# Design, Synthesis of Novel Quinazolinone Derivatives and Evaluation of EGFR Kinase Inhibition Activity via In Vitro and In Silico Studies

**DOI:** 10.1111/cbdd.70338

**Published:** 2026-06-19

**Authors:** Aybüke Züleyha Kaya, Asaf Evrim Evren, Gülşen Akalın‐Çiftçi, Leyla Yurttaş

**Affiliations:** ^1^ Faculty of Pharmacy, Department of Pharmaceutical Chemistry Anadolu University Eskisehir Turkey; ^2^ Institute of Graduate Education Anadolu University Eskisehir Turkey; ^3^ Faculty of Pharmacy, Department of Biochemistry Anadolu University Eskisehir Turkey

**Keywords:** anticancer, apoptosis induction, caspase‐3 enzyme inhibition, EGFR inhibition, molecular dynamics simulations, quinazolinone

## Abstract

Cancer is one of the most seen diseases worldwide. Due to the serious side effects of anticancer drugs and toxic effects on healthy cells, investigations for novel anticancer agents with low side effects have become one of the most important research areas of our age. With this objective, 17 new compounds were designed and successfully synthesized based on the quinazolinone, which is commonly found in the structures of FDA‐approved EGFR inhibitors, and their structures were elucidated by IR, HRMS, ^1^H, and ^13^C‐NMR spectral analysis methods. According to cytotoxicity test results, it was determined that compound **4j** showed high inhibitory activity compared to cisplatin and gefitinib in the A549 cell line. In apoptosis induction studies, it was observed that compounds **4a**, **4b**, **4d**, and **4f** showed activity in the A549 cell line, while compound **4j** showed activity in the MCF‐7 cell line. In the study investigating caspase‐3 activation, compounds **4a**, **4m**, and **4o** were found active in the A549 cell line, while compounds **4a** and **4o** were found active in MCF‐7 cells. In EGFR enzyme inhibition studies, it was observed that the activities of molecules **4b**, **4f**, and **4j** were higher than gefitinib. In silico studies indicated that **4j** interacted by establishing significant bonds with both caspase‐3 and EGFR enzyme.

## Graphical abstract

Seventeen new quinazolinone derivatives were synthesized in pure form. Compound **4j** showed the highest inhibitory activity against the A549 cell line and induced apoptosis via caspase‐3 activation in MCF‐7 cells. **4j** inhibited EGFR enzyme twice as effectively as gefitinib. In silico studies confirmed interactions with key residues in both enzymes.

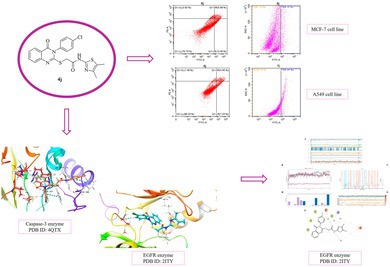

## 1 Introduction

Cancer is one of the most prevalent diseases throughout history. Cancer is the result of uncontrolled abnormal cells divisions. Leading cause of cancer is mutations that damage the biological processes of cells such as metabolic functions and growth (Brown et al. 2023; Evan and Vousden 2001; Harris and McCormick 2010; Peltomaki 2012). Not only mutations caused cancer, but several different factors directly or indirectly caused cancer. Some of these factors are viruses, bacteria, radiation, environmental factors, genetics, unhealthy lifestyle, stress, and chemicals (Adjiri 2016; Blackadar 2016; Kaya et al. 2025; Majérus 2022; Vineis and Wild 2014). Cancer is a serious public health issue due to high mortality rate, difficulty and expense of treatment, and high incidence of cancer (Forma et al. 2025; Kim et al. 2025; Tsokkou et al. 2025). The location of cancer cells, structural characteristics and size of the tumor, determine whether treatment needs to be regional or systemic. Radiotherapy, chemotherapy, and invasive procedures are commonly used approaches to treating cancer (Feller et al. 2025; Hua et al. 2025; Yun et al. 2025; Zafar et al. 2025). Hormone therapy and gene therapy were introduced as effective treatment methods with advancement of technology and clinical studies (Cetin et al. 2025; Shahdeo et al. 2025; Shokoohi et al. 2025; Tripathi et al. 2025). Main goal of the chemotherapy is to inhibit tumor growth and prevent the metastasis of cancerous cells. However, severe side effects of the chemotherapeutic complicate the treatment processes. In addition, anticancer drugs exhibit cytotoxic activity to noncancerous cells beside cancer cells. Researchers have focused on designing and synthesizing novel drug candidates that have low side effects and are selective to the cancerous cells.

Epidermal growth factor receptors (EGFR) are known as tyrosine kinase inhibitors (TKI) because they block kinase activity (Zubair and Bandyopadhyay 2023). EGFR inhibitor drugs are attracting attention in targeted therapy studies. EGFR activation is a result of the conformational changes caused by external binding of the ligand to the cell (Mudumbi et al. 2024; Tariq et al. 2025; Wirth et al. 2023; Zubair and Bandyopadhyay 2023). As a result of the changes, disruptions are observed in essential cell functions such as cell growth and proliferation (Tariq et al. 2025; Wang et al. 2024). Over‐expression of the EGFR enzyme is frequently encountered in head, neck, lung, breast, and pancreas cancer pathogenesis (Constantin et al. 2024; Mangla et al. 2024; Shyamsunder et al. 2025; Zhou et al. 2025). Moreover, high expression of EGFR promotes invasiveness of tumors and metastasis. EGFR inhibitors were first approved as non‐small cell lung cancer (NSCLC) drugs by the FDA from 2003 to 2004 (Harada and Ono 2024).

**FIGURE 1:**
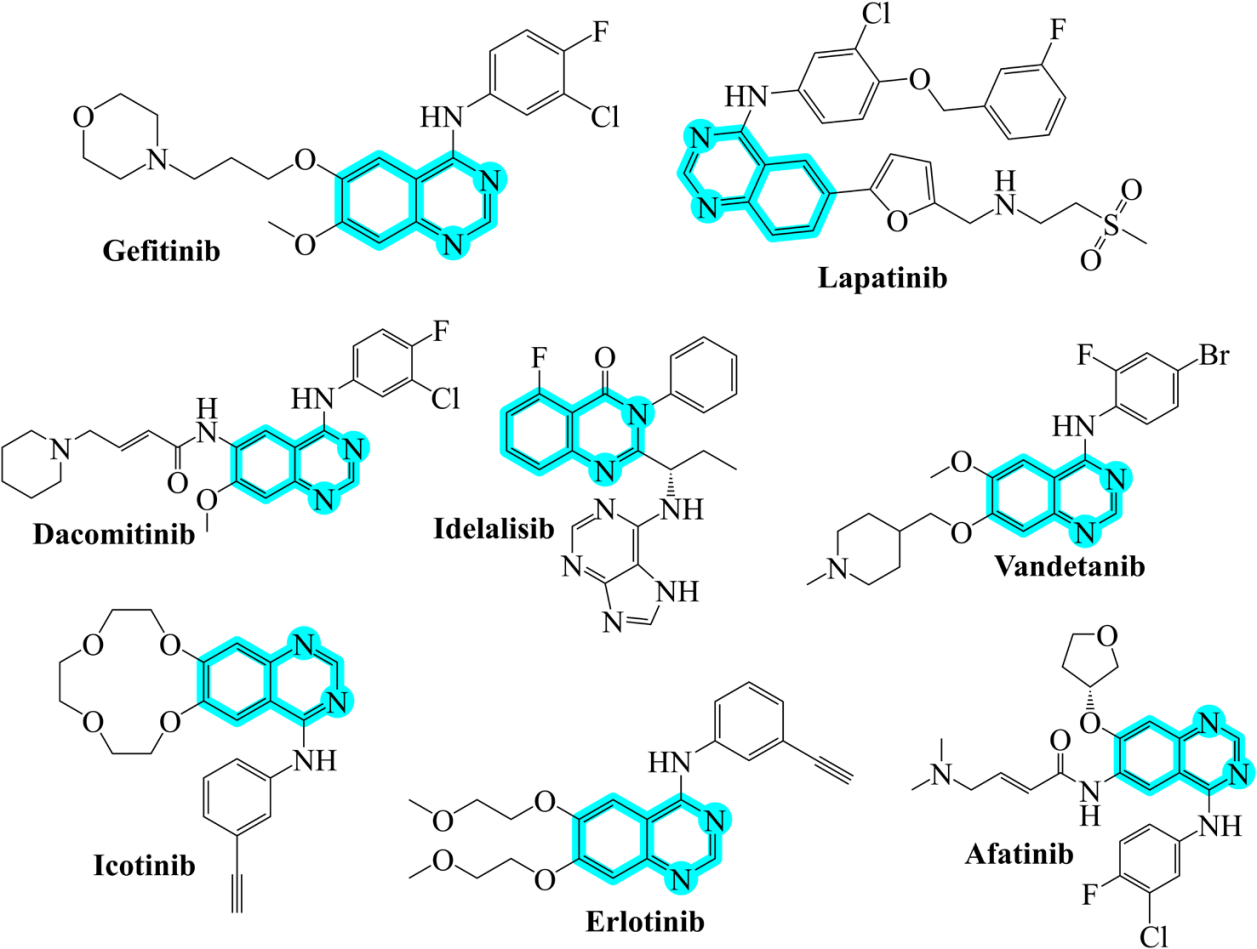
Tyrosine kinase inhibitors containing quinazoline/quinazolinone rings.

Quinazoline/quinazolinone rings are one of the major classes of heterocyclic compounds. Drugs and drug candidates containing quinazolinone have a variety of biological activities like anticancer, antimalarial, anticonvulsant, anti‐inflammatory, antihypertensive, and antiviral (Alsibaee et al. 2023; Bala et al. 2024; Deng et al. 2025; Tokali 2025). Numerous investigations have been conducted on the potential effects of quinazoline/quinazolinone derivatives, particularly in cancer treatment. Majority of tyrosine kinase inhibitory drugs featuring quinazoline/quinazolinone scaffolds in their structure. Quinazoline/quinazolinone derivatives stand out as compounds that can inhibit the growth of cancer cells and target abnormal signaling pathways in these cells. In 2003, the FDA approved Gefitinib, the first TKI drug containing a quinazoline ring, specifically to treat NSCLC. Drugs approved for clinical use include lapatinib, afatinib, vandetanib, dacomitinib, idelalisib, icotinib, and erlotinib, as presented in Figure 1.

The significance of quinazoline/quinazolinone structures in anticancer drug synthesis has been commonly mentioned in the literature. To provide a new perspective to treatment of cancer, 17 novel compounds with quinazoline/quinazolinone scaffolds have been designed and synthesized. Their anticancer activities have been investigated, and the results aligned with in silico assessments.

## 2 Results and Discussion

### 2.1 Chemistry

**FIGURE 2:**
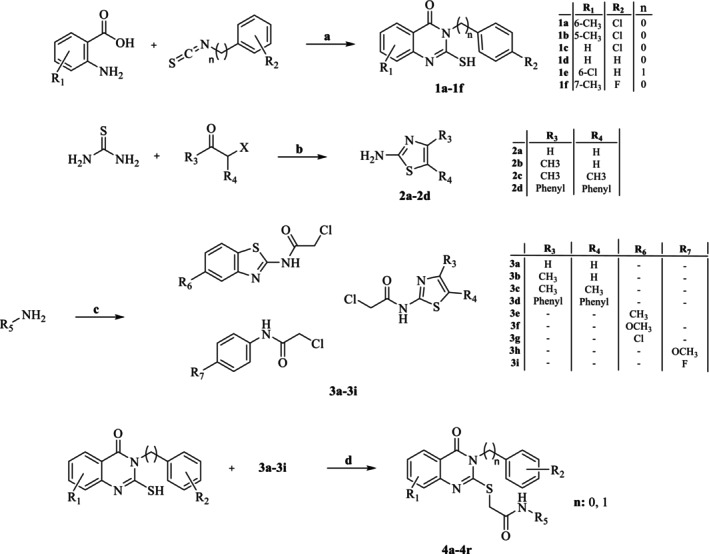
Synthesis scheme of final products *Experimental conditions: (a) absolute EtOH, triethylamine, reflux, 6 h; (b) EtOH, r.t., 2 h; (c) tetrahydrofuran, triethylamine, triethylamine, chloracetyl chloride, 0°C, 30 min.‐1 h, then r.t.; (d) acetone, potassium carbonate, r.t., 1 h.

Seventeen novel quinazolinone derivatives are synthesized according to the synthesis route given in Figure 2 and Table 1. Spectral analysis of resulted compounds confirmed their structures (Spectra of analysis given as Figures S1 **–** S63). When the IR spectra are examined, it is observed that stretching bands of aromatic C‐H bands in the structure are between 3279 and 3005 cm^−1^, stretching bands of aliphatic C‐H bonds are between 2988 and 2731 cm^−1^, C=C stretching bands in quinazolinone ring and other cyclic structures substituted to this ring are between 1609 and 1466 cm^−1^, C=O stretching bands are between 1707 and 1661 cm^−1^, out of plane bands of the **4a–4m** derivatives with a 1,4‐disubstituted benzene ring are between 866 and 820 cm^−1^, and out of plane deformation bands of 1,3,5‐trisubstituted benzene in the quinazolinone ring structure are between 812 and 719 cm^−1^, all within the expected regions. The final molecules contain quinazolinone, thiazole, benzothiazole, and substituted benzene rings in their structure. Examination of NMR spectra of similar structures in the literature revealed similarities with the spectra of the resulting products (Heil et al. 2013; Sohrabi et al. 2022). ^1^H‐NMR spectra showed that in the **4a–4g**, **4r** derivatives, the peaks of the methyl protons at positions 5, 6, and 7 of the quinazolinone ring were seen as singlet peaks in between 2.39 and 2.69 ppm. In structures with acetylated substitutions at the 3rd position of the quinazolinone ring, the peaks of the NH protons, common to the quinazolinone ring, were seen as singlet peaks in between 10.46 and 12.82 ppm, while the peaks of CH_2_ protons attached to the sulfur atom were observed as singlets in the range of 4.11–4.28 ppm. In the spectra of compounds with thiazole derivatives substituted at the 4/5th position, peaks of protons belonging to the thiazole ring were detected as doublet peaks ranging from 6.74 to 7.50 ppm, while peaks of methyl groups substituted to the thiazole ring were seen as singlet peaks between 2.16 and 2.69 ppm. In the spectra of derivatives with benzothiazole rings substituted, the peaks of the protons belonging to ring were detected as doublets and double doublets between 7.04 and 8.10 ppm, while peak of OCH_3_ group proton substituted to ring at the 6th position was seen as a singlet at 3.79 ppm. When the ^13^C‐NMR spectra of the synthesized molecules were examined, several peaks equivalent to the number of carbon atoms in the structures of the molecules were observed. The aliphatic carbon peaks seen between 10.81 and 47.70 ppm, while the carbonyl carbon of the acetylated products substituted at the 3rd position of the quinazolinone ring, showed peaks between 165.88 and 179.72 ppm. When the obtained spectral results were compared with similar structures in the literature, it was observed that the peaks were in similar regions (Heil et al. 2013; Sohrabi et al. 2022).

**TABLE 1:** Synthesized compounds.

Compounds	*n*	R_1_	R_2_	R_5_
**4a**	0	6‐CH_3_	4‐Cl	Thiazole‐2‐yl
**4b**	0	6‐CH_3_	4‐Cl	4‐Methylthiazole‐2‐yl
**4c**	0	6‐CH_3_	4‐Cl	6‐Methylbenzothiazole‐2‐yl
**4d**	0	6‐CH_3_	4‐Cl	6‐Chlorobenzothiazole‐2‐yl
**4e**	0	5‐CH_3_	4‐Cl	Thiazole‐2‐yl
**4f**	0	5‐CH_3_	4‐Cl	4,5‐Dimethylthiazole‐2‐yl
**4g**	0	5‐CH_3_	4‐Cl	4,5‐Diphenylthiazole‐2‐yl
**4h**	0	5‐CH_3_	4‐Cl	6‐Methylbenzothiazole‐2‐yl
**4i**	0	H	4‐Cl	Thiazole‐2‐yl
**4j**	0	H	4‐Cl	4,5‐Dimethylthiazole‐2‐yl
**4k**	0	H	4‐Cl	6‐Methylbenzothiazole‐2‐yl
**4l**	0	H	4‐Cl	6‐Methoxybenzothiazole‐2‐yl
**4m**	0	H	4‐Cl	6‐Chlorobenzothiazole‐2‐yl
**4n**	1	H	H	Thiazole‐2‐yl
**4o**	1	H	H	4‐Fluorophenyl
**4p**	1	6‐Cl	H	4‐Fluorophenyl
**4r**	0	7‐CH_3_	4‐F	4‐Methoxyphenyl

### 2.2 Anticancer Activity Studies

#### 2.2.1 In Vitro Cytotoxicity Test Results

Cytotoxicity studies were performed on 17 final compounds (**4a–4r**), and their IC_50_ values are given in Table 2. Gefitinib and cisplatin were used as reference compounds. IC_50_ values of the compounds showed that compound **4j** exhibited the strongest inhibition in the A549 cell line. **4j** showed higher inhibitory activity than gefitinib and approximately 7 times greater than cisplatin, with IC_50_ values of 5.67 ± 2.38 μM. Although compound **4j** showed high inhibitory activity in the A549 cells, it showed lower inhibitory activity in MCF‐7 cells. In the MCF‐7, compound **4a** showed inhibitory effect with IC_50_ value of 88.44 ± 12.58 μM, while gefitinib and cisplatin showed inhibitory activity at levels of 47.83 ± 0.28 μM and 13.40 ± 2.86 μM, respectively. **4a** exhibited inhibition with IC_50_ value of 325.86 ± 28.72 μM on L929 healthy cells. Compound **4a** was found to exhibit selectivity against cancerous cells. Among the final products, compounds **4d**, **4h**, **4k**, **4m**, and **4n** showed inhibitory activity in A549 cell line, while they did not show inhibition in MCF‐7 and L929 cell lines (> 500). It was found that these compounds selectively inhibited A549 cell line and didn't have toxicity on healthy cells. Compounds **4e**, **4g**, **4i**, **4l**, **4p**, and **4r** did not show inhibition on A549 cells, while compounds **4d**, **4e**, **4g**, **4h**, **4i**, **4k**, **4l**, **4m**, **4n**, and **4r** showed no inhibition on MCF‐7 cell line.

**TABLE 2:** Results of cytotoxicity studies of the final products (**4a–4r**).

Compounds	A549^a^	MCF‐7^a^	L929^a^
**4a**	84.13 ± 0.53	88.44 ± 12.58	325.86 ± 28.72
**4b**	132.64 ± 7.35	593.40 ± 138.69	> 500
**4c**	583.04 ± 58.7	290.17 ± 54.28	> 500
**4d**	215.42 ± 56.53	> 500	> 500
**4e**	> 500	> 500	> 500
**4f**	73.59 ± 8.98	634.81 ± 210.29	512.62 ± 79.83
**4g**	> 500	> 500	> 500
**4h**	249.26 ± 73.77	> 500	> 500
**4i**	> 500	> 500	> 500
**4j**	5.67 ± 2.38	175.33 ± 27.13	130.55 ± 14.21
**4k**	373.52 ± 51.86	> 500	> 500
**4l**	> 500	> 500	> 500
**4m**	290.95 ± 91.03	> 500	> 500
**4n**	217.63 ± 71.55	> 500	> 500
**4o**	87.48 ± 11.22	329.45 ± 97.66	260.45 ± 9.32
**4p**	> 500	563.75 ± 142.16	366.50 ± 44.53
**4r**	> 500	> 500	> 500
Gefitinib	158.30 ± 16.52	47.83 ± 0.28	—
Cisplatin	43.61 ± 1.05	13.40 ± 2.86	—

^a^ The values given in the table are in μM. Red font represents the most active compounds' cytotoxicity values.

Based on the results of the MTT test, it was found that relatively active compounds such as **4a**, **4j**, **4f**, and **4o**, contains small substitutions (methyl, dimethyl, or fluoro group) in their structure. On the other hand, compound **4g**, which has diphenyl substitution in its structure, did not exhibit any activity on both cell line. This results indicated that bulkier groups added to the structure of the compounds cytotoxic activity decreases. When smaller groups are substituted to the thiazole or phenyl ring, cytotoxic activity is observed.

#### 2.2.2 Apoptosis Induction (Flow Cytometric Method)

**FIGURE 3:**
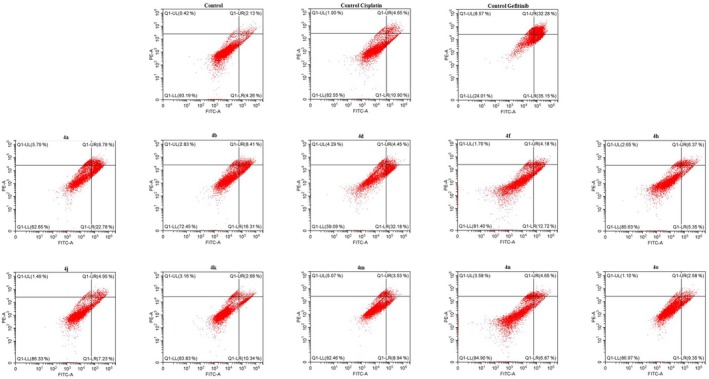
Apoptotic activity graphics of control group, standard drugs (cisplatin and gefitinib) and compounds **4a**, **4b**, **4d**, **4f**, **4h**, **4j**, **4k**, **4m**, **4n**, and **4o** (in order in the diagram) on A549 cell line.

**FIGURE 4:**
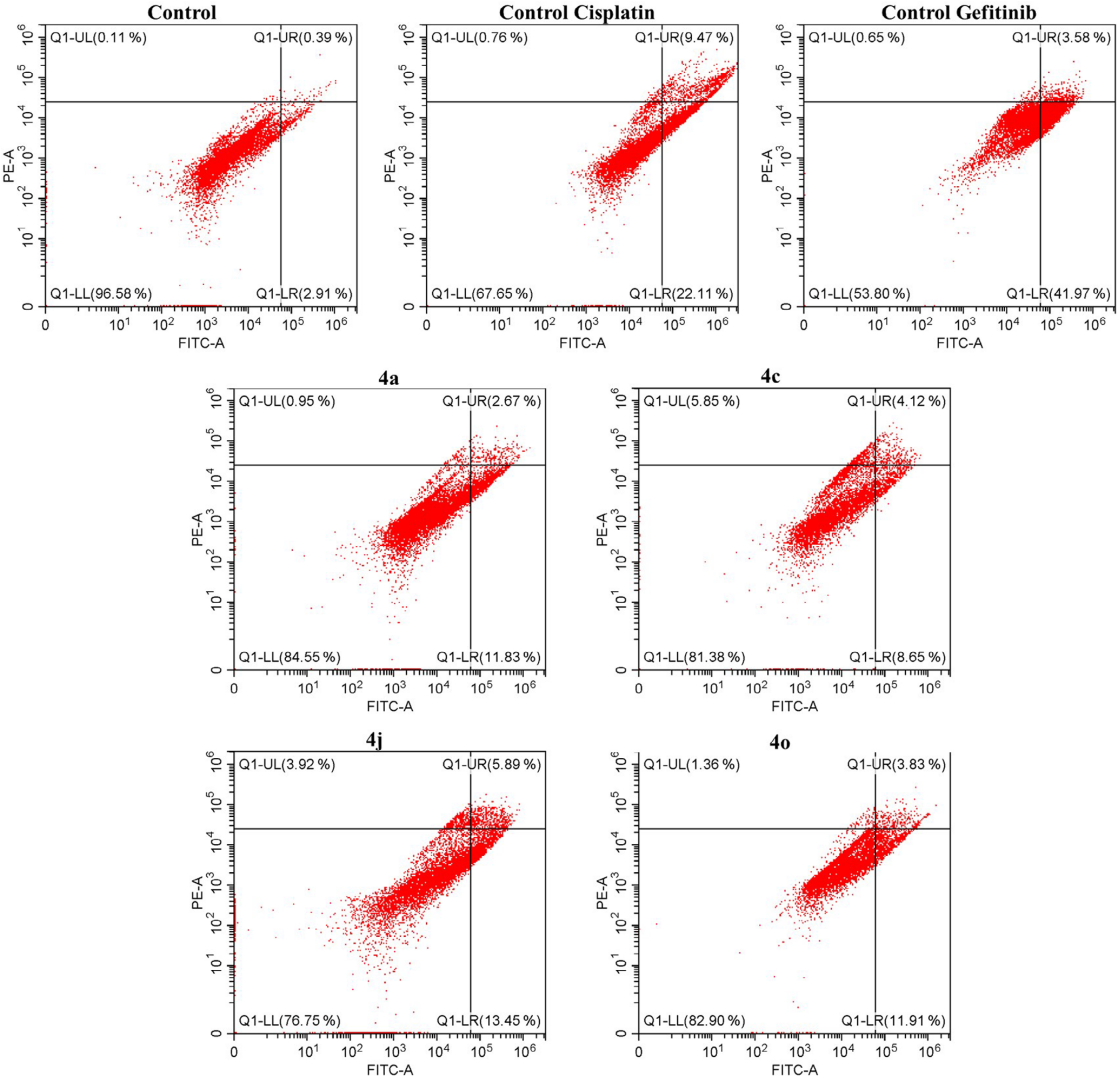
Apoptotic activity graphics of control group, standard drugs (cisplatin and gefitinib), and compounds **4a**, **4c**, **4j**, and **4o** (in order in the diagram) on MCF‐7 cell line.

Based on the results of cytotoxicity study, effective compounds compare the standard drug was chosen. Apoptotic activities of the chosen compounds investigated via Annexin‐V FITC analysis method. Analysis results on A549 cell line shown in Figure 3 and Table 3 and analysis results on MCF‐7 cell line shown in Figure 4 and Table 4.

**TABLE 3:** Apoptotic activity results of **4a**, **4b**, **4d**, **4f**, **4h**, **4j**, **4k**, **4m**, **4n**, and **4o** on A549 cell line.

Compounds	UL	UR	LL	LR	UR + LR
**4a**	5.79	8.78	62.65	22.78	**31.56**
**4b**	2.83	8.41	72.45	16.31	**24.72**
**4d**	4.29	4.45	59.09	32.18	**36.63**
**4f**	1.70	4.18	81.40	12.72	**16.90**
**4h**	2.65	6.37	85.63	5.35	11.72
**4j**	1.49	4.95	86.33	7.23	12.18
**4k**	3.16	2.68	83.83	10.34	13.02
**4m**	5.07	3.53	82.46	8.94	12.47
**4n**	3.58	4.65	84.90	6.87	11.52
**4o**	1.10	2.58	86.97	9.35	11.93
Control	0.42	2.13	93.19	4.26	6.39
Cisplatin	1.90	4.65	82.55	10.90	15.55
Gefitinib	8.57	32.28	24.01	35.15	67.43

Abbreviations: LL, live cells; LR, early apoptotic cells; UL, necrotic and dead cells; UR, late apoptotic cells; UR+LR, early and late apoptotic cells. Values shown in red font indicate the cytotoxicity values of the most active compounds.

**TABLE 4:** Apoptotic activity results of **4a**, **4c**, **4j**, and **4o** on MCF‐7 cell line.

Compounds	UL	UR	LL	LR	UR + LR
**4a**	0.95	2.67	84.55	11.83	14.50
**4c**	5.85	4.12	81.38	8.65	12.77
**4j**	3.92	5.89	76.75	13.45	**19.34**
**4o**	1.36	3.83	82.90	11.91	15.74
Control	0.11	0.39	96.58	2.91	3.30
Cisplatin	0.76	9.47	67.65	22.11	31.58
Gefitinib	0.65	3.58	53.80	41.97	45.55

Abbreviations: LL, live cells; LR, early apoptotic cells; UL, necrotic and dead cells; UR, late apoptotic cells; UR+LR, early and late apoptotic cells. Values shown in red font indicate the cytotoxicity values of the most active compounds.

Total early and late apoptotic cells of cisplatin and gefitinib on A549 cell line found 15.55% and 67.43%, respectively. All the analyzed compounds exhibited comparable apoptotic effect with cisplatin. While **4f** has shown nearly same activity (16.90%) with cisplatin, **4a**, **4b**, and **4d** have shown higher activity than cisplatin with 31.56%, 24.72%, and 36.63%, respectively. **4d** has exhibited 2.3 times higher apoptotic effect (36.63%) than cisplatin (15.55%).

Results of the apoptosis induction study indicated that total early and late apoptotic cells of cisplatin and gefitinib on MCF‐7 cell line found 31.58% and 45.55%, respectively. Apoptotic activities of **4a**, **4c**, and **4o** have been detected 14.50%, 12.77%, and 15.74%, respectively. **4j** has shown the highest apoptotic activity with 19.34%.

#### 2.2.3 Caspase‐3 Enzyme Activation

**FIGURE 5:**
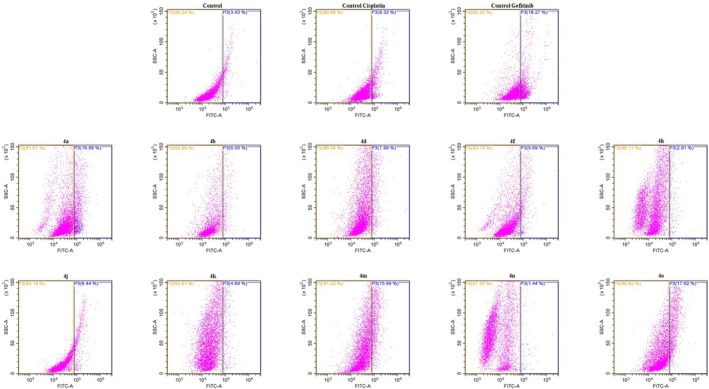
Caspase‐3 enzyme activation graphics of control group, standard drugs (cisplatin and gefitinib), and compounds **4a**, **4b**, **4d**, **4f**, **4h**, **4j**, **4k**, **4m**, **4n**, and **4o** (in order in the diagram) on A549 cells.

**FIGURE 6:**
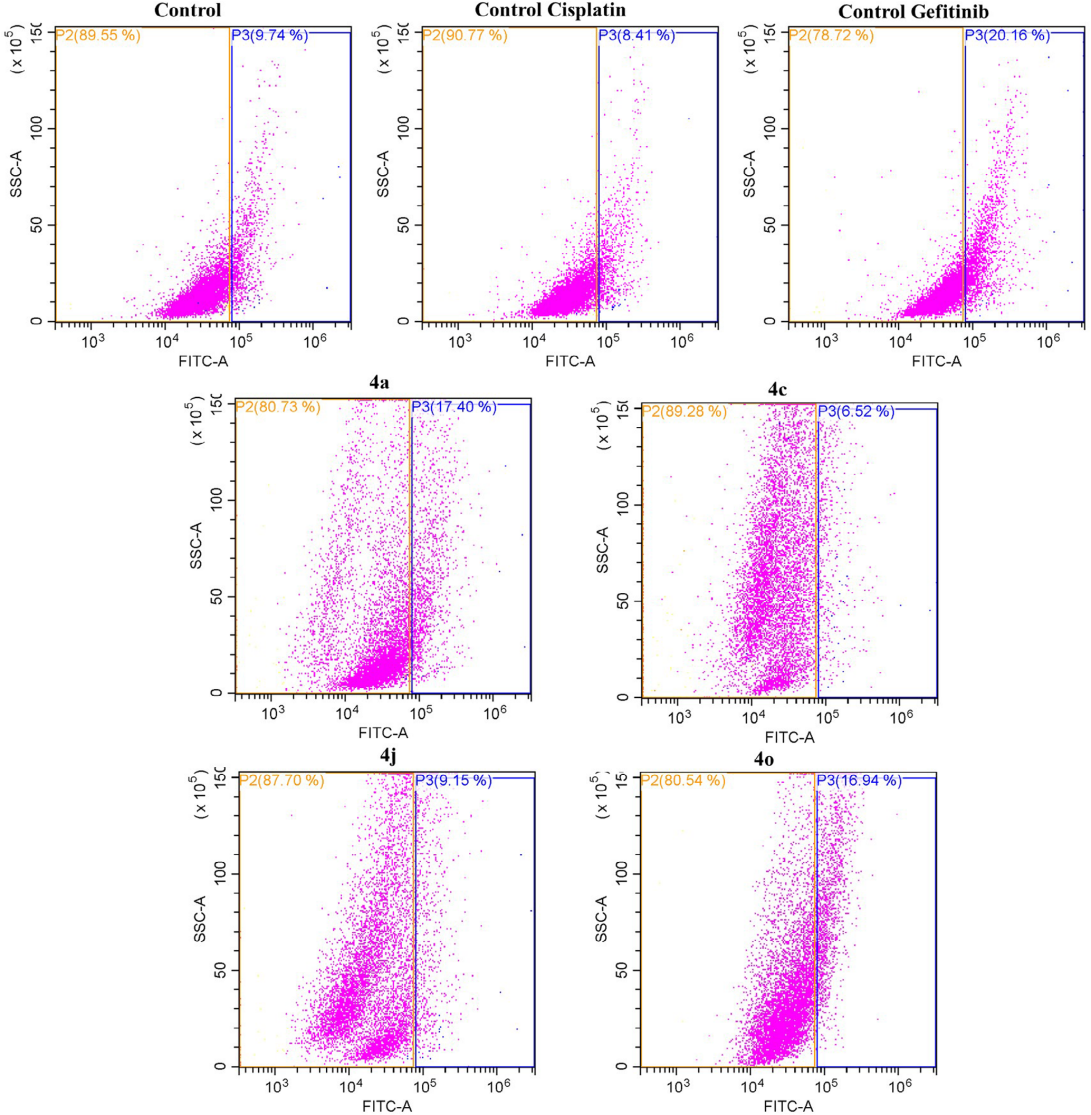
Caspase‐3 enzyme activation graphics of control group, standard drugs (cisplatin and gefitinib), and compounds **4a**, **4c**, **4j**, and **4o** (in order in the diagram) on MCF‐7 cell line.

Apoptosis induction studies have been evaluated and compounds that have apoptotic effects on A549 and MCF‐7 cells have been determined. Caspase‐3 enzyme is a crossing point enzyme which intrinsic and extrinsic pathways crossed. To determine the pathway of the compounds that showed apoptotic effects, caspase‐3 enzyme was chosen. The caspase‐3 activation percentage of negative caspase‐3 and positive caspase‐3 on A549 and MCF‐7 cells is presented in Tables 5 and 6. Also, caspase‐3 enzyme activation diagrams are presented in Figures 5 and 6.

**TABLE 5:** % Caspase‐3 activation results of **4a**, **4b**, **4d**, **4f**, **4h**, **4j**, **4k**, **4m**, **4n**, and **4o** on A549 cells.

Compounds	% Negative Caspase‐3 P (%)	% Positive Caspase‐3 P (%)
**4a**	81.67	**16.99**
**4b**	92.86	6.50
**4d**	89.94	7.99
**4f**	93.70	5.69
**4h**	96.11	2.91
**4j**	93.14	6.44
**4k**	92.61	4.84
**4m**	81.22	**15.94**
**4n**	97.20	1.44
**4o**	80.82	**17.62**
Cisplatin	89.88	9.32
Gefitinib	80.20	18.27
Control	96.24	3.43

*Note:* Values shown in red font indicate the cytotoxicity values of the most active compounds.

**TABLE 6:** % Caspase‐3 Activation results of **4a**, **4c**, **4j**, and **4o** on MCF‐7 cell line.

Compounds	% Negative Caspase‐3 P (%)	% Positive Caspase‐3 P (%)
**4a**	80.73	**17.40**
**4c**	89.28	6.52
**4j**	87.70	9.15
**4o**	80.54	**16.94**
Cisplatin	90.77	8.41
Gefitinib	78.72	20.16
Control	89.55	9.74

*Note:* Values shown in red font indicate the cytotoxicity values of the most active compounds.

Caspase‐3 enzyme activation of cisplatin and gefitinib on A549 cell line was found 9.32% and 18.27%, respectively. Starting from the most active compound, **4o**, **4a**, and **4m** showed activation with 17.62%, 16.99%, and 15.94%, respectively. Results indicated that **4o**, **4a**, and **4m** exhibited two times higher activation than cisplatin and comparable activation with gefitinib. **4h** and **4n** did not show any significant activation and were considered non‐effective.

The percentage of the caspase‐3 enzyme activations of cisplatin and gefitinib on MCF‐7 cell line determined 8.41% and 20.16%, respectively. **4a** and **4o** exhibited two times higher caspase‐3 activation than cisplatin with a percentage of 17.40% and 16.94%, respectively. Even though **4a** and **4o** did not show superior activation to gefitinib, they still exhibited relatively close activation with gefitinib. Also, **4c** and **4j** showed nearby activity with cisplatin at a percentage of 6.52% and 9.15%, respectively.

#### 2.2.4 EGFR Enzyme Inhibition

EGFR enzyme inhibition of compounds **4a**, **4b**, **4f**, and **4j** was investigated. Results of the EGFR inhibition study are shown in Table 7. Gefitinib was chosen as the standard drug. IC_50_ value of gefitinib was determined to be 63.09 nM. Results indicated that **4b**, **4f**, and **4j** exhibited superior inhibition on the EGFR enzyme than gefitinib. While **4b** and **4f** showed 25 times higher inhibition than gefitinib, **4j** also showed two times higher inhibition than gefitinib.

**TABLE 7:** EGFR inhibition results of **4a**, **4b**, **4f**, and **4o**.

Compounds	IC_50_ (nM)
**4a**	> 100,000
**4b**	**2.15**
**4f**	**2.61**
**4j**	**35.67**
**4o**	> 100,000
Gefitinib	63.09

*Note:* Values shown in red font indicate the cytotoxicity values of the most active compounds.

### 2.3 In Silico Approaches

#### 2.3.1 Molecular Docking Analysis of Caspase‐3 Enzyme

**FIGURE 7:**
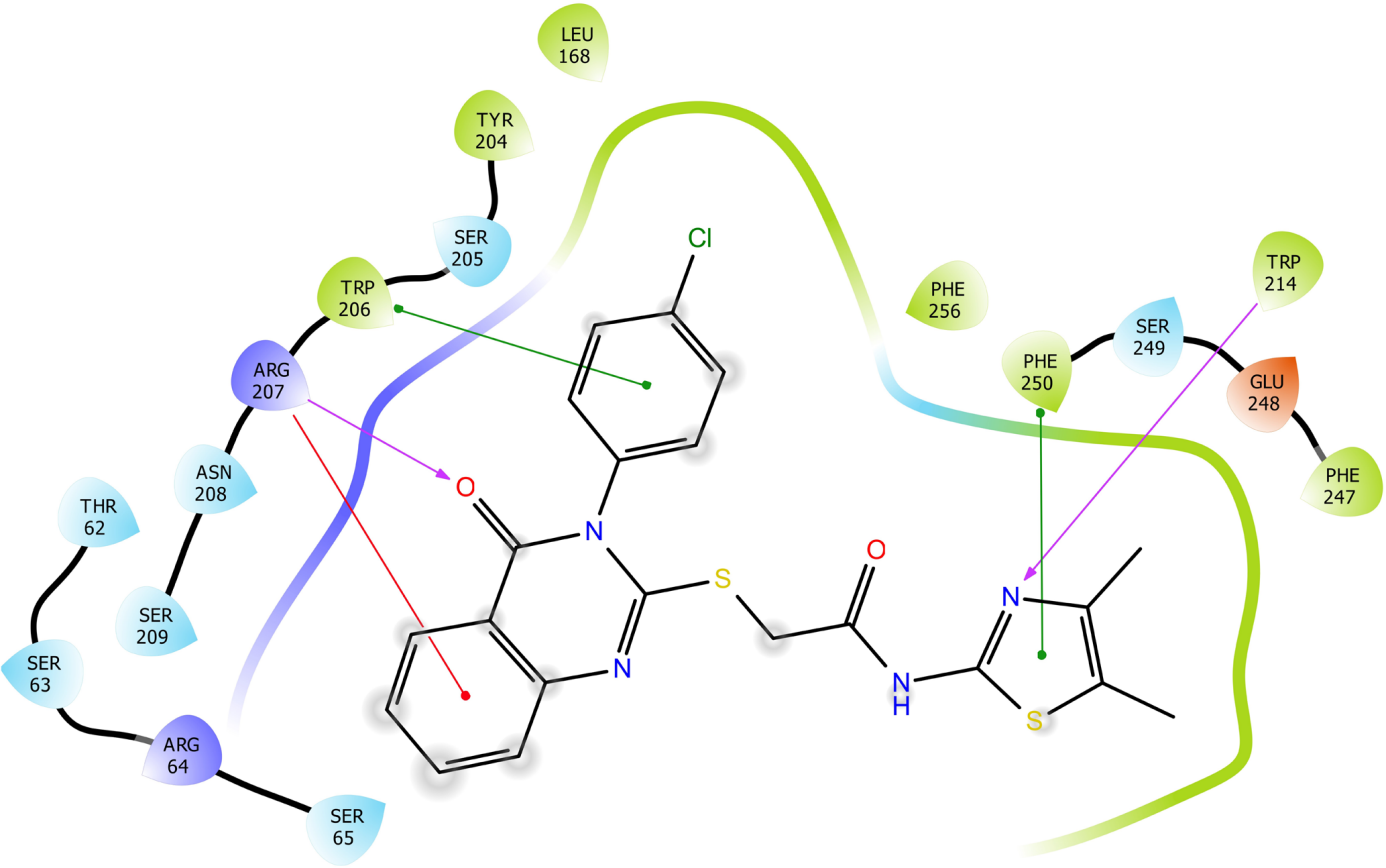
Two‐dimensional (2D) molecular docking poses of **4j**‐caspase‐3 complex.

**FIGURE 8:**
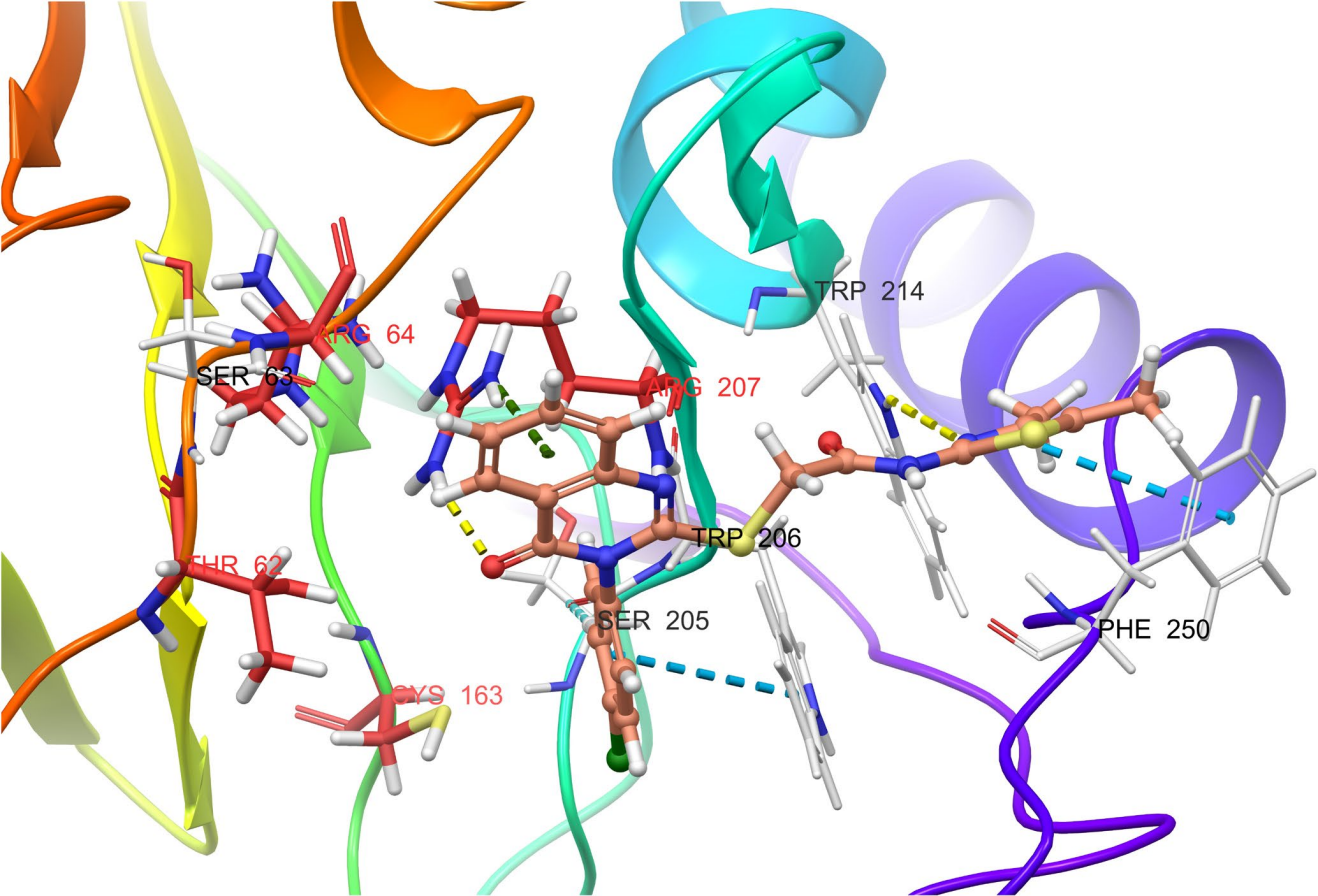
Three‐dimensional (3D) molecular docking poses of **4j**‐caspase‐3 complex.

MTT, caspase‐3 inhibition and EGFR inhibition test results indicated that compound **4j** exhibit highest inhibitory activity on EGFR enzyme. Primarily focus of this study is to find molecules to inhibit EGFR enzyme. Aim of the docking study was to explained the representative pattern of the interactions. For this purpose, **4j**, which is most active compound on EGFR inhibition test, has chosen to be docked. Two and three dimensional interaction poses of **4j**‐caspase‐3 enzyme complex were given in Figures 7 and 8. Interactions with Arg207, Trp206, Cys163, Arg64, Ser120, His121, Gly122, Tyr204, Gln161, Ser205, and Phe250 amino acid residues is important to exhibited caspase‐3 enzyme activation. Results showed that **4j**'s carbonyl oxygen of quinazolinone ring with Arg207 and thiazole nitrogen with Trp214 interacted via hydrogen bonds. Also, quinazolinone ring formed a π‐cation bond with Arg207. Additionally, 4‐chlorophenyl ring with Trp206 and thiazole ring with Phe250 amino acid residues interacted via π‐π interactions.

#### 2.3.2 Molecular Docking Study on EGFR

**FIGURE 9:**
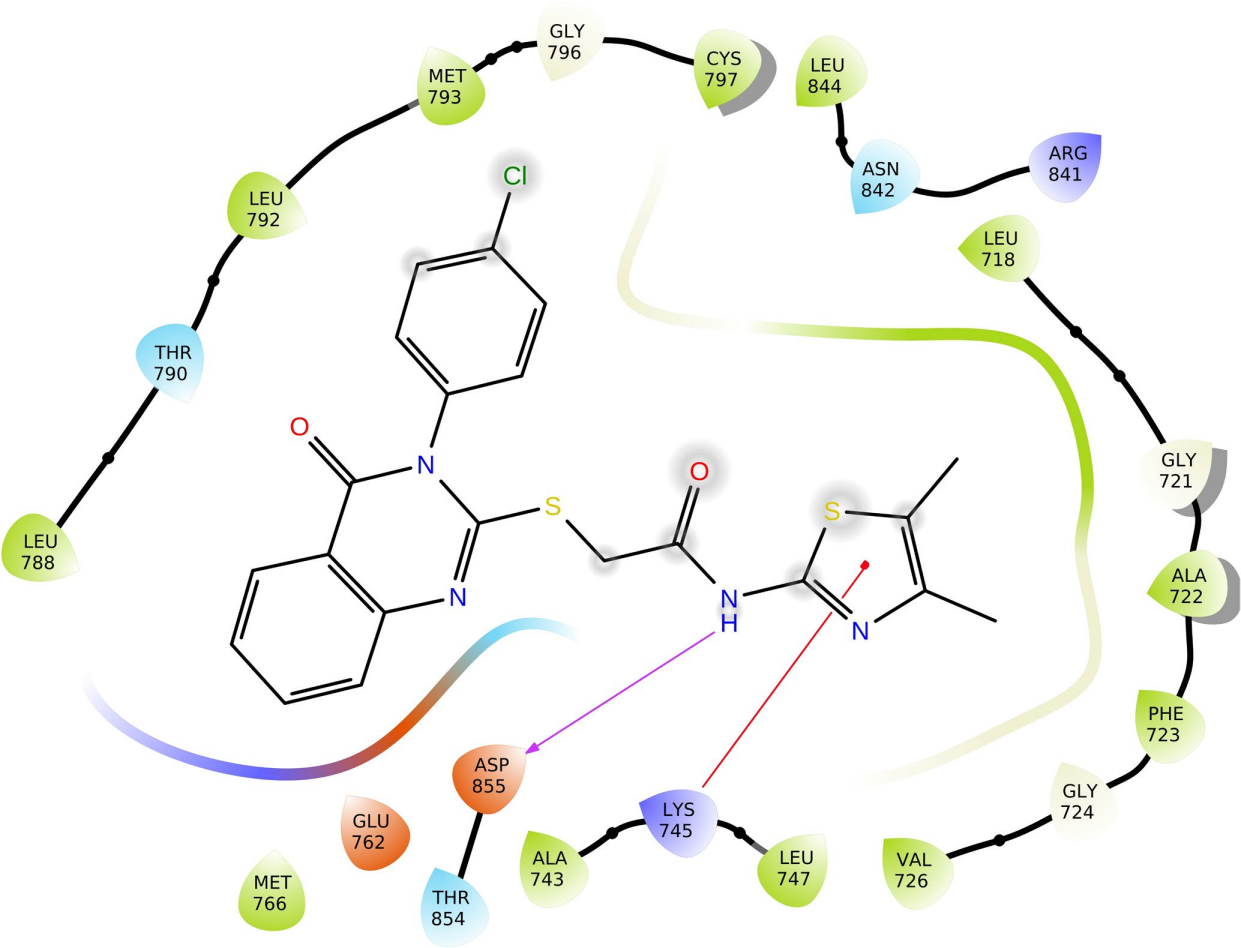
2D molecular docking poses of **4j**‐EGFR enzyme complex.

**FIGURE 10:**
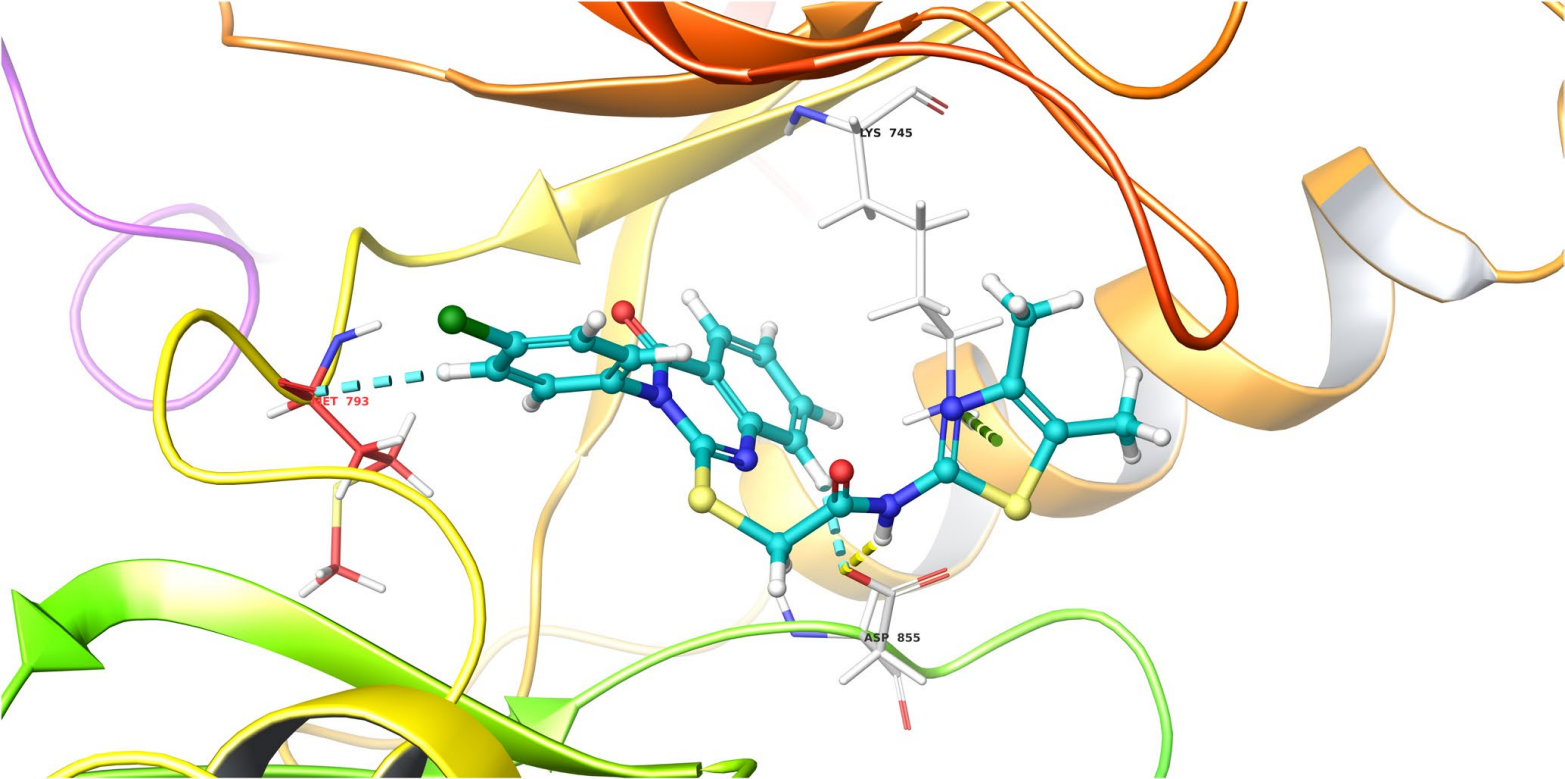
3D molecular docking poses of **4j**‐EGFR enzyme complex.

2D and 3D interaction poses of the **4j**‐EGFR enzyme complex were shown in Figure 9 and Figure 10. Amide structure between quinazolinone and thiazole ring formed hydrogen bonds with Asp855 amino acid. Also, the thiazole ring interacted with Lys745 amino acid via π‐cation bond. Based on the 3D poses of **4j**‐EGFR enzyme complex, the 4‐chlorophenyl ring interacted with Met793 via hydrogen bond. This indicates that **4j** interacts with the allosteric site of the EGFR. Interaction with Met793 is important to exhibit the inhibitory activity. Met793 amino acid residue is placed in the hinge structure of the enzyme. FDA‐approved drugs like gefitinib showed their inhibitory activities via interaction with Met793 residue.

#### 2.3.3 Molecular Dynamics Simulations of EGFR Enzyme‐**4j** Complex

**FIGURE 11:**
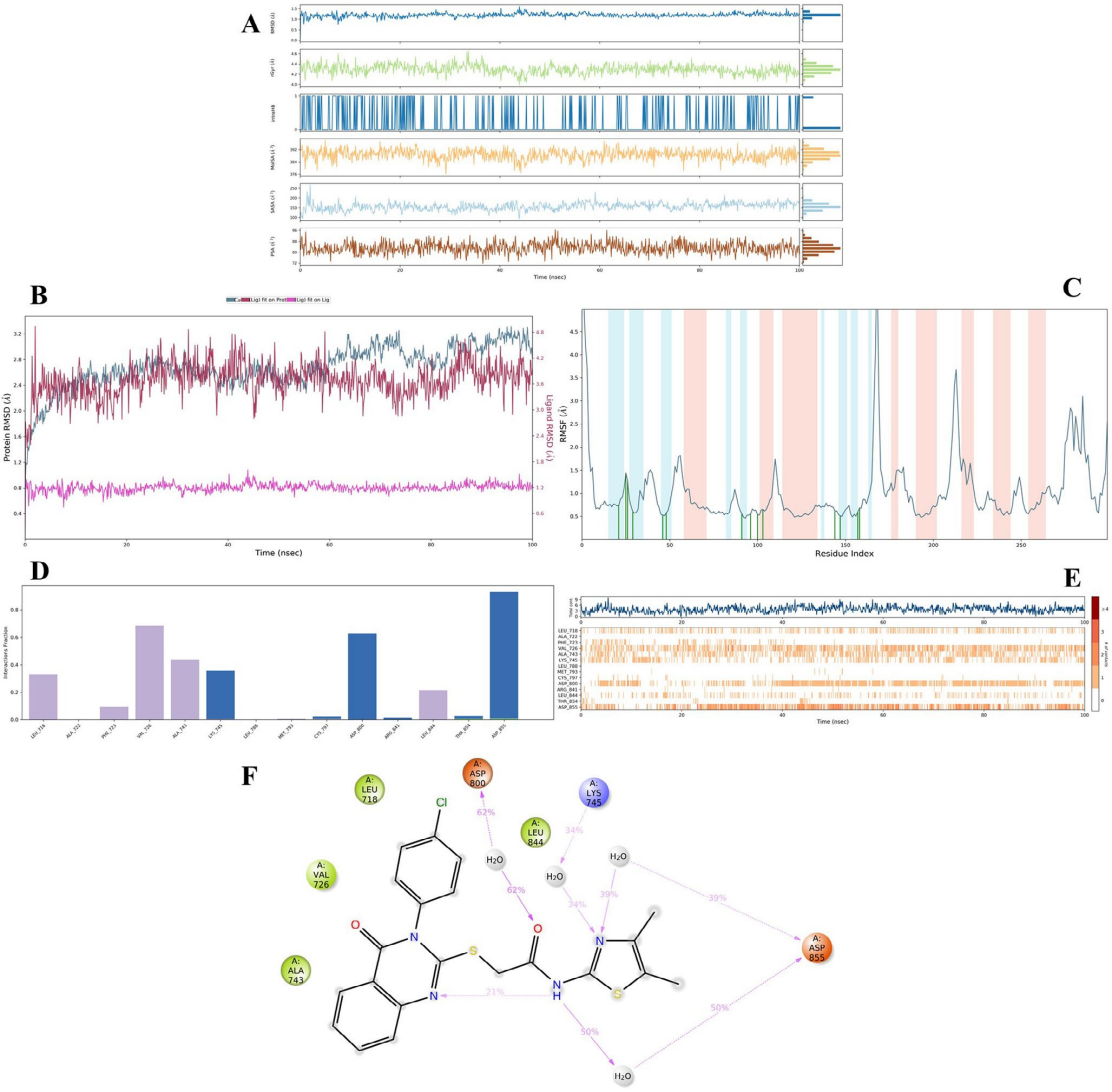
Stability and interaction diagrams of **4j**‐EGFR enzyme complex * (A) Physicochemical properties of ligand, (B) RMSD graphics of ligand and protein through simulation, (C) RMSF diagram of interacted amino acids, (D) Diagram of fraction‐residue interaction, (E) Graphic of interactions‐interaction types‐time, (F) 2D poses of amino acid‐compound interactions.

Molecular dynamics (MD) simulation was performed to determine the interactions between the **4j**‐EGFR enzyme complex and the stability of the interactions. MD simulations were carried out for 100 ns for clear observation. Results of the MD simulations are given in Figure 11.

Based on the results of Figure 11A–C, Rg values were observed at 0.5 Å. When RMSD graphic investigated values determined between 1 and 3 Å in correlation with the literature. In the RMSF graph showing interactions occurring in the enzyme's loop region, peaks below 1 Å were observed for amino acids interacting with **4j**.

When MD simulations graphics on Figure 11D–F and video (link in Acknowledgements section) investigated, it was observed that **4j** interacted with Leu718, Phe723, Val726, Ala743, Ile744, Lys745, Ile789, Leu792, Met793, Cys797, Asp800, Arg841, Leu844, Thr854, and Asp855 amino acids. Lys745 with thiazole nitrogen via water dependent hydrogen bond (34%), Asp800 with carbonyl oxygen via water dependent hydrogen bond (62%), Asp855 with thiazole nitrogen (39%), and amide nitrogen (50%) via water dependent hydrogen bond. Additionally, compound **4j** has been found to interact with many amino acids: Leu718 (hydrophobic bond), Val726 (hydrophobic bond), Ala743 (hydrophobic bound), Lys745 (water dependent hydrogen bond, hydrophobic bond), Asp800 (water dependent hydrogen bond), Leu844 (hydrophobic bond), and Asp855 (water dependent hydrogen bond, hydrogen bond). The simulation revealed continuous interaction between the Asp855 amino acid residue and compound **4j** in the 20–80 ns range. Two‐dimensional poses of EGFR‐**4j** interactions showed that compound **4j** forms intramolecular hydrogen bonds. Intramolecular hydrogen bonds increase the stability of the structure.

## 3 Material and Methods

### 3.1 Chemistry

All chemicals supplied from Merck (Germany) and Sigma Aldrich (USA) companies. No further purifications have been done. Synthesis reactions were monitored by using thin layer chromatography (TLC). As mobile phase, ethyl acetate: petroleum ether was chosen at 3:1 and 9:1 rates. According to the results of the TLC analysis, reactions were terminated. Melting points of the final products were detected via Cole Parmer MP‐250 melting point device and not corrected. Structural analysis of the compounds was conducted by using IR, HRMS, ^1^H and ^13^C‐NMR techniques. Shimadzu‐IR (Japan) analysis device was used to obtain IR spectra. NMR analysis was performed via 300 MHz and 75 MHz Bruker spectrometer (USA). Compounds were dissolved in deuteron dimethyl sulfoxide and spectra were recorded by comparison with trimethyl silane (TMS). HRMS analysis of the compounds was detected via LC/MS‐IT‐TOF (Japan) system. M + 1 values of compounds were found similar to calculated molecular weights.

**Synthesis of 3‐aryl‐5/6‐methyl/chloro‐2‐mercaptoquinazolin‐4( *3H* )‐one (1)**

Anthranilic acid derivative (1 eq) and substituted phenyl/benzyl isothiocyanate derivatives (1 eq) were dissolved in 50 mL absolute ethanol. Triethylamine (TEA, 1.5 eq) was added to the reaction as a catalyst and refluxed for 6 h. Termination of the reaction was detected by TLC analysis. Solid product was filtered and recrystallized from absolute ethanol (Shafik et al. 2016).

**Synthesis of 4/5‐disubstitued‐thiazole‐2‐amine (2a–2d)**

Thiourea (1 eq) and 3‐chloro‐2‐butanone (1 eq) dissolved in 25 mL ethanol and stirred at room temperature for 2 h. Termination of the reaction tracked by TLC analysis. Solid product filtered and recrystallized from ethanol. Same method applied to obtain two different thiazole derivatives with 2‐bromo‐1,2‐diphenylethan‐1‐one and chloroacetone (Asaf Evrim Evren et al. 2021; Walter et al. 2010).

**Synthesis of N‐(substituted thiazole‐2‐yl/benzothiazole‐2‐yl/phenyl)‐2‐chloroacetamides (3a–3i)**

Thiazole‐2‐yl, benzothiazole‐2‐yl and aniline derivatives (1 eq) dissolved in 50 mL tetrahydrofuran (THF). Reaction flask has been placed in the ice‐bath. 1.5 eq TEA added to the reaction dropwise. After 10 min addition of TEA, chloracetyl chloride (1.2 eq) diluted with THF started to added drop by drop. After TLC analysis, reaction terminated. Reaction solvent evaporated, then washed with water. Solid product is recrystallized from absolute ethanol (Yurttaş et al. 2016).

**Synthesis of N‐(substituted thiazole‐2‐yl/benzothiazole‐2‐yl/phenyl)‐2‐((3‐(substituted phenyl)‐5/6‐substituted‐4‐oxo‐3,4‐dihydroquinazolin‐2‐yl)thio)acetamides (4a–4r)**

3,6‐Disubstituted‐2‐mercaptoquinazolin‐4(3*H*)‐one (1 eq) and acetylated thiazole/benzothiazole/aniline derivatives (1 eq) dissolved in 25 mL acetone and stirred for 1 h at 25°C. Potassium carbonate (K_2_CO_3_, 1.5 eq) was added to the reaction mixture as a catalyst. After TLC checking, the reaction was terminated. Reaction solvent evaporated, washed with distilled water, dried at 25°C and recrystallized from absolute ethanol (Soliman et al. 2020).

**2‐((3‐(4‐Chlorophenyl)‐6‐methyl‐4‐oxo‐3,4‐dihydroquinazoline‐2‐yl)thio)‐N‐(thiazole‐2‐yl)acetamide (4a)**

C_20_H_15_ClN_4_O_2_S_2_, m.p.: 263°C. White powder. Yield: 68%. IR (cm^−1^): 1692 (C=O stretching), 820 (1,4‐disubstituted benzene ring deformation bands), 770 and 719 (1,3,5‐trisubstituted benzene ring deformation bands). ^1^H‐NMR (300 MHz, DMSO‐d_6_) δ (ppm): 2.41 (3H, s, quinazoline‐6‐CH_3_), 4.16 (2H, s, S‐CH_2_), 7.21 (1H, d, *J*: 3.53 Hz, thiazole‐CH), 7.35 (1H, d, *J*: 8.25 Hz, Ar‐CH), 7.50 (1H, d, *J*: 3.55 Hz, thiazole‐CH), 7.56 (2H, d, *J*: 8.59 Hz, Ar‐CH), 7.62 (1H, dd, *J*_*1*_: 1.92 Hz, *J*_*2*_: 8.39 Hz, Ar‐CH), 7.68 (2H, d, *J*: 8.61 Hz, Ar‐CH), 7.86 (1H, wide s, quinazolinone‐CH), 12.46 (1H, s, NH). ^13^C‐NMR (75 MHz, DMSO‐d_6_) δ (ppm): 21.19, 36.20, 114.05, 119.66, 126.19, 126.37, 130.14, 131.95, 135.22, 136.29, 136.68, 138.24, 145.56, 155.60, 158.41, 160.99, 166.69. HRMS (m/z): [M + 1]^+^ calculated 443.0398; found 443.0388.

**2‐((3‐(4‐Chlorophenyl)‐6‐methyl‐4‐oxo‐3,4‐dihydroquinazoline‐2‐yl)thio)‐N‐(4‐methylthiazole‐2‐yl)acetamide (4b)**

C_21_H_17_ClN_4_O_2_S_2_, m.p.: 250°C. Yellow powder. Yield: 61%. IR (cm^−1^): 3057 and 3005 (Aromatic C‐H stretching band), 2949 and 2916 (Aliphatic C‐H stretching band), 1680 (C=O stretching band), 1547 and 1487 (C=C stretching band), 833 (1,4‐disubstituted benzene ring deformation bands), 772 and 721 (1,3,5‐trisubstituted benzene ring deformation bands). ^1^H‐NMR (300 MHz, DMSO‐d_6_) δ (ppm): 2.27 (3H, s, thiazole‐4‐CH_3_), 2.41 (3H, s, quinazoline‐6‐CH_3_), 4.14 (2H, s, S‐CH_2_), 6.74 (1H, d, *J*: 1.03 Hz, thiazole‐CH), 7.36 (1H, d, *J*: 8.25 Hz, Ar‐CH), 7.56 (2H, d, *J*: 8.70 Hz, Ar‐CH), 7.63 (1H, dd, *J*_*1*_: 1.92 Hz, *J*_*2*_: 8.61 Hz, Ar‐CH), 7.67 (2H, d, *J*: 8.71 Hz, Ar‐CH), 7.86 (1H, wide s, quinazolinone‐CH), 12.35 (1H, s, NH). ^13^C‐NMR (75 MHz, DMSO‐d_6_) δ (ppm): 17.37, 21.18, 36.24, 108.27, 119.68, 126.24, 126.37, 130.13, 131.95, 135.21, 136.30, 136.68, 145.56, 147.18, 155.60, 161.01, 166.57. HRMS (m/z): [M + 1]^+^ calculated 457.0554; found 457.0542.

**2‐((3‐(4‐Chlorophenyl)‐6‐methyl‐4‐oxo‐3,4‐dihydroquinazoline‐2‐yl)thio)‐N‐(6‐methylbenzothiazole‐2‐yl)acetamide (4c)**

C_25_H_19_ClN_4_O_2_S_2_, m.p.: 273°C. White powder. Yield: %45. IR (cm^−1^): 3674 (N‐H stretching band), 2988 and 2901 (Aliphatic C‐H stretching band), 1688 (C=O stretching band), 1551 and 1491 (C=C stretching band), 845 (1,4‐disubstituted benzene ring deformation bands), 812 and 772 (1,3,5‐trisubstituted benzene ring deformation bands). ^1^H‐NMR (300 MHz, DMSO‐d_6_) δ (ppm): 2.40 (6H, s, CH_3_), 4.20 (2H, s, S‐CH_2_), 7.26 (1H, d, *J*: 8.59 Hz, Ar‐CH), 7.35 (1H, d, *J*: 8.26 Hz, Ar‐CH), 7.56–7.61 (3H, m, Ar‐H), 7.65–7.70 (3H, m, Ar‐H), 7.74 (1H, wide s, benzothiazole‐CH), 7.85 (1H, wide s, Quinazolinone‐CH), 12.64 (1H, s, NH). ^13^C‐NMR (75 MHz, DMSO‐d_6_) δ (ppm): 21.16, 21.43, 36.44, 119.68, 120.73, 121.79, 126.16, 126.37, 127.93, 130.15, 131.95, 132.05, 133.53, 135.21, 135.24, 136.31, 136.69, 145.54, 147.03, 155.60, 157.48, 160.99, 167.70.

***N*‐(6‐Chlorobenzothiazole‐2‐yl)‐2‐((3‐(4‐chlorophenyl)‐6‐methyl‐4‐oxo‐3,4‐dihydroquinazoline‐2‐yl)thio)acetamide (4d)**

C_24_H_16_Cl_2_N_4_O_2_S_2_, m.p.: 263°C. White powder. Yield: 43%. IR (cm^−1^): 1688 (C=O stretching band), 1549 and 1491 (C=C stretching band), 845 (1,4‐disubstituted benzene ring deformation bands), 810 and 772 (1,3,5‐trisubstituted benzene ring deformation bands). ^1^H‐NMR (300 MHz, DMSO‐d_6_) δ (ppm): 2.39 (3H, s, Quinazolinone‐CH_3_), 4.22 (2H, s, S‐CH_2_), 7.33 (1H, d, *J*: 8.27 Hz, Ar‐CH), 7.47 (1H, dd, *J*_*1*_: 2.19 Hz, *J*_*2*_: 8.64 Hz, Ar‐CH), 7.56–7.59 (3H, m, Ar‐CH), 7.68 (2H, d, *J*: 8.70 Hz, Ar‐CH), 7.78 (1H, d, *J*: 8.63 Hz, Ar‐CH), 7.85 (1H, s, Quinazolinone‐CH), 8.10 (1H, d, *J*: 2.13 Hz, benzothiazole‐CH), 12.82 (1H, s, NH). ^13^C‐NMR (75 MHz, DMSO‐d_6_) δ (ppm): 21.16, 36.44, 119.68, 121.95, 122.30, 126.12, 126.38, 126.98, 128.11, 130.15, 131.95, 133.61, 135.20, 135.26, 136.33, 136.70, 145.51, 147.96, 155.57, 159.24, 160.98, 168.15, 179.72.

**2‐((3‐(4‐Chlorophenyl)‐5‐methyl‐4‐oxo‐3,4‐dihydroquinazoline‐2‐yl)thio)‐N‐(thiazole‐2‐yl)acetamide (4e)**

C_20_H_15_ClN_4_O_2_S_2_, m.p.: 265°C. White powder. Yield: 40%. IR (cm^−1^): 3190, 3117 and 3075 (Aromatic C‐H stretching band), 2932 and 2735 (Aliphatic C‐H stretching band), 1682 (C=O stretching band), 1584, 1547 and 1493 (C=C stretching band), 831 (1,4‐disubstituted benzene ring deformation bands), 779 and 725 (1,3,5‐trisubstituted benzene ring deformation bands). ^1^H‐NMR (300 MHz, DMSO‐d_6_) δ (ppm): 2.68 (3H, s, Quinazolinone‐CH_3_), 4.15 (2H, s, S‐CH_2_), 7.20 (1H, d, *J*: 3.51 Hz, Ar‐CH), 7.22–7.28 (2H, m, Ar‐CH), 7.49 (1H, d, *J*: 3.57 Hz, thiazole‐CH), 7.55 (2H, d, *J*: 8.67 Hz, Ar‐CH), 7.60 (1H, d, *J*: 7.89 Hz, Ar‐CH), 7.67 (2H, d, *J*: 8.67 Hz, Ar‐CH), 12.45 (1H, s, NH). ^13^C‐NMR (75 MHz, DMSO‐d_6_) δ (ppm): 22.92, 36.12, 114.04, 118.25, 124.62, 129.01, 129.41, 130.09, 131.61, 132.03, 134.52, 135.10, 135.35, 138.24, 141.04, 148.97, 156.12, 158.41, 161.50, 166.71. HRMS (m/z): [M + 1]^+^ calculated 443.0398; found 443.0397.

**2‐((3‐(4‐Chlorophenyl)‐5‐methyl‐4‐oxo‐3,4‐dihydroquinazoline‐2‐yl)thio)‐*N*‐(4,5‐dimethylthiazole‐2‐yl)acetamide (4f)**

C_22_H_19_ClN_4_O_2_S_2_, m.p.: 204°C. White powder. Yield: 60%. IR (cm^−1^): 1682 (C=O stretching band), 1549 and 1470 (C=C stretching band), 866 (1,4‐disubstituted benzene ring out‐of‐plane deformation bands), 804 and 723 (1,3,5‐trisubstituted benzene ring out‐of‐plane deformation bands). ^1^H‐NMR (300 MHz, DMSO‐d_6_) δ (ppm): 2.16 (3H, s, thiazole‐CH_3_), 2.21 (3H, s, thiazole‐CH_3_), 2.68 (3H, s, quinazolinone‐CH_3_), 4.11 (2H, s, S‐CH_2_), 7.22 (1H, d, *J*: 7.34 Hz, quinazolinone‐CH), 7.27–7.33 (2H, m, Ar‐CH), 7.54 (2H, d, *J*: 8.67 Hz, Ar‐CH), 7.66 (2H, d, *J*: 8.62 Hz, Ar‐CH), 12.16 (1H, s, NH). ^13^C‐NMR (75 MHz, D1MSO‐d_6_) δ (ppm): 10.81, 14.76, 22.57, 22.93, 36.20, 124.69, 127.48, 128.10, 129.41, 130.08, 131.63, 132.03, 134.55, 135.35, 141.01, 149.02. HRMS (m/z): [M + 1]^+^ calculated 471.0711; found 471.0722.

**2‐((3‐(4‐Chlorophenyl)‐5‐methyl‐4‐oxo‐3,4‐dihydroquinazoline‐2‐yl)thio)‐*N*‐(4,5‐diphenylthiazole‐2‐yl)acetamide (4g)**

C_32_H_23_ClN_4_O_2_S_2_, m.p.: 237°C. Cream color powder. Yield: 40%. IR (cm^−1^): 3366 (N‐H stretching band), 3169 and 3055 (Aromatic C‐H stretching band), 2986 (Aliphatic C‐H stretching band), 1688 (C=O stretching band), 1597, 1553 and 1489 (C=C stretching band), 829, 804, 748, 692 and 656 (Deformation bands of the benzene ring). ^1^H‐NMR (300 MHz, DMSO‐d_6_) δ (ppm): 2.69 (3H, s, quinazolinone‐CH_3_), 4.20 (2H, s, S‐CH_2_), 7.22 (1H, d, *J*: 7.42 Hz, quinazolinone‐CH), 7.20–7.34 (9H, m, Ar‐CH), 7.43–7.46 (2H, m, Ar‐CH), 7.55–7.61 (2H, m, Ar‐CH), 7.60–7.63 (1H, m, Ar‐CH), 7.66–7.69 (2H, m, Ar‐CH), 12.70 (1H, s, NH). ^13^C‐NMR (75 MHz, DMSO‐d_6_) δ (ppm): 22.93, 36.13, 118.26, 124.73, 125.98, 128.25, 128.46, 128.79, 128.92, 129.04, 129.43, 129.73, 130.10, 132.05, 132.22, 134.55, 135.11, 135.35, 141.05, 144.37, 148.10, 156.07, 156.24, 161.52, 167.17.

**2‐((3‐(4‐Chlorophenyl)‐5‐methyl‐4‐oxo‐3,4‐dihydroquinazoline‐2‐yl)thio)‐*N*‐(6‐methylbenzothiazole‐2‐yl)acetamide (4h)**

C_25_H_19_ClN_4_O_2_S_2_, m.p.: 241°C. White powder. Yield: 56%. IR (cm^−1^): 3061 (Aromatic C‐H stretching band), 2980 and 2932 (Aliphatic C‐H stretching band), 1688 (C=O stretching band), 1547, 1491 and 1468 (C=C stretching band), 802 and 721 (Deformation bands of the benzene ring). ^1^H‐NMR (300 MHz, DMSO‐d_6_) δ (ppm): 2.40 (3H, s, CH_3_), 2.68 (3H, s, CH_3_), 4.20 (2H, s, S‐CH_2_), 7.20 (1H, d, *J*: 7.35 Hz, Ar‐CH), 7.26 (2H, d, *J*: 8.16 Hz, Ar‐CH), 7.56 (1H, d, *J*: 2.16 Hz, Ar‐CH), 7.58 (2H, s, Ar‐CH), 7.65–7.70 (3H, m, Ar‐CH), 7.74 (1H, wide s, Ar‐CH), 12.66 (1H, s, NH). ^13^C‐NMR (75 MHz, DMSO‐d_6_) δ (ppm): 21.43, 22.93, 36.37, 118.25, 120.73, 121.80, 124.59, 127.94, 129.01, 130.10, 132.04, 133.52, 134.54, 135.12, 135.36, 141.05, 147.03, 148.96, 156.11, 157.47, 161.50, 167.72. HRMS (m/z): [M + 1]^+^ calculated 507.0711; found 507.0720.

**2‐((3‐(4‐Chlorophenyl)‐4‐oxo‐3,4‐dihydroquinazoline‐2‐yl)thio)‐N‐(thiazole‐2‐yl)acetamide (4i)**

C_19_H_13_ClN_4_O_2_S_2_, m.p.: 271°C. White powder. Yield: 48%. IR (cm^−1^): 3171 and 3076 (Aromatic C‐H stretching band), 2965 and 2739 (Aliphatic C‐H stretching band), 1672 (C=O stretching band), 1574, 1543, 1489 and 1470 (C=C stretching band), 810, 770 and 739 (Deformation bands of the benzene ring). ^1^H‐NMR (300 MHz, DMSO‐d_6_) δ (ppm): 4.18 (2H, s, S‐CH_2_), 7.21 (1H, d, *J*: 3.54 Hz, thiazole‐CH), 7.43–7.50 (3H, m, Ar‐CH), 7.58 (2H, d, *J*: 8.68 Hz, Ar‐CH), 7.68 (2H, d, *J*: 8.69 Hz, Ar‐CH), 7.79 (1H, td, *J*_*1*_: 1.56 Hz, *J*_*2*_: 7.61 Hz, Ar‐CH), 8.05–8.07 (1H, m, Ar‐CH), 12.47 (1H, s, NH). ^13^C‐NMR (75 MHz, DMSO‐d_6_) δ (ppm): 36.25, 114.06, 119.98, 126.29, 126.60, 127.07, 130.15, 131.94, 135.13, 135.28, 135.45, 138.24, 147.45, 156.73, 158.43, 161.03, 166.67. HRMS (m/z): [M + 1]^+^ calculated 429.0241; found 429.0245.

**2‐((3‐(4‐Chlorophenyl)‐4‐oxo‐3,4‐dihydroquinazoline‐2‐yl)thio)‐*N*‐(4,5‐dimethylthiazole‐2‐yl)acetamide (4j)**

C_21_H_17_ClN_4_O_2_S_2_, m.p.: 188°C. White powder. Yield: 35%. IR (cm^−1^): 3613 (N‐H stretching band), 3055 (aromatic C‐H stretching band), 2968 (aliphatic C‐H stretching band), 1697 and 1688 (C=O stretching band), 1545 and 1466 (C=C stretching band), 808, 770, 733, 691 and 646 (deformation bands of the benzene ring). ^1^H‐NMR (300 MHz, DMSO‐d_6_) δ (ppm): 2.17 (3H, s, thiazole‐CH_3_), 2.21 (3H, s, thiazole‐CH_3_), 4.14 (2H, s, S‐CH_2_), 7.35 (1H, d, *J*: 7.52 Hz, Ar‐CH), 7.46 (2H, wide s, Ar‐CH), 7.58 (2H, d, *J*: 8.72 Hz, Ar‐CH), 7.68 (2H, d, *J*: 7.60 Hz, Ar‐CH), 7.77–7.83 (1H, m, Ar‐CH), 8.06 (1H, d, *J*: 7.56 Hz, Ar‐CH), 12.19 and 13.08 (1H, 2 s, NH). ^13^C‐NMR (75 MHz, DMSO‐d_6_) δ (ppm): 10.81, 14.78, 36.29, 116.20, 116.68, 119.97, 126.36, 126.60, 127.06, 127.88, 129.47, 130.14, 131.53, 131.94, 133.19, 135.14, 135.26, 135.47, 147.47, 156.72, 161.04. HRMS (m/z): [M + 1]^+^ calculated 457.0554; found 457.0558.

**2‐((3‐(4‐Chlorophenyl)‐4‐oxo‐3,4‐dihydroquinazoline‐2‐yl)thio)‐*N*‐(6‐methylbenzothiazole‐2‐yl)acetamide (4k)**

C_24_H_17_ClN_4_O_2_S_2_, m.p.: 227°C. Yellow powder. Yield: 54%. IR (cm^−1^): 3674 (N‐H stretching band), 3065 (Aromatic C‐H stretching band), 2986 and 2901 (Aliphatic C‐H stretching band), 1688 (C=O stretching band), 1574, 1547 and 1476 (C=C stretching band), 810, 764 and 692 (Deformation bands of the benzene ring). ^1^H‐NMR (300 MHz, DMSO‐d_6_) δ (ppm): 2.40 (3H, s, benzothiazole‐CH_3_), 4.21 (2H, s, S‐CH_2_), 7.24 (1H, d, *J*: 8.16 Hz, Ar‐CH), 7.46 (2H, d, *J*: 8.04 Hz, Ar‐CH), 7.57–7.79 (8H, m, Ar‐CH), 8.06 (1H, d, *J*: 7.6 Hz, Ar‐CH), 12.68 (1H, s, NH). ^13^C‐NMR (75 MHz, DMSO‐d_6_) δ (ppm): 21.43, 119.98, 120.58, 121.74, 126.29, 126.58, 127.07, 127.79, 130.16, 131.95, 132.16, 133.26, 135.17, 135.27, 135.47, 147.14, 147.48, 156.88, 161.04, 167.97. HRMS (m/z): [M + 1]^+^ calculated 493.0554; found 493.0567.

**2‐((3‐(4‐Chlorophenyl)‐4‐oxo‐3,4‐dihydroquinazoline‐2‐yl)thio)‐*N*‐(6‐methoxybenzothiazole‐2‐yl)acetamide (4l)**

C_24_H_17_ClN_4_O_3_S_2_, m.p.: 238°C. White powder. Yield: 42%. IR (cm^−1^): 3674 (N‐H stretching band), 2988 and 2901 (Aliphatic C‐H stretching band), 1692 and 1668 (C=O stretching band), 1605, 1545 and 1468 (C=C stretching band), 812 and 766 (Deformation bands of the benzene ring). ^1^H‐NMR (300 MHz, DMSO‐d_6_) δ (ppm): 3.79 (3H, s, OCH_3_), 4.22 (2H, s, S‐CH_2_), 7.04 (1H, dd, *J*_*1*_: 2.61 Hz, *J*_*2*_: 8.87 Hz, benzothiazole‐CH), 7.42–7.47 (2H, m, Ar‐CH), 7.55 (1H, d, *J*: 2.55 Hz, Ar‐CH), 7.60 (2H, d, *J*: 8.71 Hz, Ar‐CH), 7.67 (2H, d, *J*: 5.7 Hz, Ar‐CH), 7.70 (1H, d, *J*: 5.67 Hz, Ar‐CH), 7.75–7.81 (1H, m, Ar‐CH), 8.06 (1H, dd, *J*_*1*_: 1.26 Hz, *J*_*2*_: 8.18 Hz, Ar‐CH), 12.60 (1H, s, NH). ^13^C‐NMR (75 MHz, DMSO‐d_6_) δ (ppm): 36.47, 56.09, 105.18, 115.46, 119.98, 121.70, 126.29, 126.61, 127.09, 130.16, 131.95, 133.23, 135.14, 135.29, 135.49, 143.13, 147.44, 156.32, 156.63, 161.02, 167.50. HRMS (m/z): [M + 1]^+^ calculated 509.0503; found 509.0516.

***N*‐(6‐Chlorobenzothiazole‐2‐yl)‐2‐((3‐(4‐chlorophenyl)‐4‐oxo‐3,4‐dihydroquinazoline‐2‐yl)thio)acetamide (4m)**

C_23_H_14_Cl_2_N_4_O_2_S_2_, m.p.: 238°C. Light orange powder. Yield: 42%. IR (cm^−1^): 3071 (Aromatic C‐H stretching band), 2988 and 2930 (Aliphatic C‐H stretching band), 1707 and 1688 (C=O stretching band), 1609, 1547, 1489 and 1476 (C=C stretching band), 822, 766 and 694 (Deformation bands of the benzene ring). ^1^H‐NMR (300 MHz, DMSO‐d_6_) δ (ppm): 4.24 (2H, s, S‐CH_2_), 7.43–7.48 (3H, m, Ar‐CH), 7.60 (2H, d, *J*: 7.20 Hz, Ar‐CH), 7.70 (2H, d, *J*: 7.20 Hz, Ar‐CH), 7.78 (2H, d, *J*: 5.08 Hz, Ar‐CH), 8.05–8.10 (2H, m, Ar‐CH), 12.88 (1H, wide s, NH). ^13^C‐NMR (75 MHz, DMSO‐d_6_) δ (ppm): 36.62, 119.98, 121.91, 122.24, 126.25, 126.62, 126.93, 127.09, 128.03, 130.17, 131.95, 133.65, 135.14, 135.30, 135.49, 147.43, 147.99, 156.74, 161.02, 168.23.

**2‐((3‐Benzyl‐4‐oxo‐3,4‐dihydroquinazoline‐2‐yl)thio)‐*N*‐(thiazole‐2‐yl)acetamide (4n)**

C_20_H_16_N_4_O_2_S_2_, m.p.: 235°C. White powder. Yield: 48%. IR (cm^−1^): 3196 and 3132 (Aromatic C‐H stretching band), 2936 and 2731 (Aliphatic C‐H stretching band), 1682 (C=O stretching band), 1587, 1537 and 1470 (C=C stretching band), 858, 768, 729, 706 and 625 (Deformation bands of the benzene ring). ^1^H‐NMR (300 MHz, DMSO‐d_6_) δ (ppm): 4.28 (2H, s, S‐CH_2_), 5.36 (2H, s, N‐CH_2_), 7.21 (1H, wide s, Ar‐CH), 7.30–7.39 (6H, m, Ar‐CH), 7.46 (1H, t, *J*_*1*_: 7.56 Hz, *J*_*2*_: 7.52 Hz, Ar‐CH), 7.51 (1H, wide s, Ar‐CH), 7.77 (1H, t, *J*_*1*_: 7.48 Hz, *J*_*2*_: 7.58 Hz, Ar‐CH), 8.10 (1H, d, *J*: 7.68 Hz, Ar‐CH), 12.53 (1H, s, NH). ^13^C‐NMR (75 MHz, DMSO‐d_6_) δ (ppm): 36.08, 47.54, 114.05, 119.16, 126.17, 126.68, 127.11, 127.38, 128.10, 129.10, 135.41, 136.01, 138.26, 147.09, 156.74, 158.46, 161.27, 166.64. HRMS (m/z): [M + 1]^+^ calculated 409.0787; found 409.0777.

**2‐((3‐Benzyl‐4‐oxo‐3,4‐dihydroquinazoline‐2‐yl)thio)‐*N*‐(4‐fluorophenyl)acetamide (4o)**

C_23_H_18_FN_3_O_2_S, m.p.: 209°C. Light brown powder. Yield: 60%. IR (cm^−1^): 3279, 3152 and 3073 (Aromatic C‐H stretching band), 2972 (Aliphatic C‐H stretching band), 1661 (C=O stretching band), 1607, 1545, 1510 and 1470 (C=C stretching band), 831, 770, 716 and 692 (Deformation bands of the benzene ring). ^1^H‐NMR (300 MHz, DMSO‐d_6_) δ (ppm): 4.19 (2H, s, S‐CH_2_), 5.37 (2H, s, N‐CH_2_), 7.16 (2H, t, *J*_*1*_: 8.52 Hz, *J*_*2*_: 8.06 Hz, Ar‐CH), 7.30–7.38 (5H, m, Ar‐CH), 7.45–7.51 (2H, m, Ar‐CH), 7.61–7.63 (2H, m, Ar‐CH), 7.80 (1H, t, *J*_*1*_: 7.36 Hz, *J*_*2*_: 7.62 Hz, Ar‐CH), 8.11 (1H, d, *J*: 7.87 Hz, Ar‐CH), 10.47 (1H, s, NH). ^13^C‐NMR (75 MHz, DMSO‐d_6_) δ (ppm): 37.39, 47.48, 115.74, 115.96, 119.18, 121.35, 121.42, 126.29, 126.63, 127.13, 127.34, 127.95, 129.08, 135.42, 135.84, 136.05, 147.21, 157.04, 157.35, 159.73, 161.29, 165.99. HRMS (m/z): [M + 1]^+^ calculated 420.1177; found 420.1177.

**2‐((3‐Benzyl‐6‐chloro‐4‐oxo‐3,4‐dihydroquinazoline‐2‐yl)thio)‐*N*‐(4‐fluorophenyl)acetamide (4p)**

C_23_H_17_ClFN_3_O_2_S, m.p.: 197°C. White powder. Yield: 62%. IR (cm^−1^): 3252 and 3065 (Aromatic C‐H stretching band), 2982 and 2928 (Aliphatic C‐H stretching band), 1672 (C=O stretching band), 1607, 1543, and 1468 (C=C stretching band), 829, 804, 721, and 694 (Deformation bands of the benzene ring). ^1^H‐NMR (300 MHz, DMSO‐d_6_) δ (ppm): 4.18 (2H, s, S‐CH_2_), 5.36 (2H, s, N‐CH_2_), 7.16 (2H, t, *J*_*1*_: 8.06 Hz, *J*_*2*_: 8.14 Hz, Ar‐CH), 7.342–7.36 (5H, m, Ar‐CH), 7.51 (1H, d, *J*: 8.72 Hz, Ar‐CH), 7.61–7.62 (2H, m, Ar‐CH), 7.84 (1H, d, *J*: 8.72 Hz, Ar‐CH), 8.04 (1H, s, quinazolinone‐CH), 10.46 (1H, s, NH). ^13^C‐NMR (75 MHz, DMSO‐d_6_) δ (ppm): 37.43, 47.70, 115.75, 115.97, 120.43, 121.37, 121.45, 126.07, 127.37, 127.71, 128.02, 128.51, 128.71, 129.09, 130.62, 135.54, 135.76, 145.93, 157.36, 157.87, 160.38, 165.88. HRMS (m/z): [M + 1]^+^ calculated 454.0787; found 454.0781.

**2‐((3‐(4‐Fluorophenyl)‐7‐methyl‐4‐oxo‐3,4‐dihydroquinazoline‐2‐yl)thio)‐*N*‐(4‐methoxyphenyl)acetamide (4r)**

C_24_H_20_FN_3_O_3_S, m.p.: 257°C. White powder. Yield: 60%. IR (cm^−1^): 3281–3009 (Aromatic C‐H stretching band), 2928 and 2833 (Aliphatic C‐H stretching band), 1678 and 1651 (C=O stretching band), 1537 and 1512 (C=C stretching band), 800 and 781 (Deformation bands of the benzene ring). ^1^H‐NMR (300 MHz, DMSO‐d_6_) δ (ppm): 2.45 (3H, s, Quinazolinone‐CH_3_), 3.71 (3H, s, ‐OCH_3_), 4.06 (2H, s, S‐CH_2_), 6.88 (2H, d, *J*: 7.72 Hz, Ar‐CH), 7.30 (1H, d, *J*: 7.72 Hz, Ar‐CH), 7.38 (1H, s, Ar‐CH), 7.43 (2H, t, *J*_*1*_: 7.48 Hz, *J*_*2*_: 7.66 Hz, Ar‐CH), 7.49 (2H, d, *J*: 7.68 Hz, Ar‐CH), 7.57 (2H, wide s, Ar‐CH), 7.96 (1H, d, *J*: 7.80 Hz, Ar‐CH), 10.18 (1H, s, NH). ^13^C‐NMR (75 MHz, DMSO‐d_6_) δ (ppm): 21.90, 37.65, 55.62, 114.36, 116.83, 117.06, 117.59, 121.07, 121.17, 126.10, 126.97, 127.91, 132.32, 132.41, 132.54, 145.97, 147.68, 155.81, 157.35, 161.07, 161.85, 164.30, 165.53.

### 3.2 Experimental Activity Studies

#### 3.2.1 In Vitro Cytotoxicity Evaluation Test (MTT Test)

Cytotoxicity of final molecules investigated against three different cell lines. MTT test was conducted on A549 (human alveolar epithelial cells), MCF‐7 (lung cancer cells), and L929 (mouse fibroblast cells) cell lines. Multiplication and preparation of the cells were carried out via passaging regularly every 2 days. Cell culture obtained from incubation medium was shaken slowly to prevent the dead cells from transferring to the culture medium. After that, culture medium solutions were removed. 5 mL phosphate buffer was added to the culture bottle to wash the cells and solutions removed from the medium. 1X Trypsin–EDTA solution was added to the culture medium and then incubated approximately 5 min (%95 humidity, 37°C, %5 CO_2_). Approximately 25 mL of culture medium was added to the bottle taken from the incubation medium, cells were suspended and divided at a ratio of 1:2 or 1:3, and transferred to clean culture bottles that did not contain any substance or culture medium. Culture bottles were placed in the incubator to grow cells. Medium was added to the cell suspension and transferred to a centrifuge tube.

After centrifuging cell suspension, 10 μL of suspension was taken and stained with 10 μL of trypan blue. Cell suspension was distributed into a cell culture plate at concentration of 2 × 10^4^ cells/well and incubated for 1 day. Reference drug cisplatin was administered at eight different dilutions from 3.9 to 500 μg/mL to compare the antiproliferative efficacy of resulting molecules. When growth rate of the cells reached 80%, cells separated via %0.25 trypsin–EDTA solutions and counted according to the hemocytometry method (Asaf E. Evren et al. 2019; Evren et al. 2021; Yurttas et al. 2018).

#### 3.2.2 Apoptosis Induction (Flow Cytometric Method)

Annexin‐V is a type of protein that forms a bond with phosphatidylserine phospholipid. Annexin‐V was marked with a fluorescent substance (FITC, fluorescein isothiocyanate) to make the apoptotic cells visible.

First, based on the cytotoxicity test results, most active molecules chosen, three different concentrations of the compounds implemented to cancerous cell lines. After applications, supernatants divided to sterile tubes. Precipitate at the bottom of the tubes washed with %0.25 concentration trypsin for 5 min and separated. Tubes centrifuged at 4°C, 1200 rpm for 5 min. Cell counting is carried out with trypan blue staining method. After that, tubes were cooled down and centrifuged at 4°C, 1200 rpm for 5 min. After supernatant was separated and discarded, the number of cells remaining at the bottom was adjusted to 1 × 10^6^ cells using “Binding buffer” according to the procedure specified in the kit. 5 μL of Annexin‐V FITC and 10 μL of PI were mixed in the tube. Cell suspension was mixed with 100 μL of PI and incubated in a dark place at 20°C–25°C for 15 min. After this time, 400 μL of binding buffer was added to resuspend cells. Measurements were performed using a flow cytometry device (Evren et al. 2019; Yurttas et al. 2015).

#### 3.2.3 Evaluation of Caspase‐3 Activation

Caspase‐3 activation study performed with spectrofluorometric caspase‐3 assay kit (BD Pharmingen, NJ). Firstly, cells (1–10^6^ cell/mL) were washed with phosphate buffered saline (PBS), re‐suspended in lysis buffer and incubated on ice for half an hour. After a day incubation of compounds and mitoxantrone at different concentrations, cell lysates were prepared. For each reaction, 5 mL diluted Ac‐DEVD‐AMC was added to 0.2 mL 1 HEPES buffer containing wells. Cell lysate (20 mL) was added to the wells. The reaction mixtures were incubated at 37°C for an hour. The quantity of AMC emitted from Ac‐DEVD‐AMC was measured using a microplate reader (Perkin Elmer Victor) with an excitation wavelength of 380 nm and an emission wavelength of 460 nm. All experiments were performed twice. Double‐well setups were used at all dose levels (Ciftci et al. 2015; Yurttas et al. 2017; Yurttas et al. 2014).

#### 3.2.4 EGFR Inhibition Study

EGFR is a member of the tyrosine kinase family. Over expression or hyperactivation of the EGFR enzyme found related to various cancer types. To investigate the EGFR kinase enzyme inhibition of the compounds, the EGFR kinase enzyme assay kit (BPS Bioscience, USD) was chosen. The test procedure was conducted in accordance with instructions provided by the manufacturer. Gefitinib was chosen as the standard drug (Abourehab et al. 2021; Dokala and Thakur 2017; Moasser 2007; Westphal et al. 2017).

### 3.3 In Silico Analysis

#### 3.3.1 Molecular Docking Studies

Molecular docking analysis was carried out to observe interactions between the enzyme and final compounds. Based on the results of the anticancer activity studies, compound **4j** was chosen to be docked. Crystal structures of caspase‐3 and EGFR enzymes derived from Protein Data Bank (PDB ID: 4QTX, PDB ID: 2ITY, respectively) (Schrödinger 2020b). Crystal structures of the enzymes were prepared separately by using Schrödinger Maestro software. Protein preparation step was carried out with Schrödinger Suite 2020 program. Compound **4j** was prepared with LigPrep module at 7.4 ± 1.0 pH (Schrödinger 2020c). Mapping of the enzyme‐compound interaction site was completed using the Glide module (Schrödinger 2020a). Ligand docking was performed utilizing the standard precision (SP) method.

#### 3.3.2 Molecular Dynamics Simulations

In correlation with molecular docking results, molecular dynamics simulations conducted on compound **4j** to investigate the state of the interactions. Protein structure and **4j** were prepared separately by using Desmond's ‘System Setup’ module. In simulation studies, the transferable three‐point intermolecular potential water model was used as a hydration model. Palmitoyloleoylphosphatidylcholine (POPC) membrane model was chosen while preparing the simulation (Abd Halim et al. 2015; Kalli et al. 2015). Neutralization of the system provided by Na^+^ and Cl^−^ ions. To observe the stability of the interactions between enzyme and **4j**, 100 ns long simulations was carried out (Dawbaa et al. 2022; Evren et al. 2023, 2022; Osmaniye et al. 2022; Turan Yucel et al. 2022).

## 4 Conclusion

In the present study, novel 17 quinazolin‐4‐(3*H*)‐one derivatives were synthesized, crystallized and structural analysis performed by various spectral techniques (IR, HRMS, ^1^H‐NMR, and ^13^C‐NMR). Anticancer activity profiles of final molecules were investigated. MTT tests were performed on cancerous cell lines A549, MCF‐7, and L929. Based on the activity test results, the most active compound was chosen and in silico studies were conducted.

Cytotoxicity test results indicated that compound **4j** exhibited higher inhibitory activity than cisplatin and gefitinib on A549 cell line. However, **4j** showed lower inhibitory activity on MCF‐7 cells. **4a** exhibited comparable inhibition with reference drugs on MCF‐7. Among the resulted compounds, **4d**, **4h**, **4k**, **4m**, and **4n** showed selective inhibition on A549 cell line and did not exhibit toxic effects on noncancerous cell line L929. Based on the findings of cytotoxicity tests, apoptosis induction activity studies were performed on A549 and L929 cells. Compounds **4d**, **4a**, **4b**, and **4f** exhibited two times higher apoptotic effect than cisplatin on A549. Although, on MCF‐7 cells **4j** was found to show comparable apoptotic effect with cisplatin. Caspase‐3 enzyme inhibition studies were conducted to determine the pathway of the apoptosis induction. Experiments conducted on two different cell lines showed that in the A549 cell line, compounds **4o**, **4a**, and **4m** and in the MCF‐7 cell line, compounds **4a** and **4o** inhibited the caspase‐3 enzyme to a greater extent than cisplatin, while exhibiting a similar level of inhibition to gefitinib. Based on the EGFR inhibition study, **4b**, **4f**, and **4j** showed higher inhibition than gefitinib. In silico molecular docking and molecular dynamics simulations were performed in the light of anticancer activity tests results. Molecular docking studies were carried out on both caspase‐3 and EGFR enzyme. It has been observed that **4j** interacts with the essential amino acids Arg207, Trp206, and Cys163 in caspase‐3 activation by forming hydrogen and π‐π bonds. In the docking study with the EGFR enzyme, it was determined that the compound plays a role in enzyme inhibition by forming a hydrogen bond with the Met793 amino acid located at the enzyme's hinge structure. Molecular dynamics simulations have shown that **4j** inhibits EGFR enzyme by forming various hydrogen bonds.

Within the scope of this study, the quinazoline‐4(3*H*)‐one ring, known for its anticancer activity and particularly its EGFR inhibitor activity, has been modified with various structures. Aim of this study is to present the results to the literature and to provide a new perspective on the development of anticancer drug candidates.

## Author Contributions

**Leyla Yurttaş:** conceptualization, methodology, validation, formal analysis, investigation, resources, data curation, writing – original draft, writing – review and editing, visualization, supervision, project administration, funding acquisition. **Asaf Evrim Evren:** conceptualization, methodology, software, validation, formal analysis, investigation, data curation, writing – original draft, writing – review and editing, visualization, supervision. **Aybüke Züleyha Kaya:** conceptualization, methodology, software, validation, formal analysis, investigation, data curation, writing – original draft, writing – review and editing, visualization. **Gülşen Akalın‐Çiftçi:** methodology, validation, formal analysis, investigation, data curation.

## Funding

This master's thesis was financially funded by Anadolu University Scientific Research Projects Coordination Unit (BAP) grant number “TYL‐2025‐2850”.

## Disclosure

This study is derived from Aybüke Züleyha KAYA's master's thesis entitled “Synthesis Of Quinazolinone Derivatives And Investigation Of Their Anticancer Activities” completed in 2025 at the Department of Pharmaceutical Chemistry, Institute of Graduate Education, Anadolu University.

## Ethics Statement

No animal or clinical experiments requiring ethical approval have been conducted.

## Conflicts of Interest

The authors declare no conflicts of interest.

## Supporting information


**Figure S1:** IR spectrum of compound **4a**.
**Figure S2:** HRMS spectrum of compound **4a**.
**Figure S3:** ^1^H‐NMR spectrum of compound **4a**.
**Figure S4:** ^13^C‐NMR spectrum of compound **4a**.
**Figure S5:** IR spectrum of compound **4b**.
**Figure S6:** HRMS spectrum of compound **4b**.
**Figure S7:** ^1^H‐NMR spectrum of compound **4b**.
**Figure S8:** ^13^C‐NMR spectrum of compound **4b**.
**Figure S9:** IR spectrum of compound **4c**.
**Figure S10:** ^1^H‐NMR spectrum of compound **4c**.
**Figure S11:** ^13^C‐NMR spectrum of compound **4c**.
**Figure S12:** IR spectrum of compound **4d**.
**Figure S13:** ^1^H‐NMR spectrum of compound **4d**.
**Figure S14:** ^13^C‐NMR spectrum of compound **4d**.
**Figure S15:** IR spectrum of compound **4e**.
**Figure S16:** HRMS spectrum of compound **4e**.
**Figure S17:** ^1^H‐NMR spectrum of compound **4e**.
**Figure S18:** ^13^C‐NMR spectrum of compound **4e**.
**Figure S19:** IR spectrum of compound **4f**.
**Figure S20:** HRMS spectrum of **4f**.
**Figure S21:** ^1^H‐NMR spectrum of compound **4f**.
**Figure S22:** ^13^C‐NMR spectrum of compound **4f**.
**Figure S23:** IR spectrum of compound **4g**.
**Figure S24:** ^1^H‐NMR spectrum of compound **4g**.
**Figure S25:** ^13^C‐NMR spectrum of compound **4g**.
**Figure S26:** IR spectrum of compound **4h**.
**Figure S27:** HRMS spectrum of compound **4h**.
**Figure S28:** ^1^H‐NMR spectrum of compound **4h**.
**Figure S29:** ^13^C‐NMR spectrum of compound **4h**.
**Figure S30:** IR spectrum of compound **4i**.
**Figure S31:** HRMS spectrum of compound **4i**.
**Figure S32:** ^1^H‐NMR spectrum of compound **4i**.
**Figure S33:** ^13^C‐NMR spectrum of compound **4i**.
**Figure S34:** IR spectrum of compound **4j**.
**Figure S35:** HRMS spectrum of compound **4j**.
**Figure S36:** ^1^H‐NMR spectrum of compound **4j**.
**Figure S37:** ^13^C‐NMR spectrum of compound **4j**.
**Figure S38:** IR spectrum of compound **4k**.
**Figure S39:** HRMS spectrum of compound **4k**.
**Figure S40:** ^1^H‐NMR spectrum of compound **4k**.
**Figure S41:** ^13^C‐NMR spectrum of compound **4k**.
**Figure S42:** IR spectrum of compound **4l**.
**Figure S43:** HRMS spectrum of compound **4l**.
**Figure S44:** ^1^H‐NMR spectrum of compound **4l**.
**Figure S45:** ^13^C‐NMR spectrum of compound **4l**.
**Figure S46:** IR spectrum of compound **4m**.
**Figure S47:** ^1^H‐NMR spectrum of compound **4m**.
**Figure S48:** ^13^C‐NMR spectrum of compound **4m**.
**Figure S49:** IR spectrum of compound **4n**.
**Figure S50:** HRMS spectrum of compound **4n**.
**Figure S51:** ^1^H‐NMR spectrum of compound **4n**.
**Figure S52:** ^13^C‐NMR spectrum of compound **4n**.
**Figure S53:** IR spectrum of compound **4o**.
**Figure S54:** HRMS spectrum of compound **4o**.
**Figure S55:** ^1^H‐NMR spectrum of compound **4o**.
**Figure S56:** ^13^C‐NMR spectrum of compound **4o**.
**Figure S57:** IR spectrum of compound **4p**.
**Figure S58:** HRMS spectrum of compound **4p**.
**Figure S59:** ^1^H‐NMR spectrum of compound **4p**.
**Figure S60:** ^13^C‐NMR spectrum of compound **4p**.
**Figure S61:** IR spectrum of compound **4r**.
**Figure S62:** ^1^H‐NMR spectrum of compound **4r**.
**Figure S63:** ^13^C‐NMR spectrum of compound **4r**.

## Data Availability

Data sharing not applicable to this article as no datasets were generated or analysed during the current study.
