# Supplementary material for: Design, Synthesis of Novel Quinazolinone Derivatives and Evaluation of EGFR Kinase Inhibition Activity via In Vitro and In Silico Studies
Source: Chem Biol Drug Des. 2026 Jun 19;107(6):e70338. doi: 10.1111/cbdd.70338 (PMC13280546; doi:10.1111/cbdd.70338)
Supplement: Supplementary file 1 — Figure S1: IR spectrum of compound 4a. Figure S2: HRMS spectrum of compound 4a. Figure S3: 1H‐NMR spectrum of compound 4a. Figure S4: 13C‐NMR spectrum of compound 4a. Figure S5: IR spectrum of compound 4b. Figure S6: HRMS spectrum of compound 4b. Figure S7: 1H‐NMR spectrum of compound 4b. Figure S8: 13C‐NMR spectrum of compound 4b. Figure S9: IR spectrum of compound 4c. Figure S10: 1H‐NMR spectrum of compound 4c. Figure S11: 13C‐NMR spectrum of compound 4c. Figure S12: IR spectrum of compound 4d. Figure S13: 1H‐NMR spectrum of compound 4d. Figure S14: 13C‐NMR spectrum of compound 4d. Figure S15: IR spectrum of compound 4e. Figure S16: HRMS spectrum of compound 4e. Figure S17: 1H‐NMR spectrum of compound 4e. Figure S18: 13C‐NMR spectrum of compound 4e. Figure S19: IR spectrum of compound 4f. Figure S20: HRMS spectrum of 4f. Figure S21: 1H‐NMR spectrum of compound 4f. Figure S22: 13C‐NMR spectrum of compound 4f. Figure S23: IR spectrum of compound 4g. Figure S24: 1H‐NMR spectrum of compound 4g. Figure S25: 13C‐NMR spectrum of compound 4g. Figure S26: IR spectrum of compound 4h. Figure S27: HRMS spectrum of compound 4h. Figure S28: 1H‐NMR spectrum of compound 4h. Figure S29: 13C‐NMR spectrum of compound 4h. Figure S30: IR spectrum of compound 4i. Figure S31: HRMS spectrum of compound 4i. Figure S32: 1H‐NMR spectrum of compound 4i. Figure S33: 13C‐NMR spectrum of compound 4i. Figure S34: IR spectrum of compound 4j. Figure S35: HRMS spectrum of compound 4j. Figure S36: 1H‐NMR spectrum of compound 4j. Figure S37: 13C‐NMR spectrum of compound 4j. Figure S38: IR spectrum of compound 4k. Figure S39: HRMS spectrum of compound 4k. Figure S40: 1H‐NMR spectrum of compound 4k. Figure S41: 13C‐NMR spectrum of compound 4k. Figure S42: IR spectrum of compound 4l. Figure S43: HRMS spectrum of compound 4l. Figure S44: 1H‐NMR spectrum of compound 4l. Figure S45: 13C‐NMR spectrum of compound 4l. Figure S46: IR spectrum of compound 4m. Figure S47: 1H‐NMR spectrum of compound 4m. Figure S [file CBDD-107-e70338-s001.docx]

**SUPPLEMENTARY DATA**

**Design, synthesis of novel quinazolinone derivatives and evaluation of anticancer activity targeting EGFR inhibition via *in vitro* and *in silico* studies**

Aybüke Züleyha KAYA^1,2,*^, Asaf Evrim EVREN^1^, Gülşen Akalın-Çiftçi^3^, Leyla YURTTAŞ^1^

*^1^ Anadolu University, Faculty of Pharmacy, Department of Pharmaceutical Chemistry, Eskisehir, Turkey.*

*^2^ Institute of Graduate Education, Anadolu University, Eskisehir, Turkey.*

*^3^ Anadolu University, Faculty of Pharmacy, Department of Biochemistry, Eskisehir, Turkey.*

**Corresponding Author:* [*aybukezuleyhakaya@anadolu.edu.tr*](mailto:aybukezuleyhakaya@anadolu.edu.tr)

***2-((3-(4-Chlorophenyl)-6-methyl-4-oxo-3,4-dihydroquinazoline-2-yl)thio)-N-(thiazole-2-yl)acetamide (4a)***


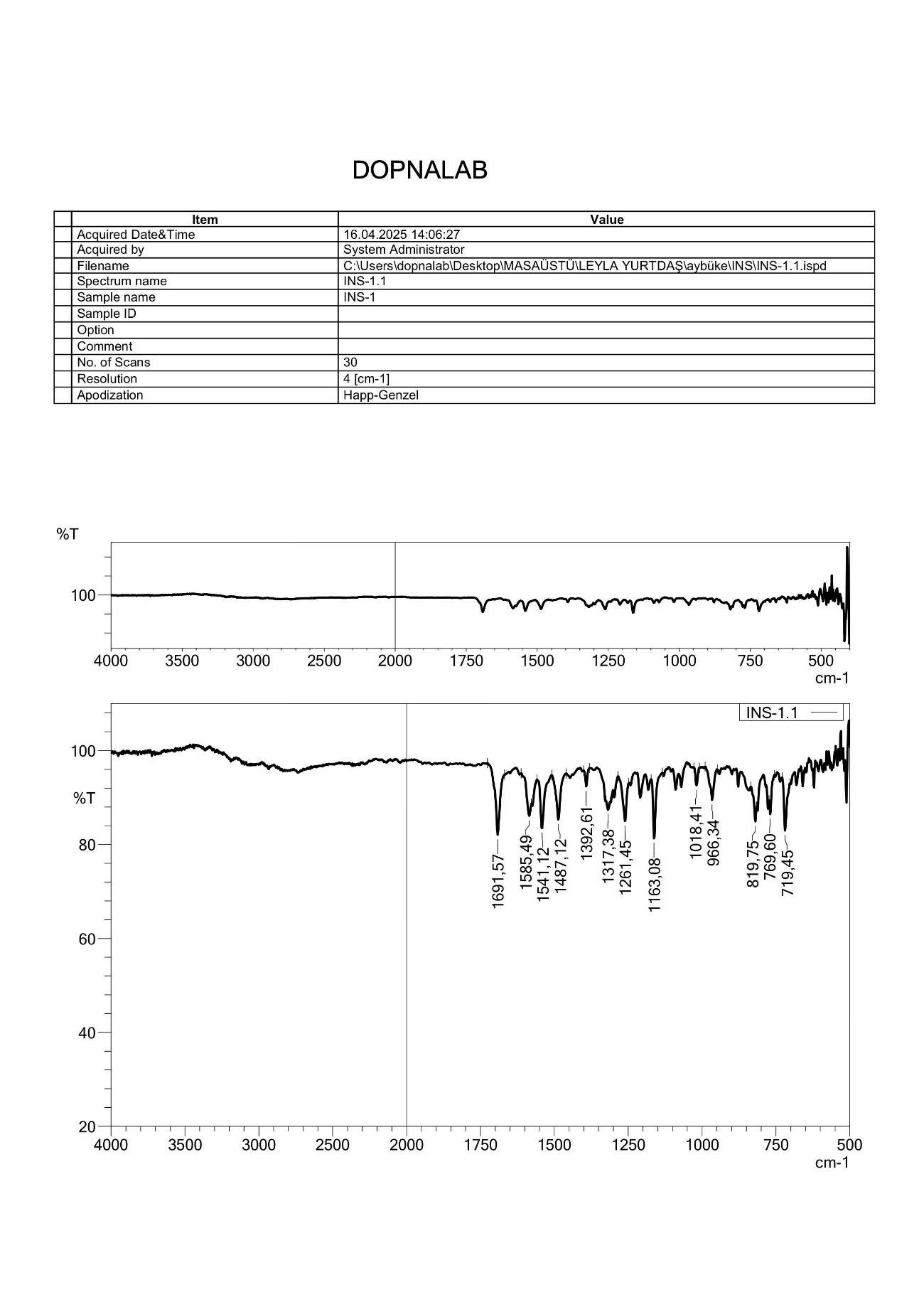


Figure S1. IR spectrum of compound 4a

*
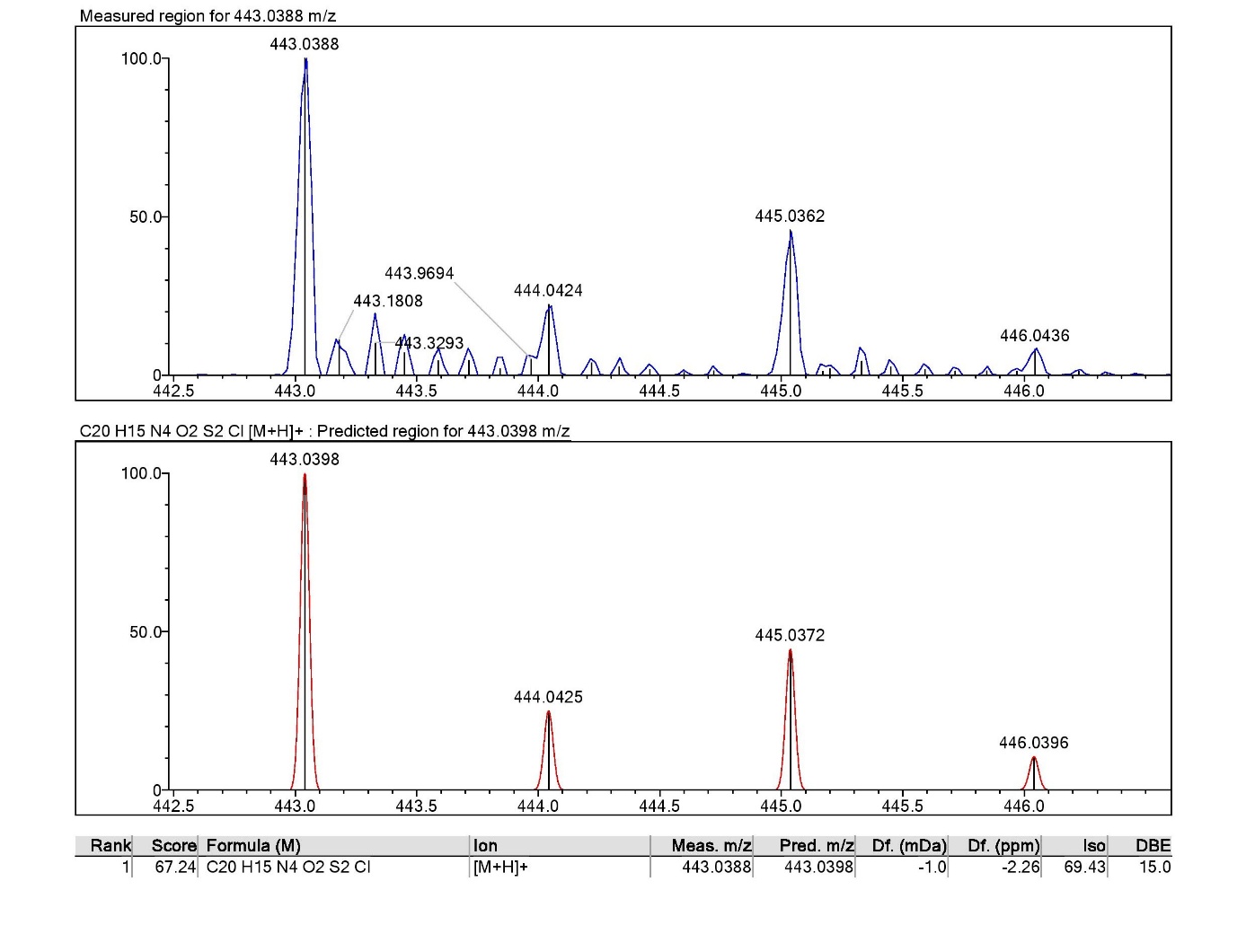
*

**Figure S2**. HRMS spectrum of compound **4a**


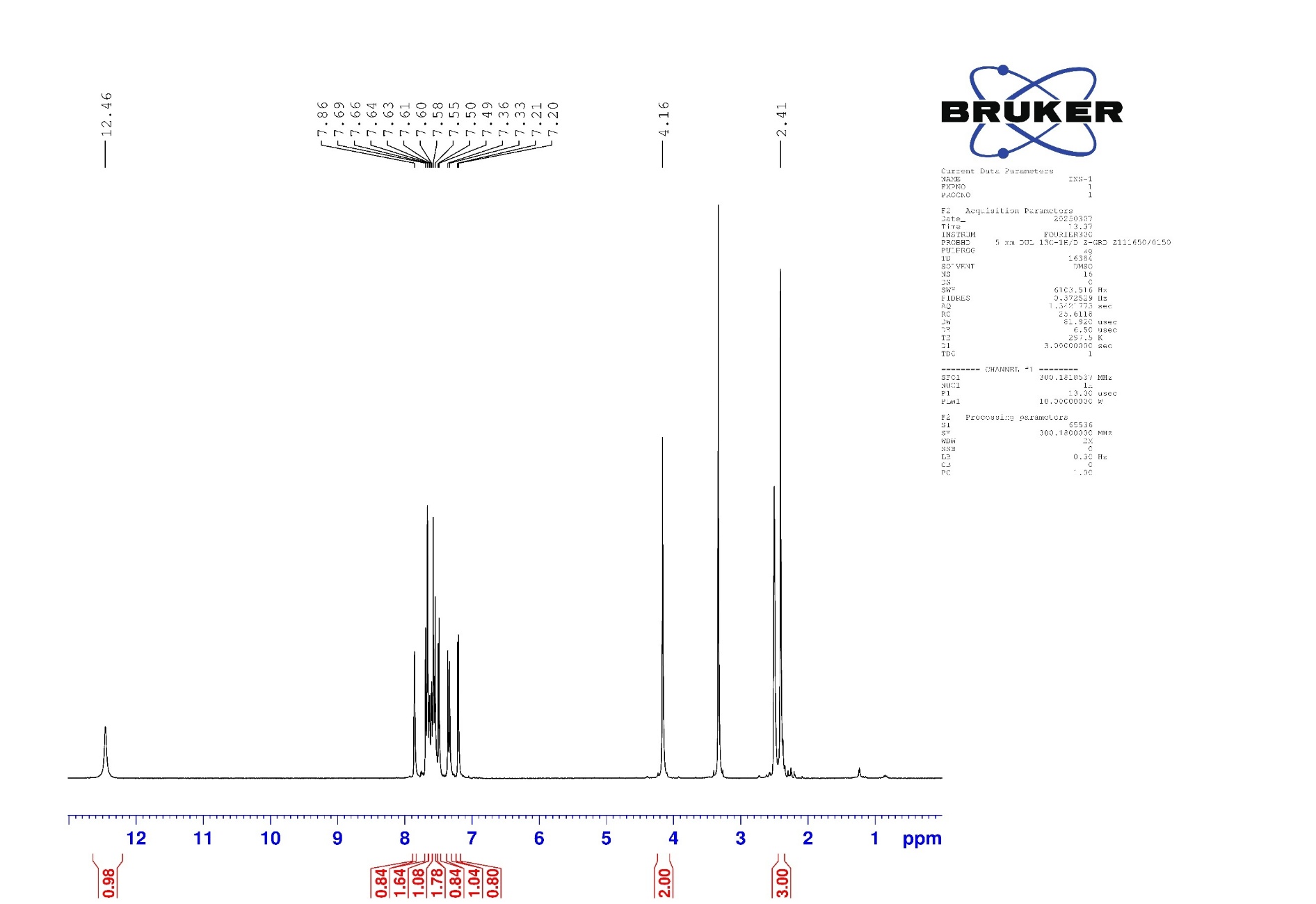


Figure S3. ^1^H-NMR spectrum of compound 4a


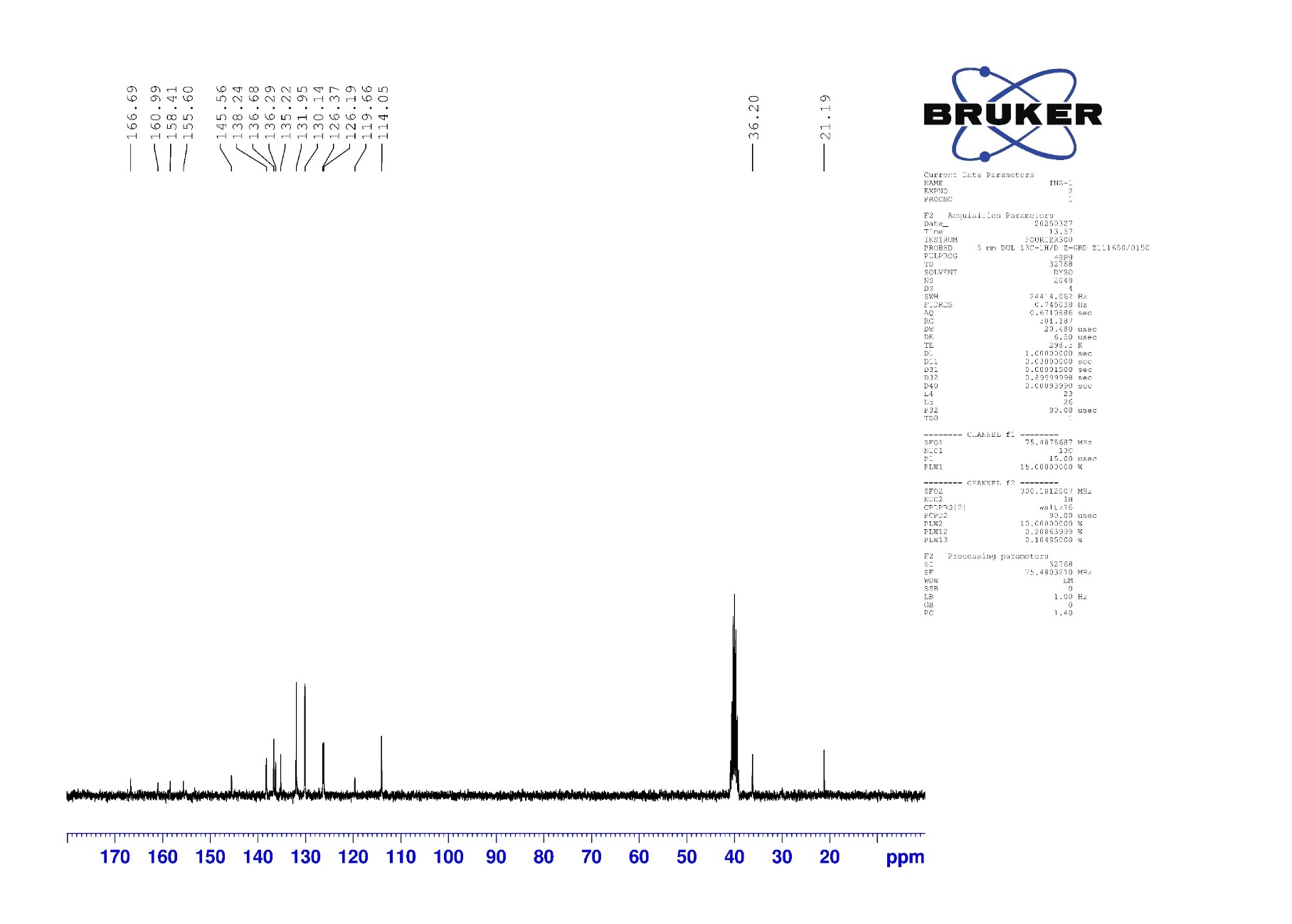


Figure S4. ^13^C-NMR spectrum of compound 4a

***2-((3-(4-Chlorophenyl)-6-methyl-4-oxo-3,4-dihydroquinazoline-2-yl)thio)-N-(4-methylthiazole-2-yl)acetamide (4b)***


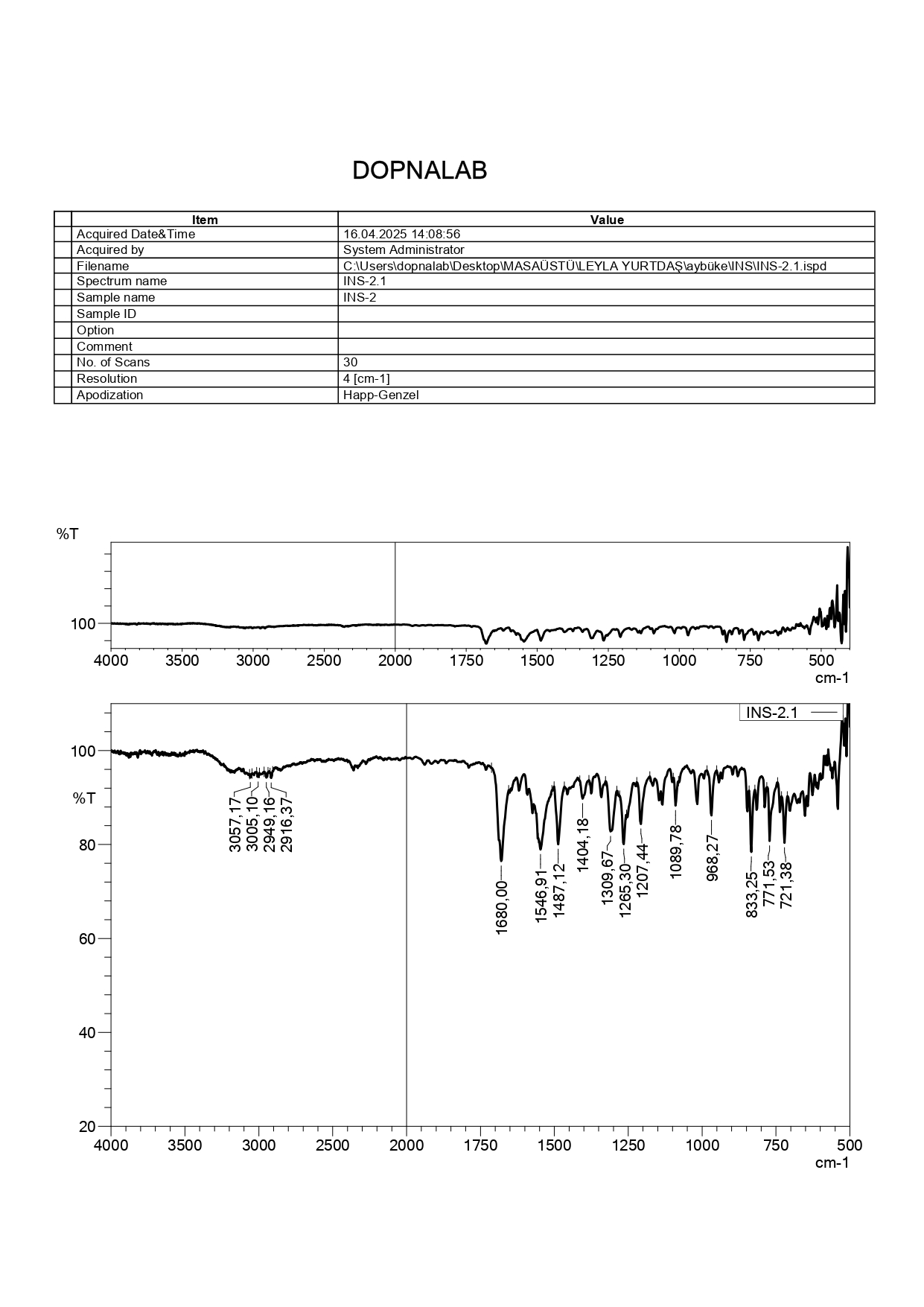


**Figure S5.** IR spectrum of compound **4b**

**
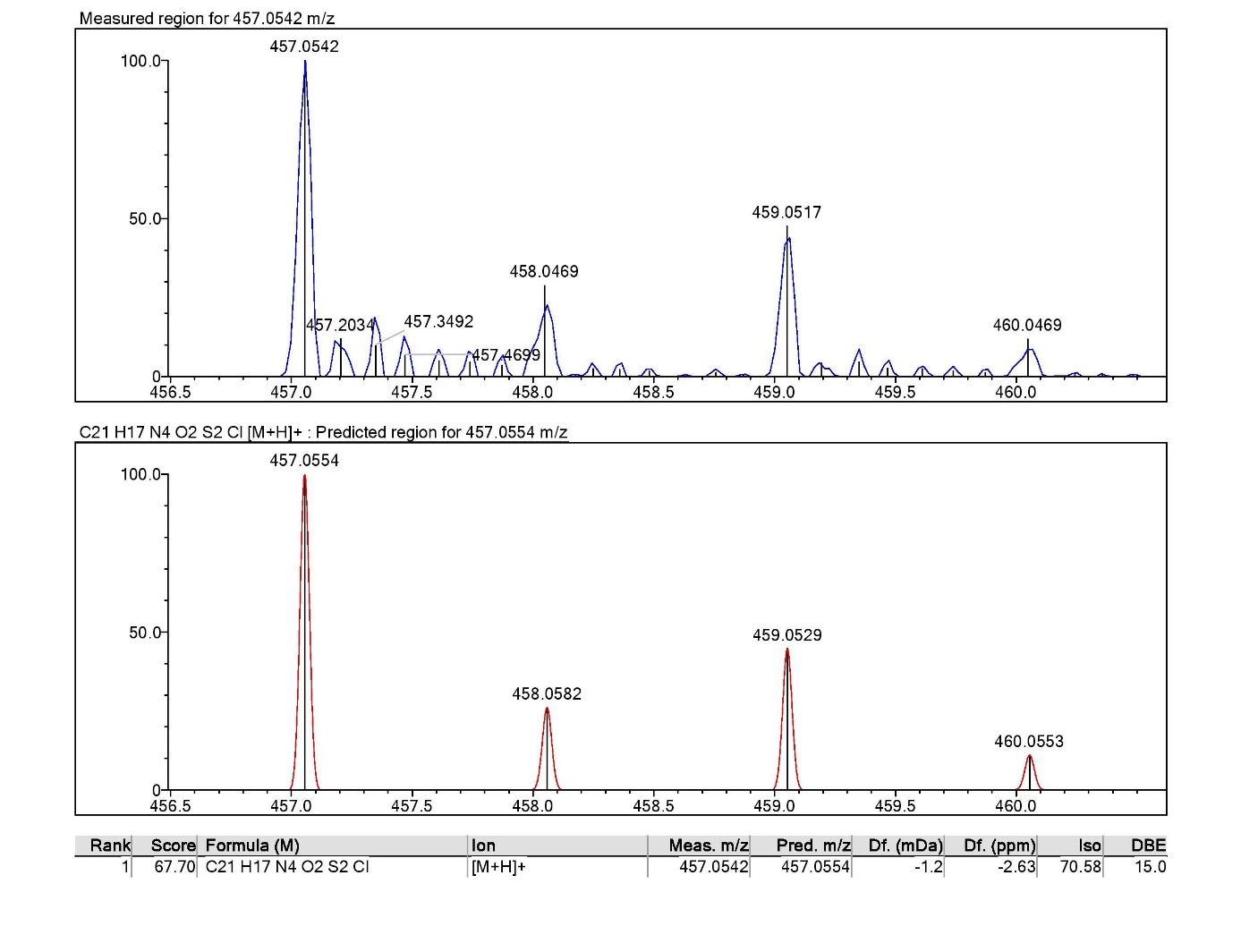
**

**Figure S6.** HRMS spectrum of compound **4b**


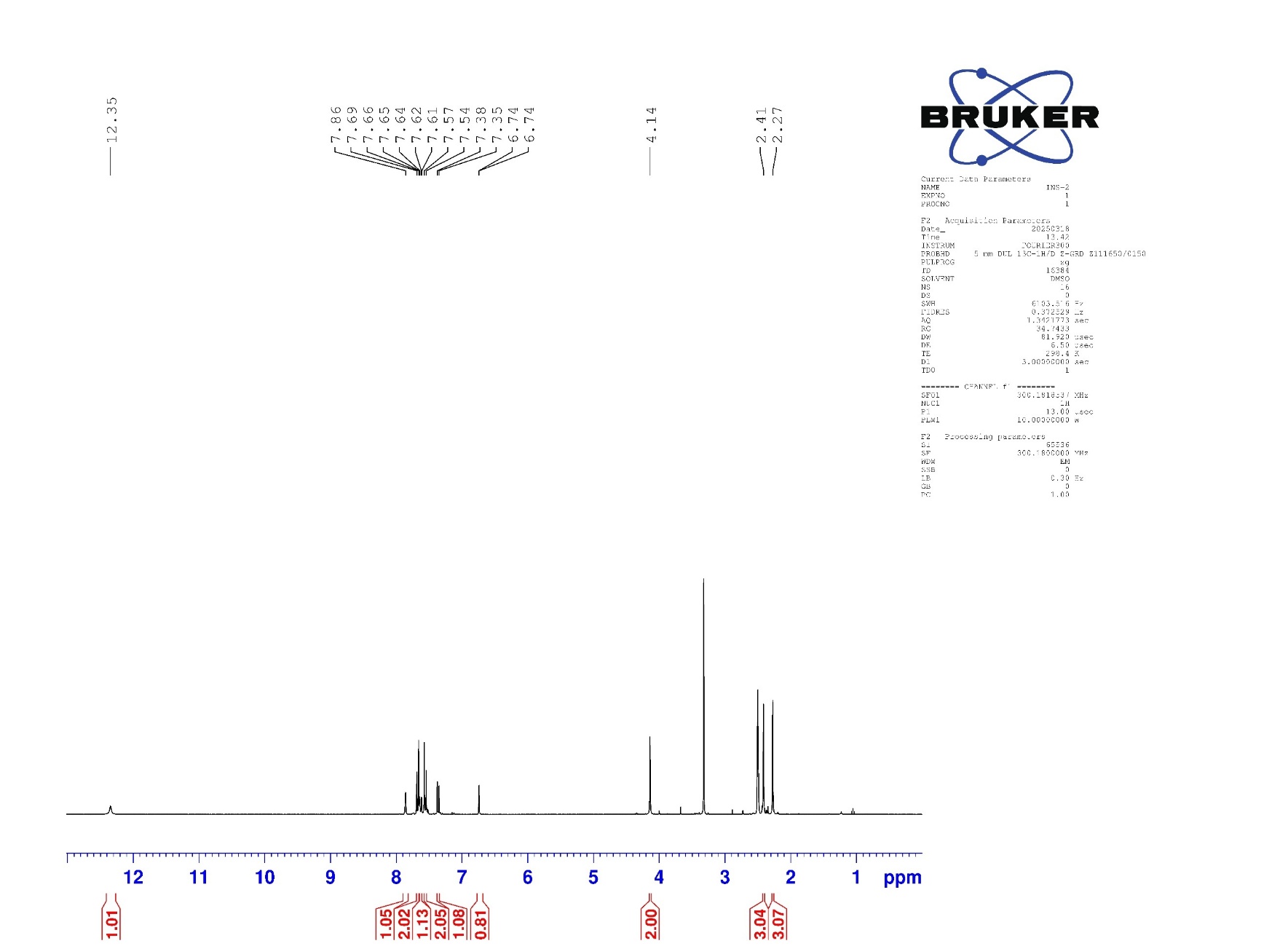


**Figure S7.** ^1^H-NMR spectrum of compound **4b**


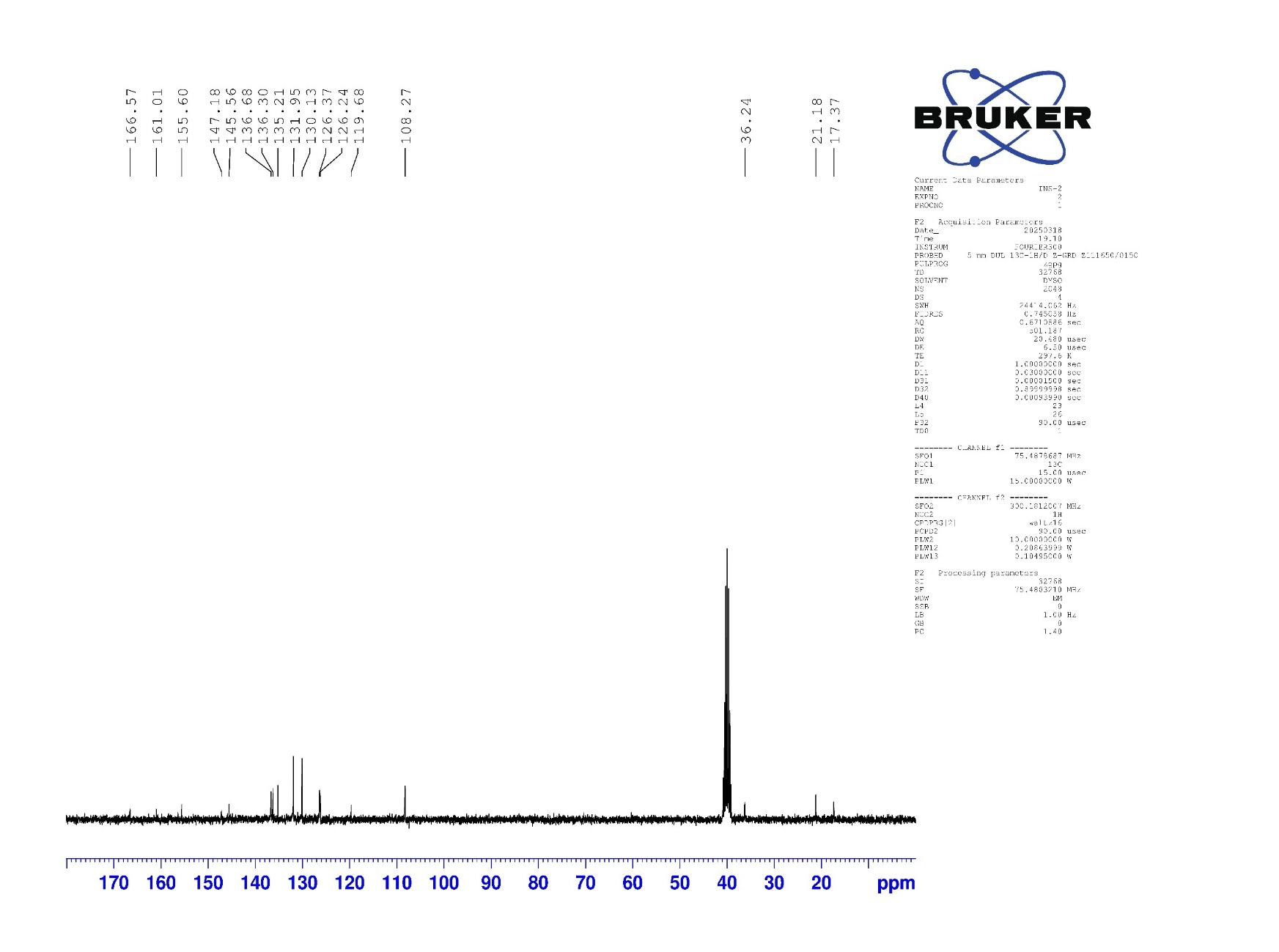


**Figure S8.** ^13^C-NMR spectrum of compound **4b**

***2-((3-(4-Chlorophenyl)-6-methyl-4-oxo-3,4-dihydroquinazoline-2-yl)thio)-N-(6-methylbenzothiazole-2-yl)acetamide (4c)***


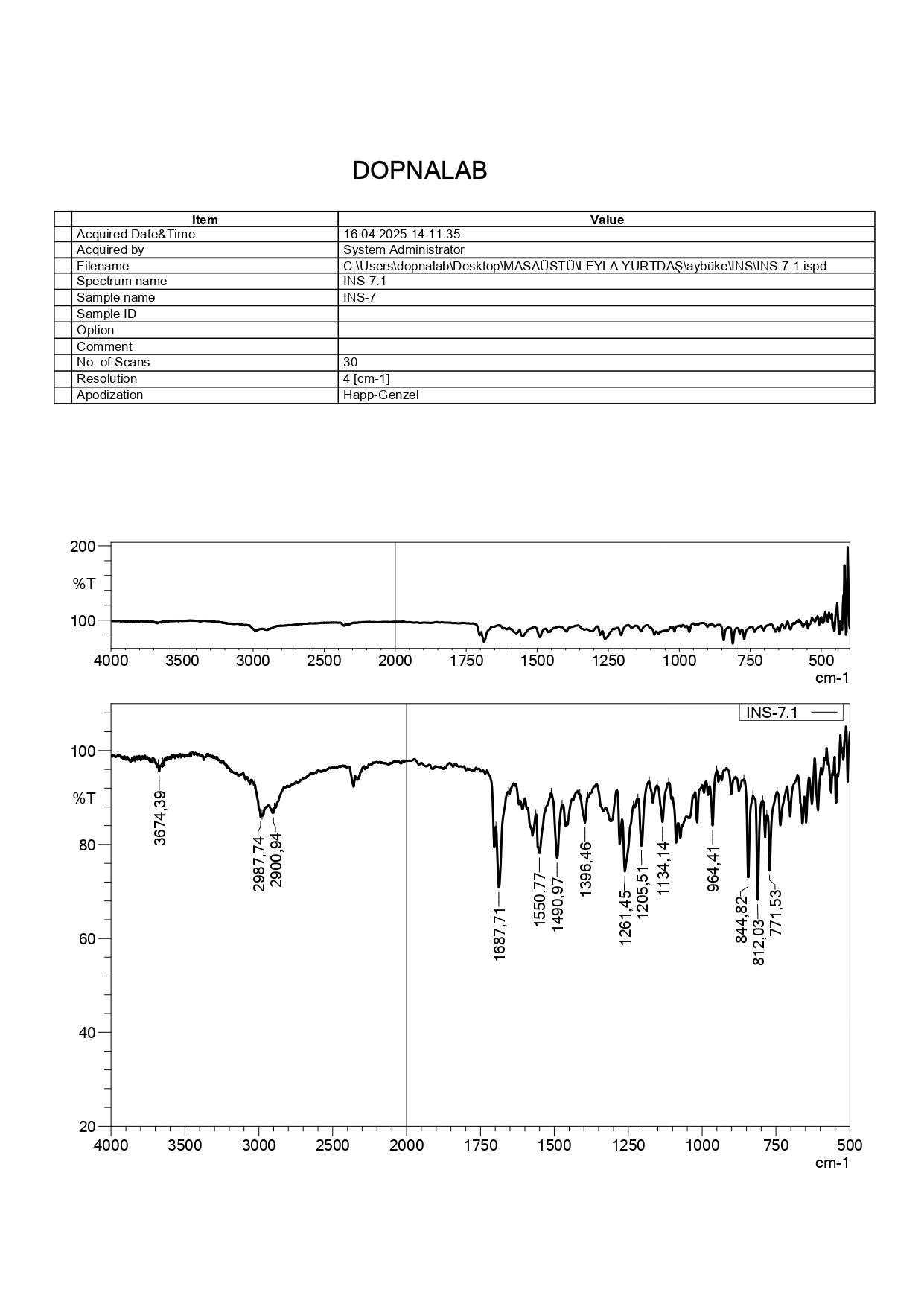


**Figure S9.** IR spectrum of compound **4c**


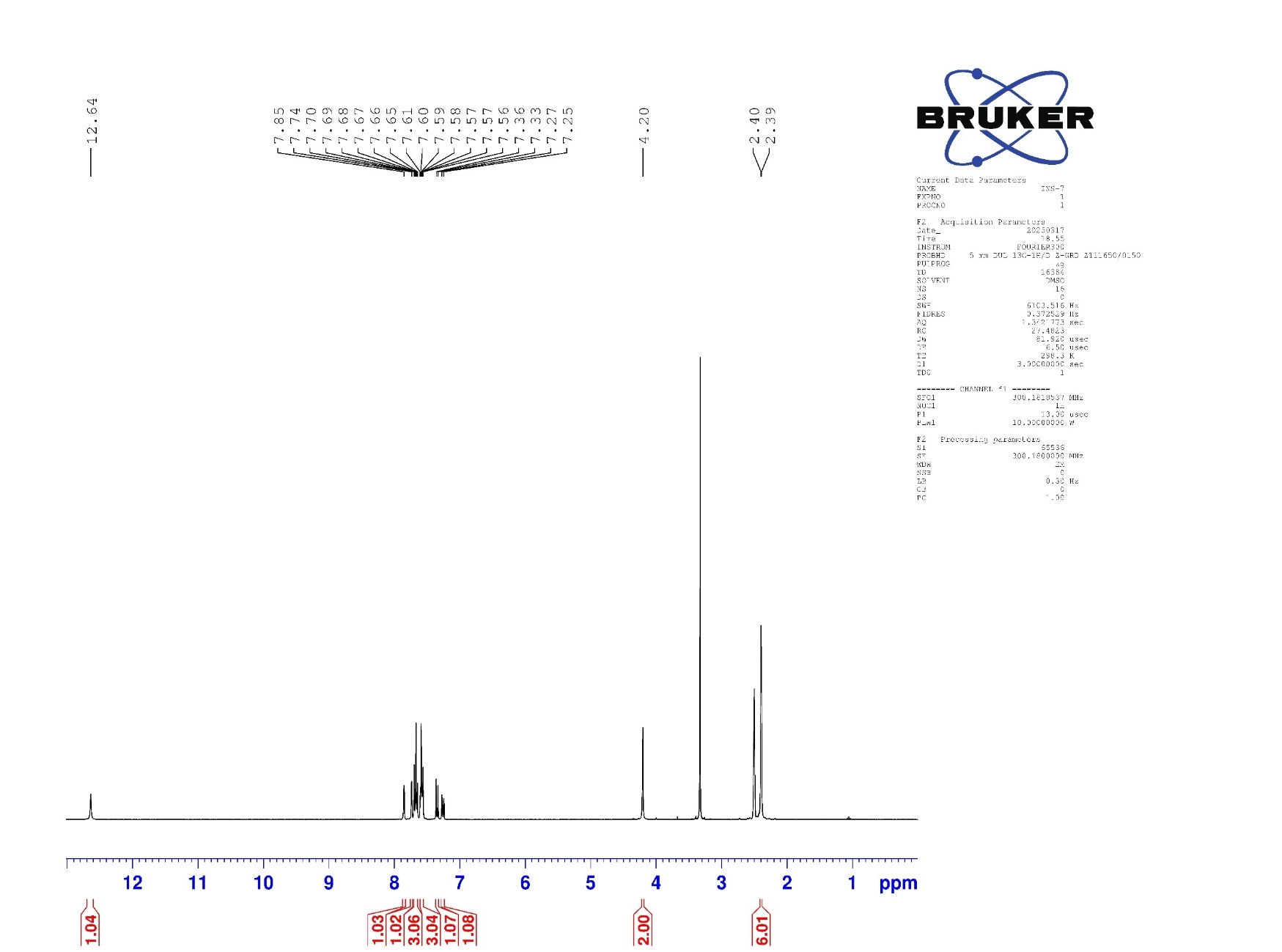


Figure S10. ^1^H-NMR spectrum of compound 4c


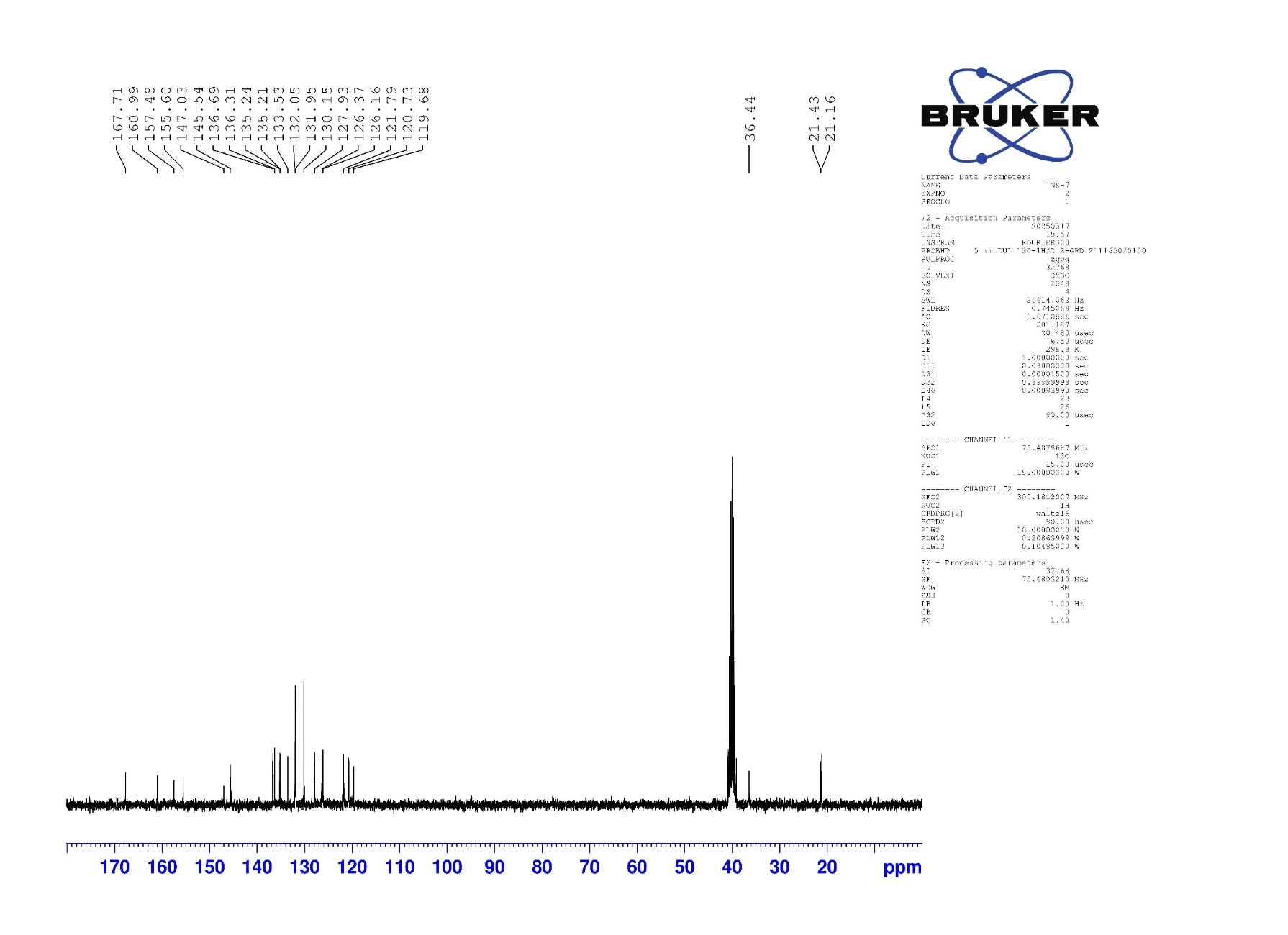


Figure S11. ^13^C-NMR spectrum of compound 4c

***N-(6-Chlorobenzothiazole-2-yl)-2-((3-(4-chlorophenyl)-6-methyl-4-oxo-3,4-dihydroquinazoline-2-yl)thio)acetamide (4d)***


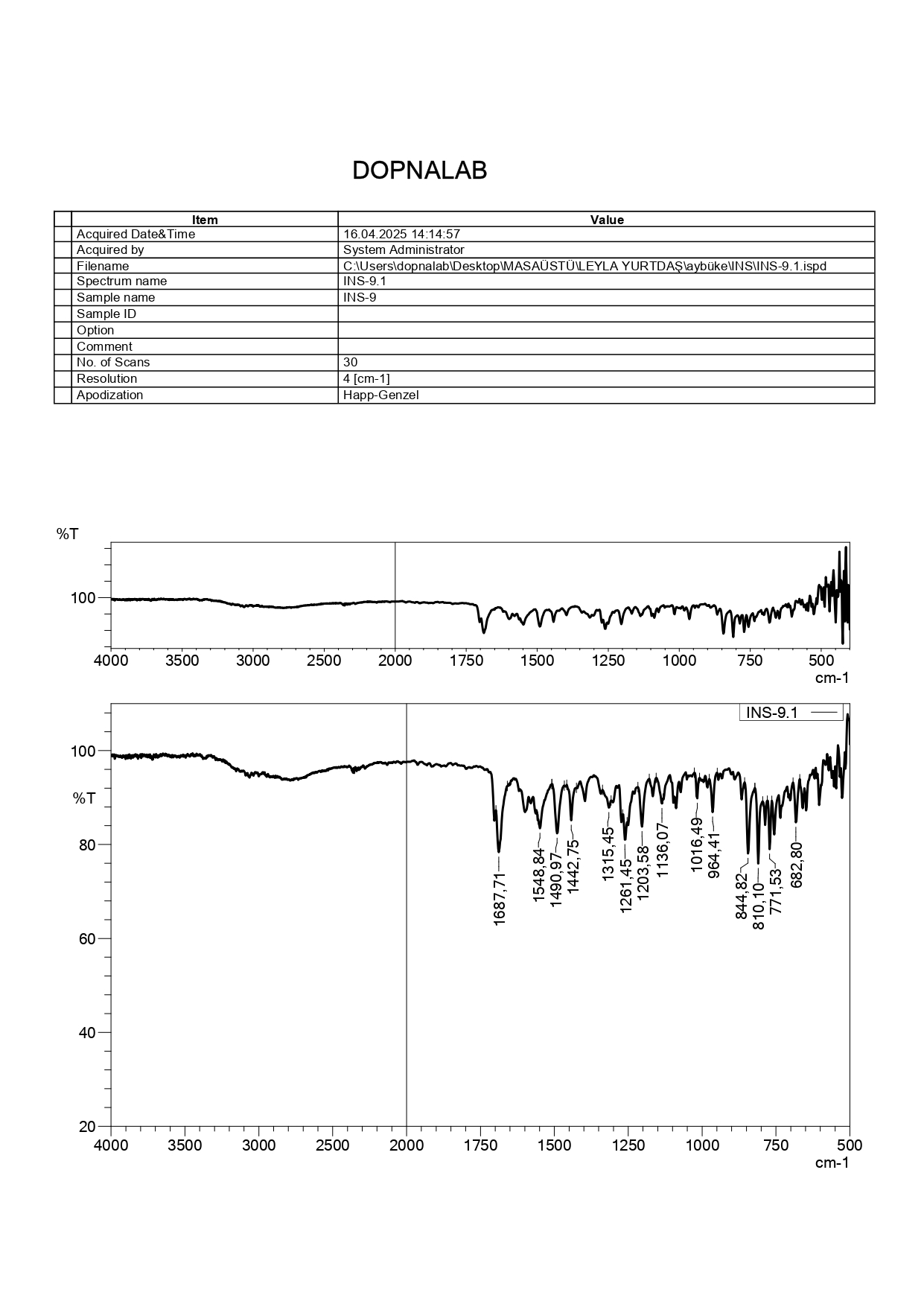


**Figure S12.** IR spectrum of compound **4d**

*
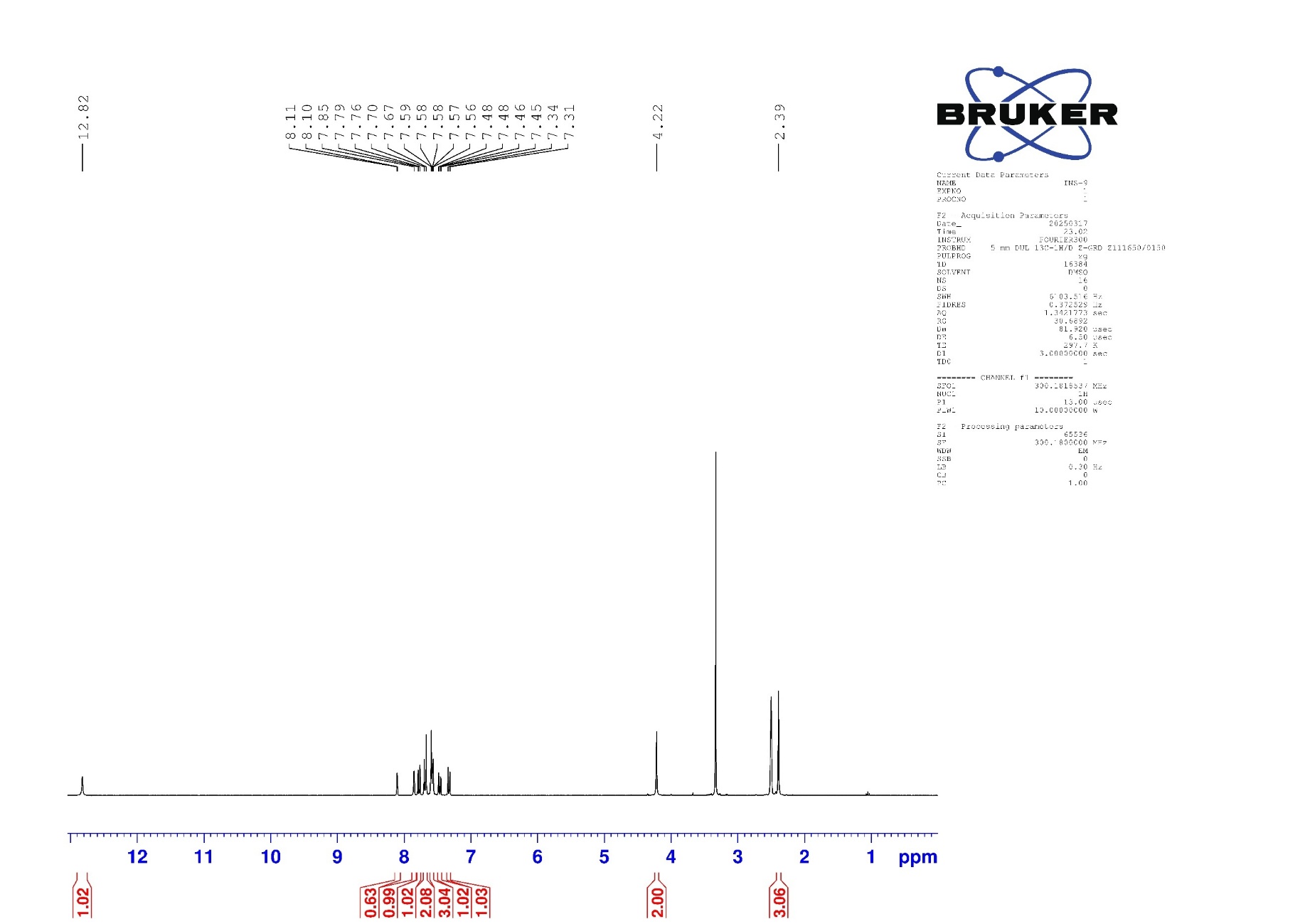
*

**Figure S13.** ^1^H-NMR spectrum of compound **4d**

*
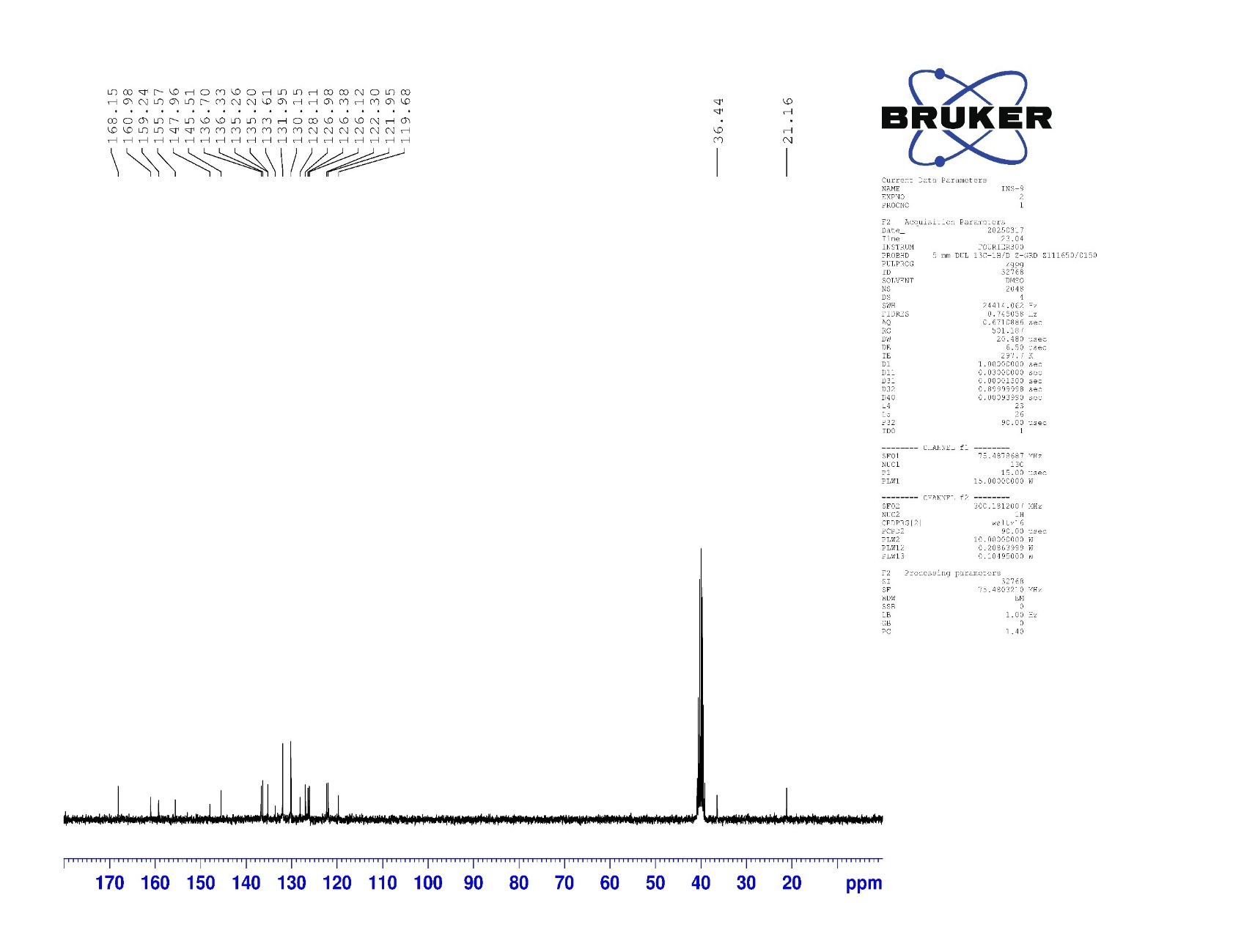
*

Figure S14. ^13^C-NMR spectrum of compound 4d

***2-((3-(4-Chlorophenyl)-5-methyl-4-oxo-3,4-dihydroquinazoline-2-yl)thio)-N-(thiazole-2-yl)acetamide (4e)***


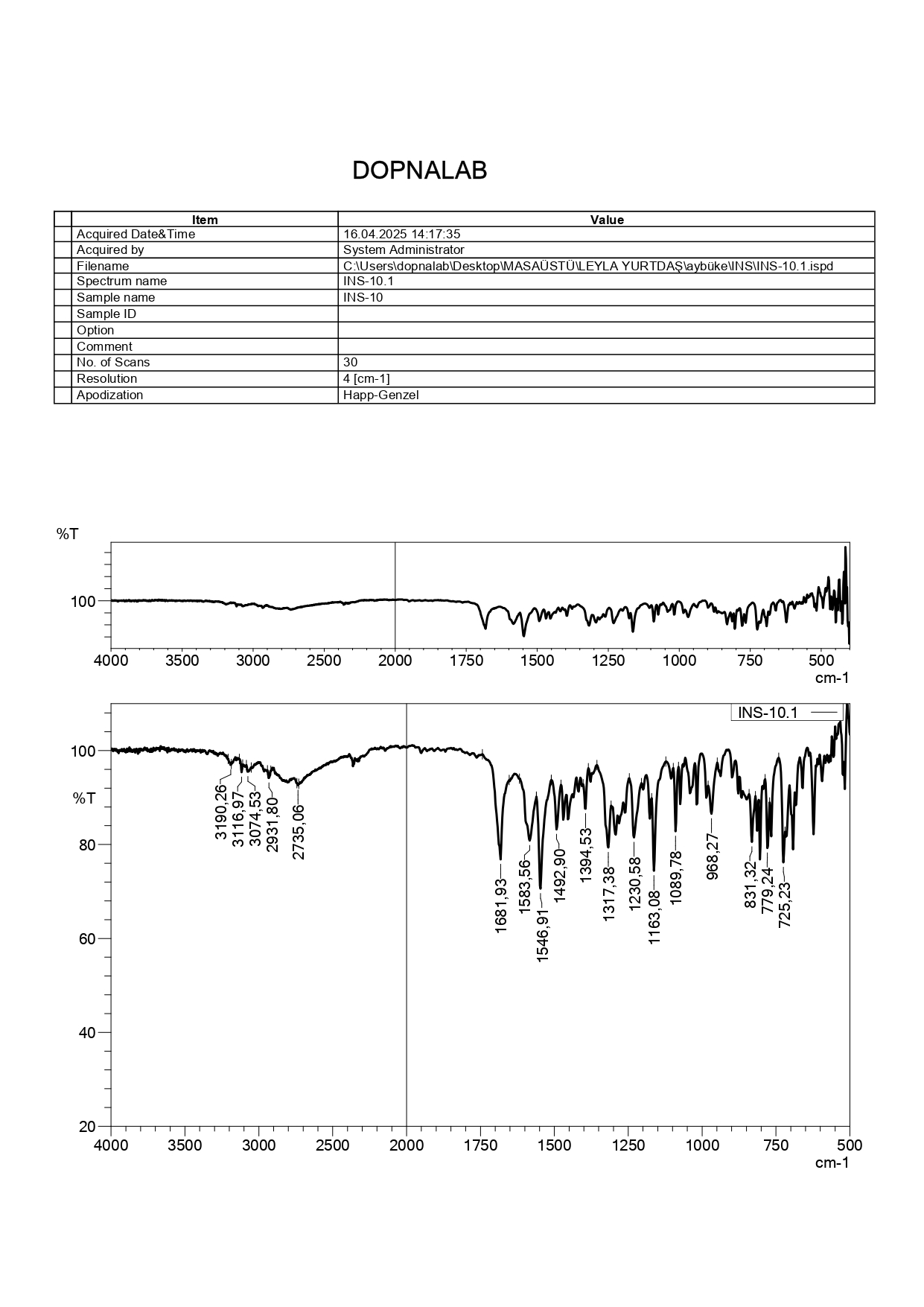


**Figure S15**. IR spectrum of compound **4e**

***
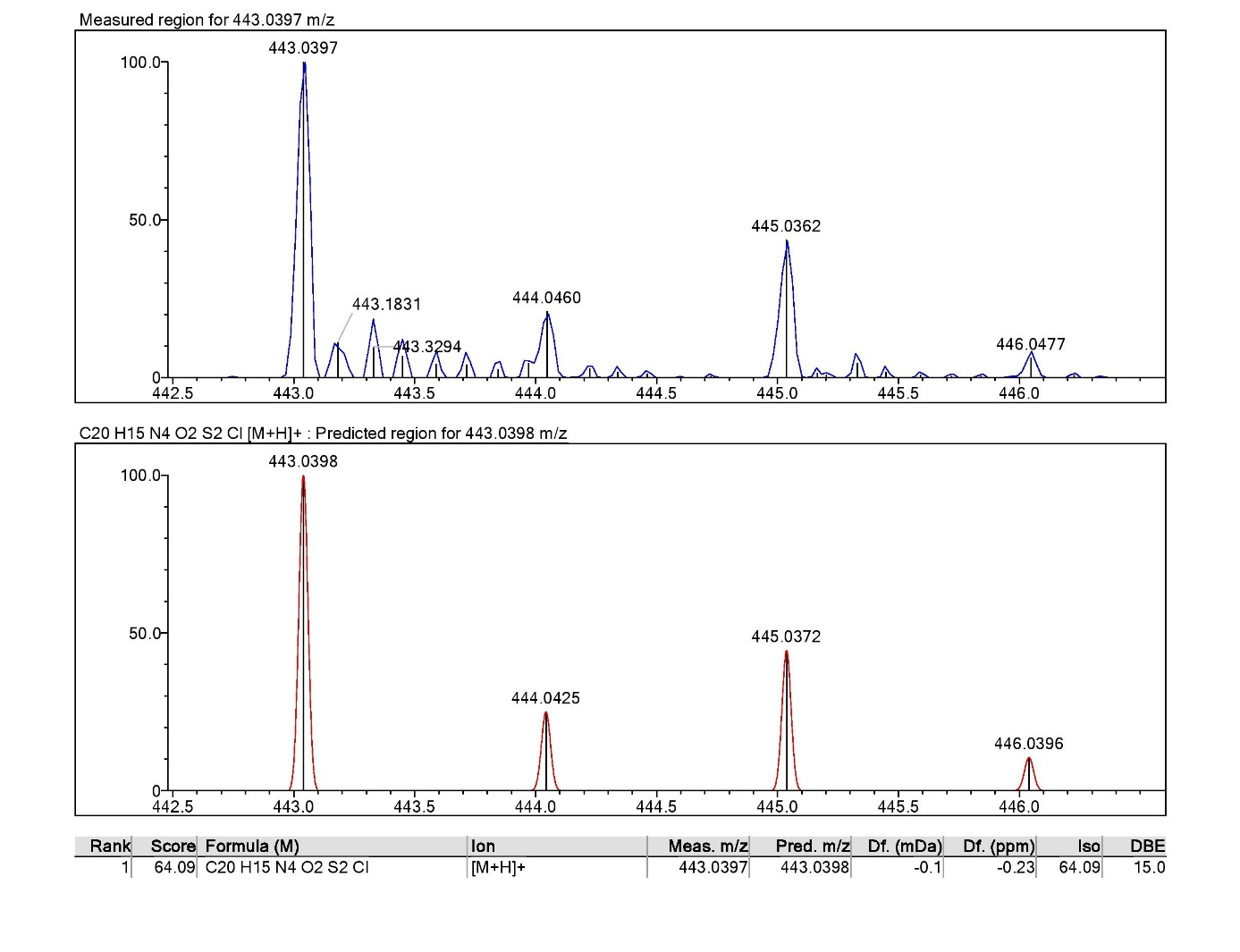
***

**Figure S16.** HRMS spectrum of compound **4e**

*
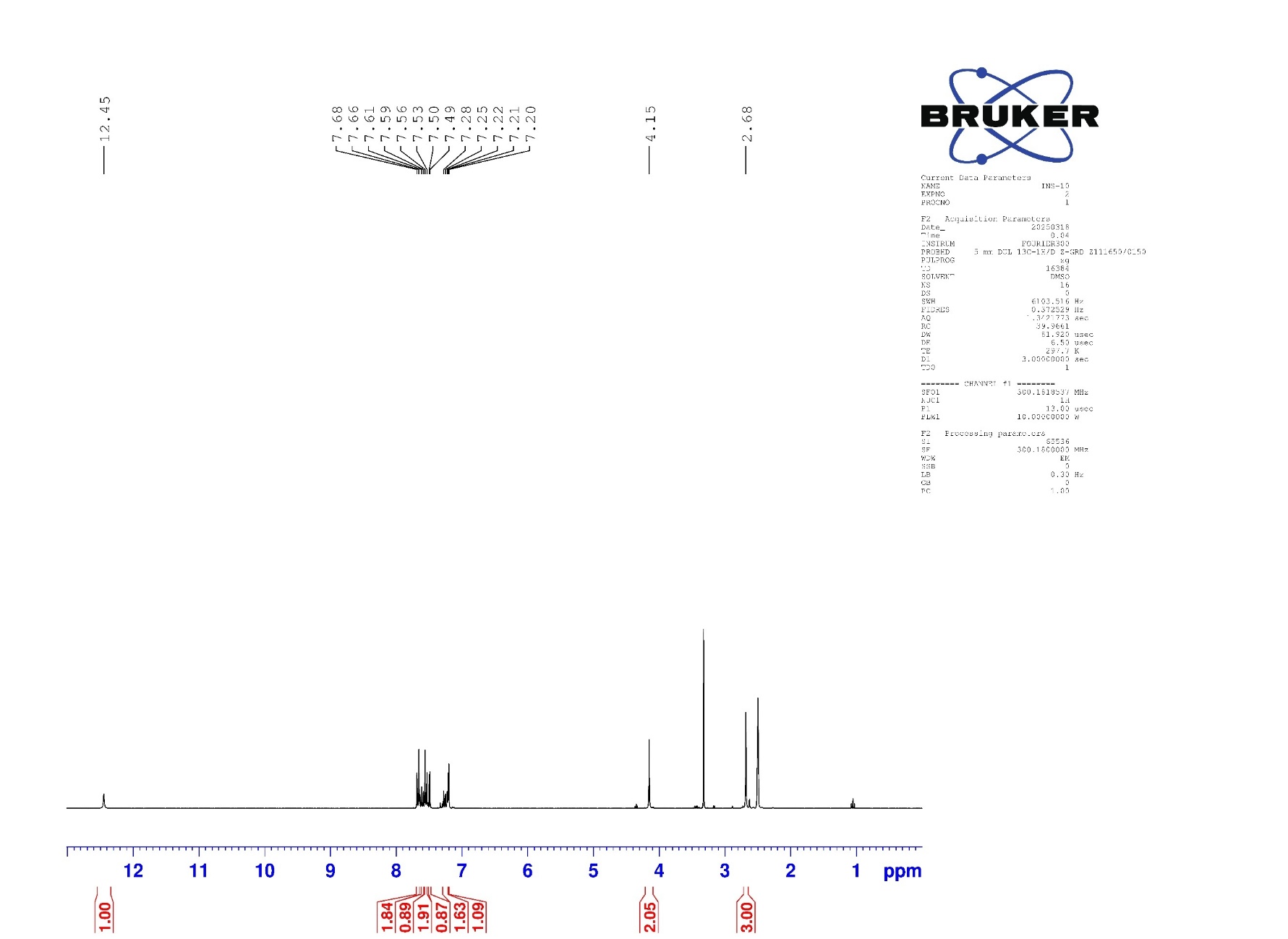
*

Figure S17. ^1^H-NMR spectrum of compound 4e

*
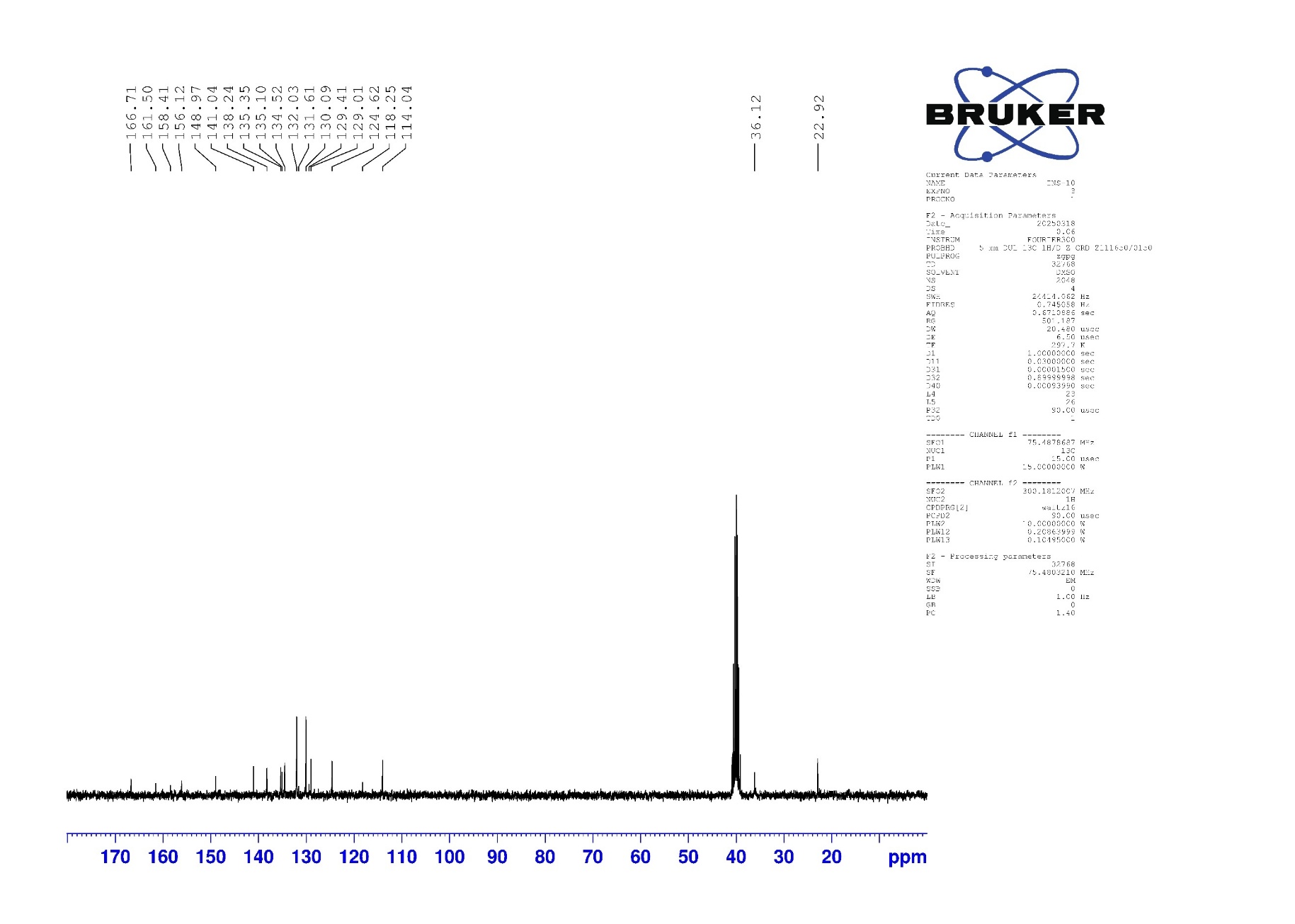
*

Figure S18. ^13^C-NMR spectrum of compound 4e

***2-((3-(4-Chlorophenyl)-5-methyl-4-oxo-3,4-dihydroquinazoline-2-yl)thio)-N-(4,5-dimethylthiazole-2-yl)acetamide (4f)***


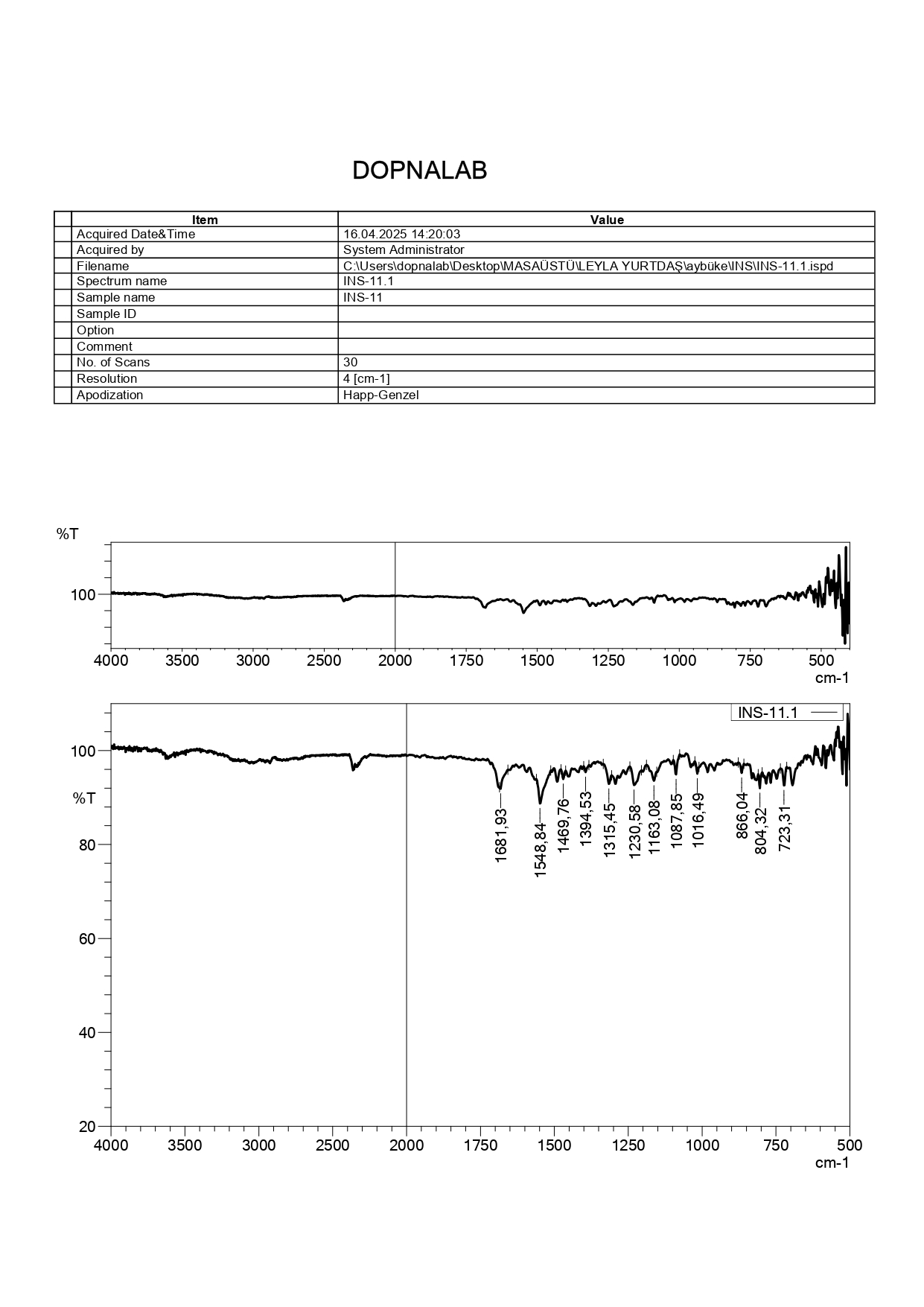


Figure S19. IR spectrum of compound 4f


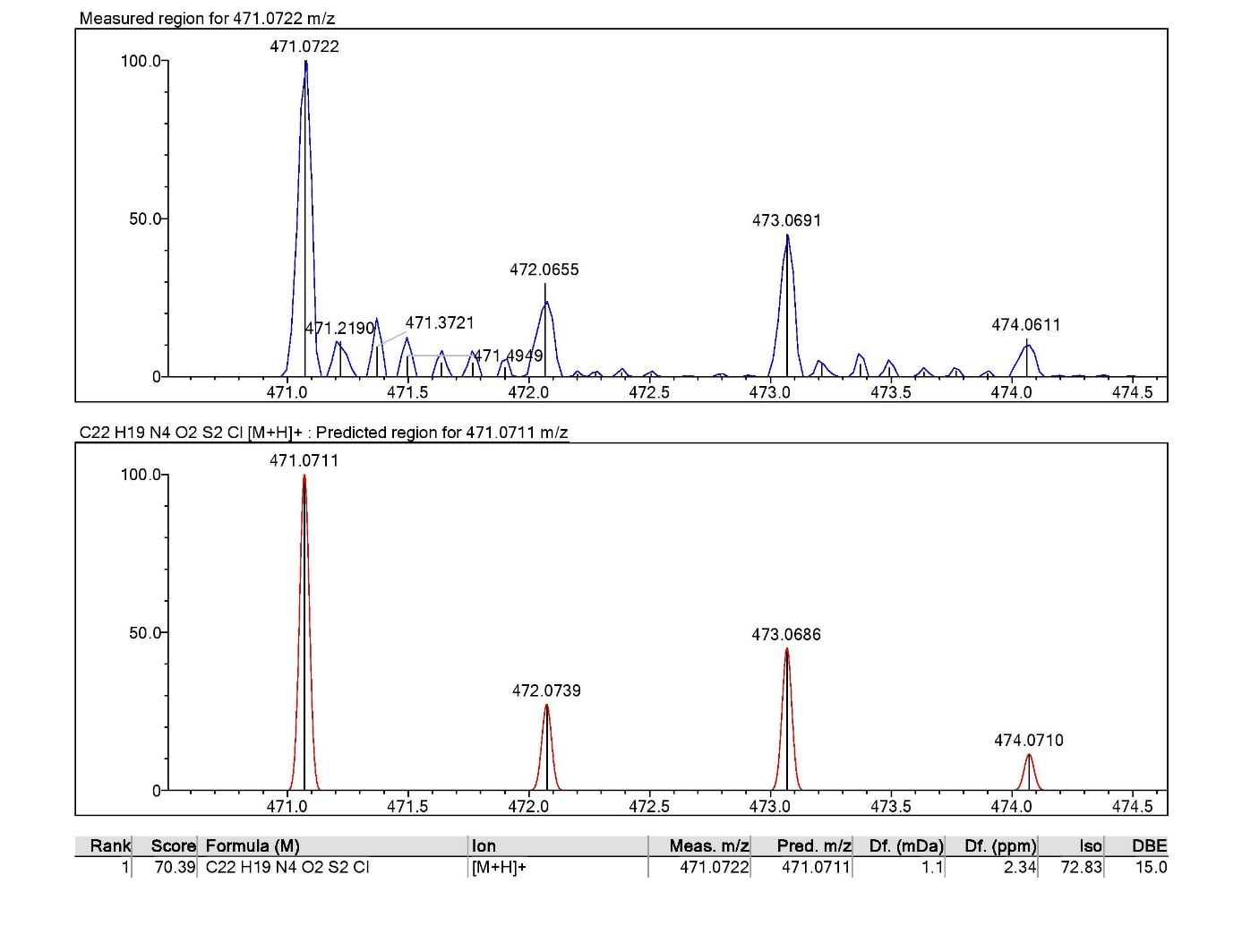


**Figure S20.** HRMS spectrum of **4f**

*
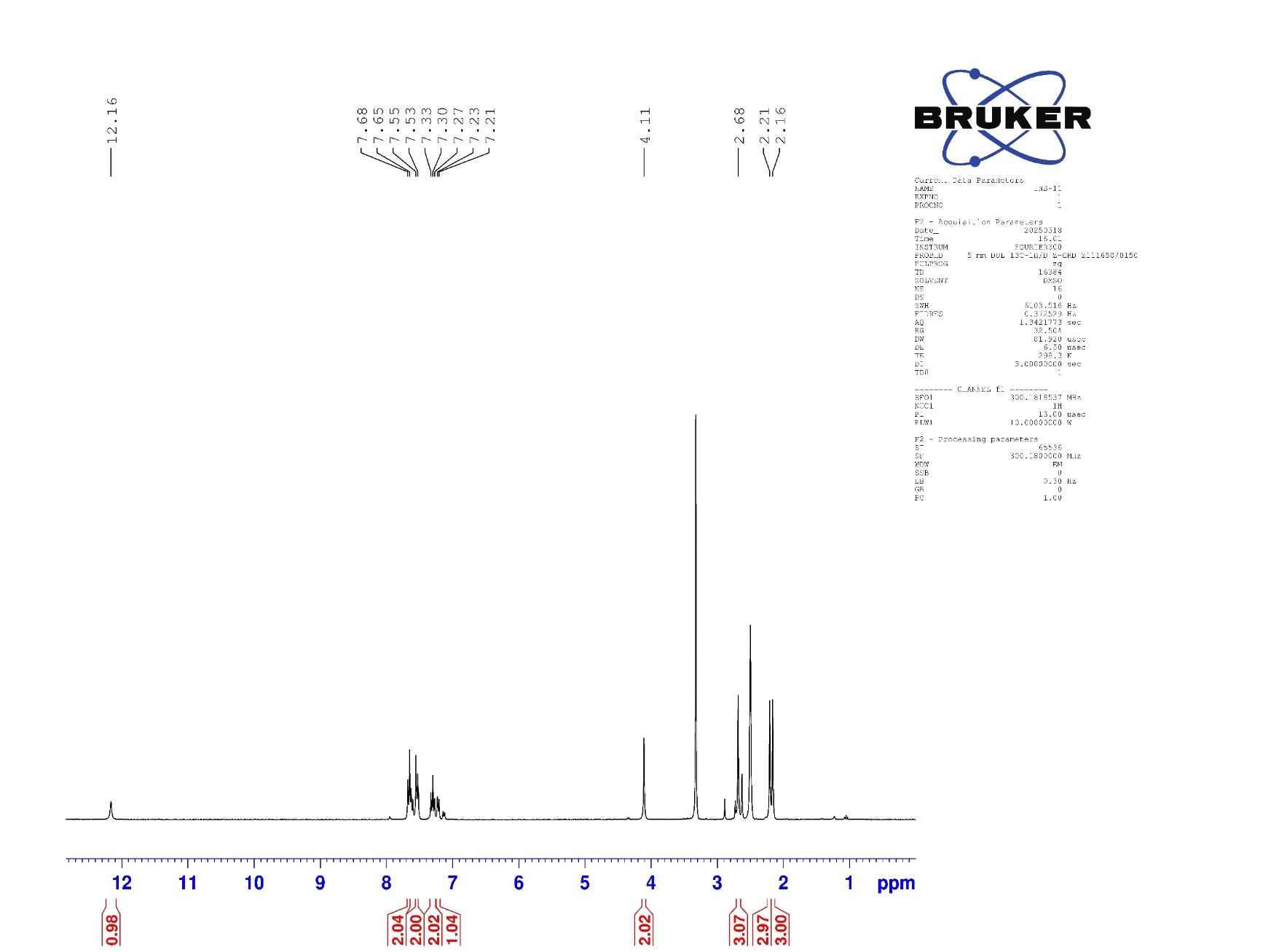
*

**Figure S21.** ^1^H-NMR spectrum of compound **4f**


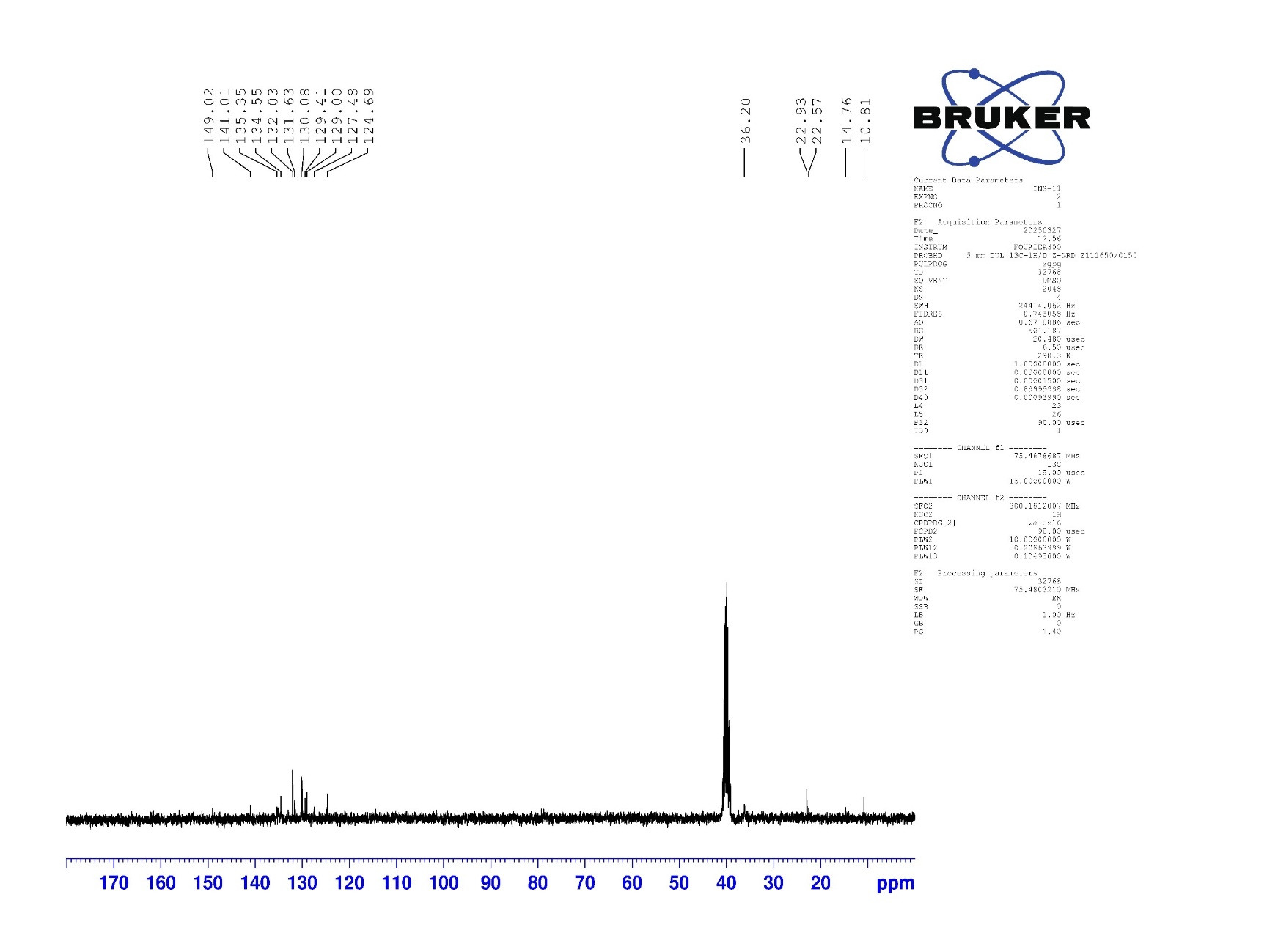


Figure S22. ^13^C-NMR spectrum of compound 4f

***2-((3-(4-Chlorophenyl)-5-methyl-4-oxo-3,4-dihydroquinazoline-2-yl)thio)-N-(4,5-diphenylthiazole-2-yl)acetamide (4g)***


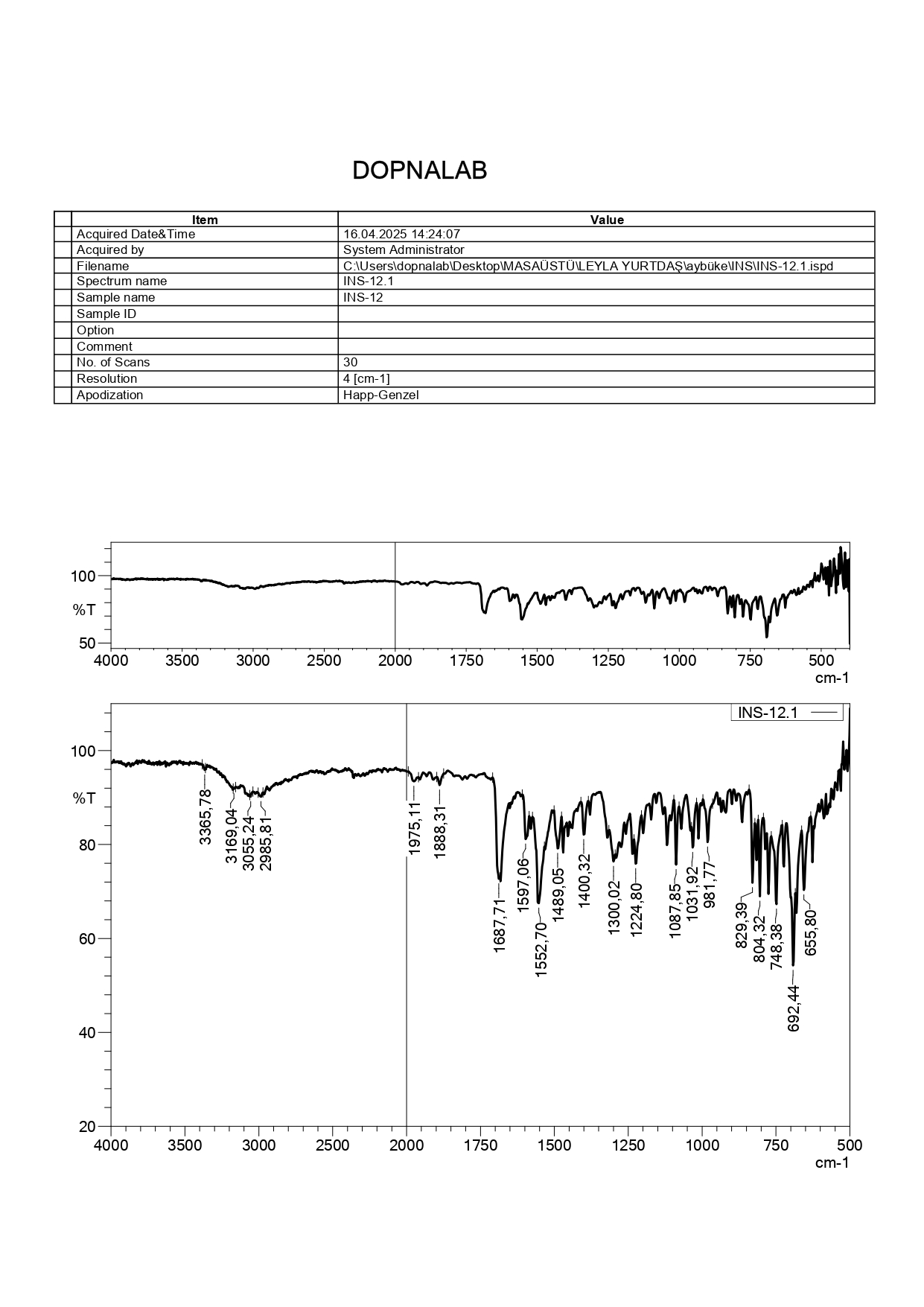


Figure S23. IR spectrum of compound 4g

*
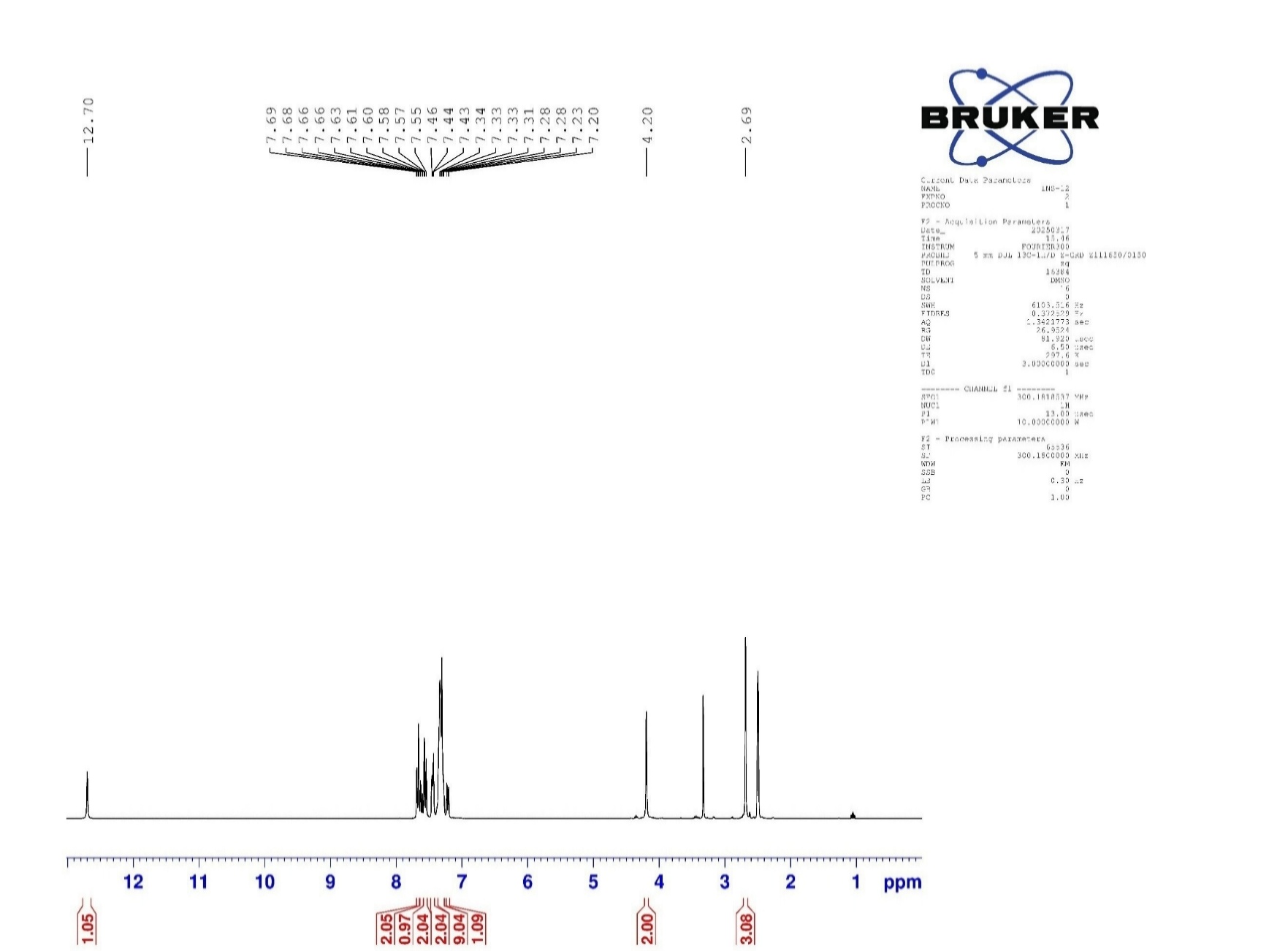
*

Figure S24. ^1^H-NMR spectrum of compound 4g

*
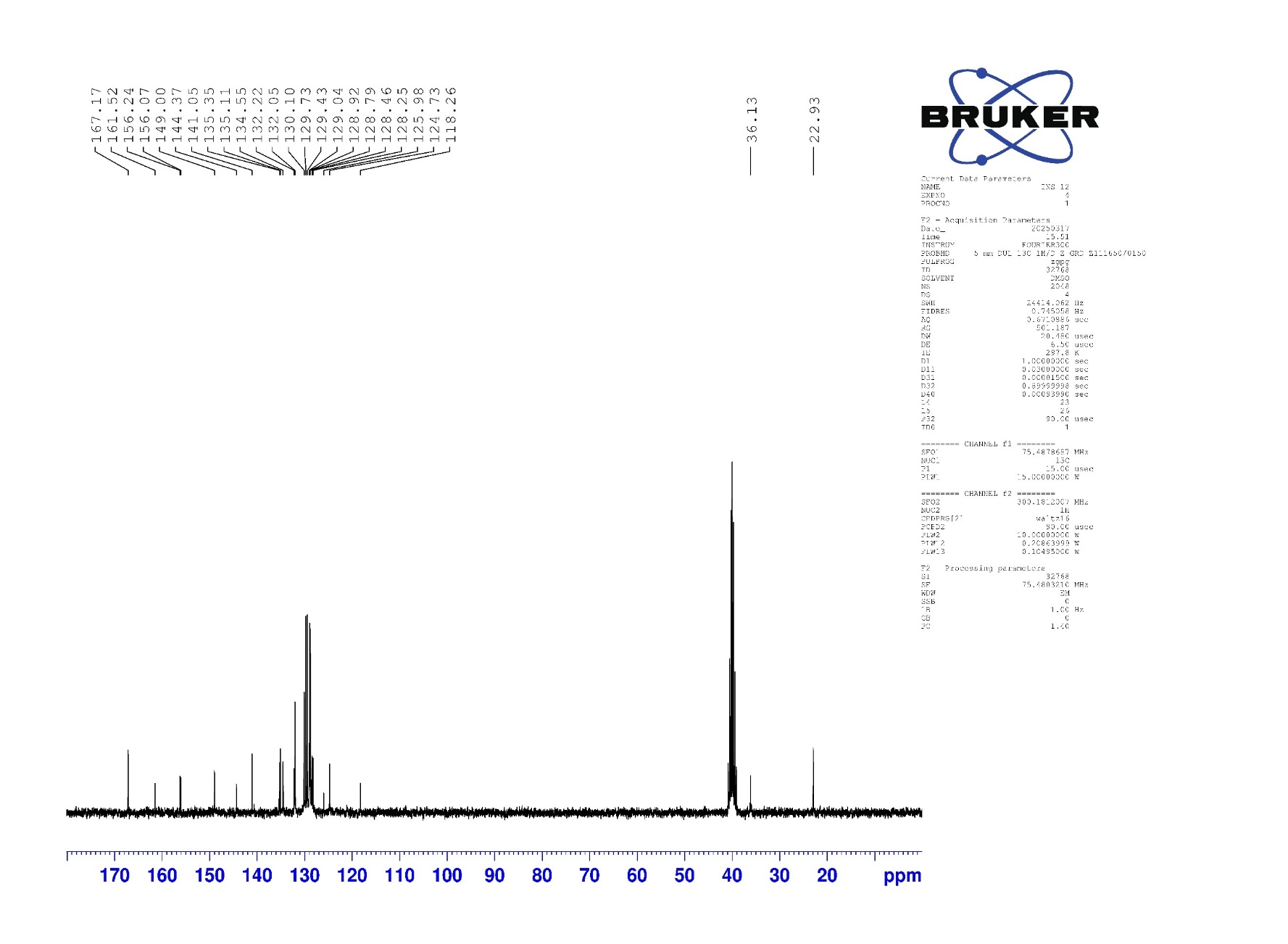
*

Figure S25. ^13^C-NMR spectrum of compound 4g

***2-((3-(4-Chlorophenyl)-5-methyl-4-oxo-3,4-dihydroquinazoline-2-yl)thio)-N-(6-methylbenzothiazole-2-yl)acetamide (4h)***


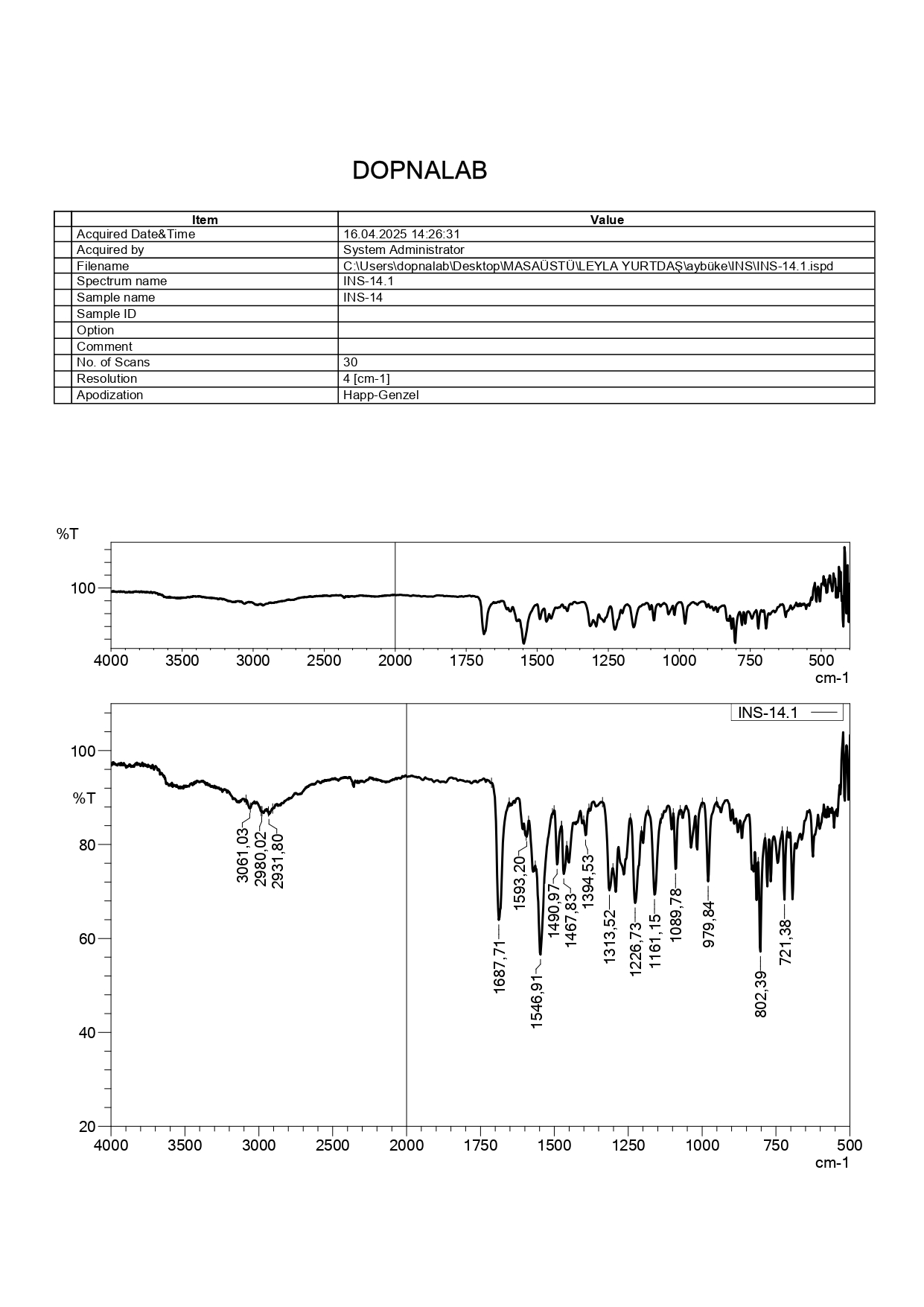


**Figure S26**. IR spectrum of compound **4h**

**
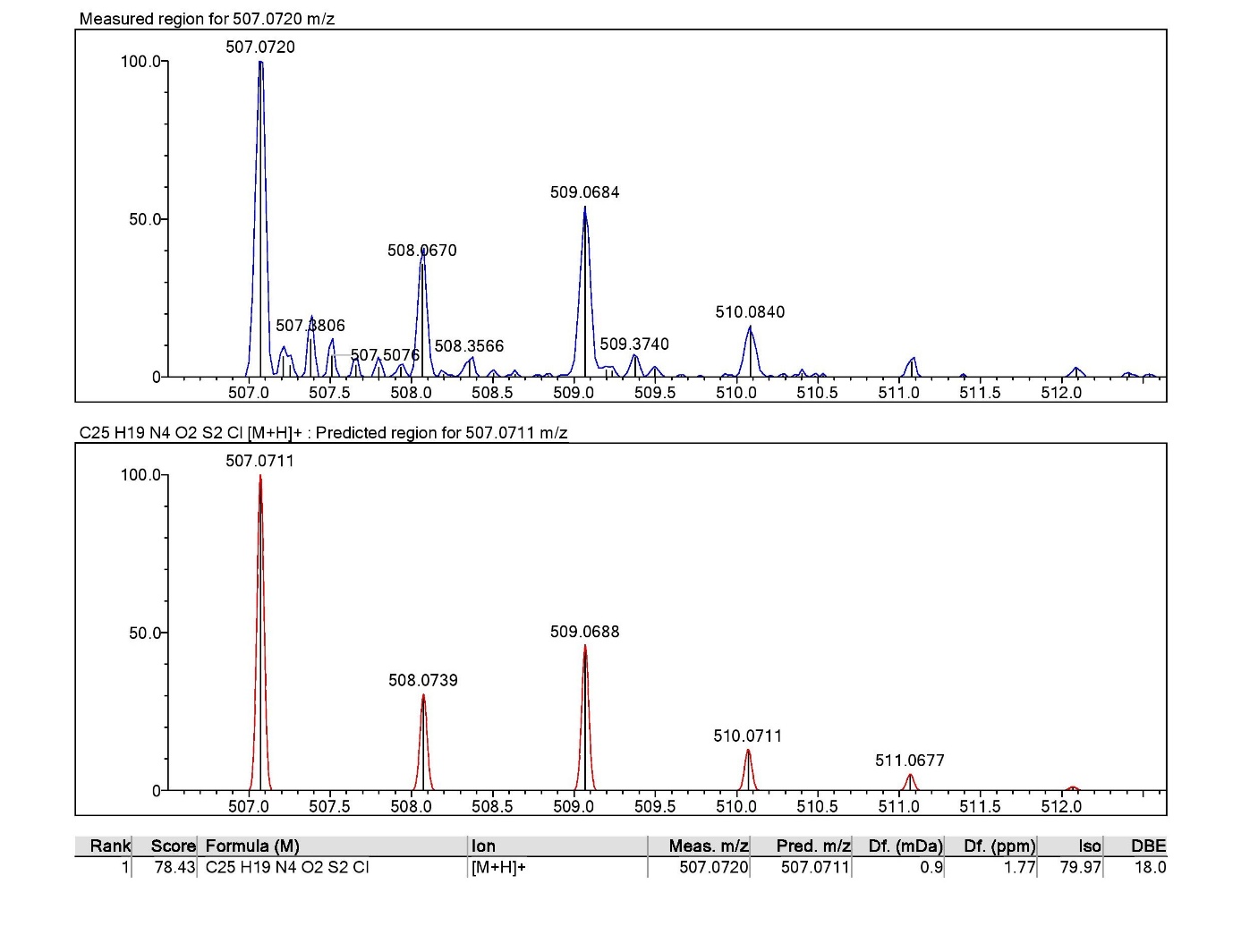
**

**Figure S27.** HRMS spectrum of compound **4h**

*
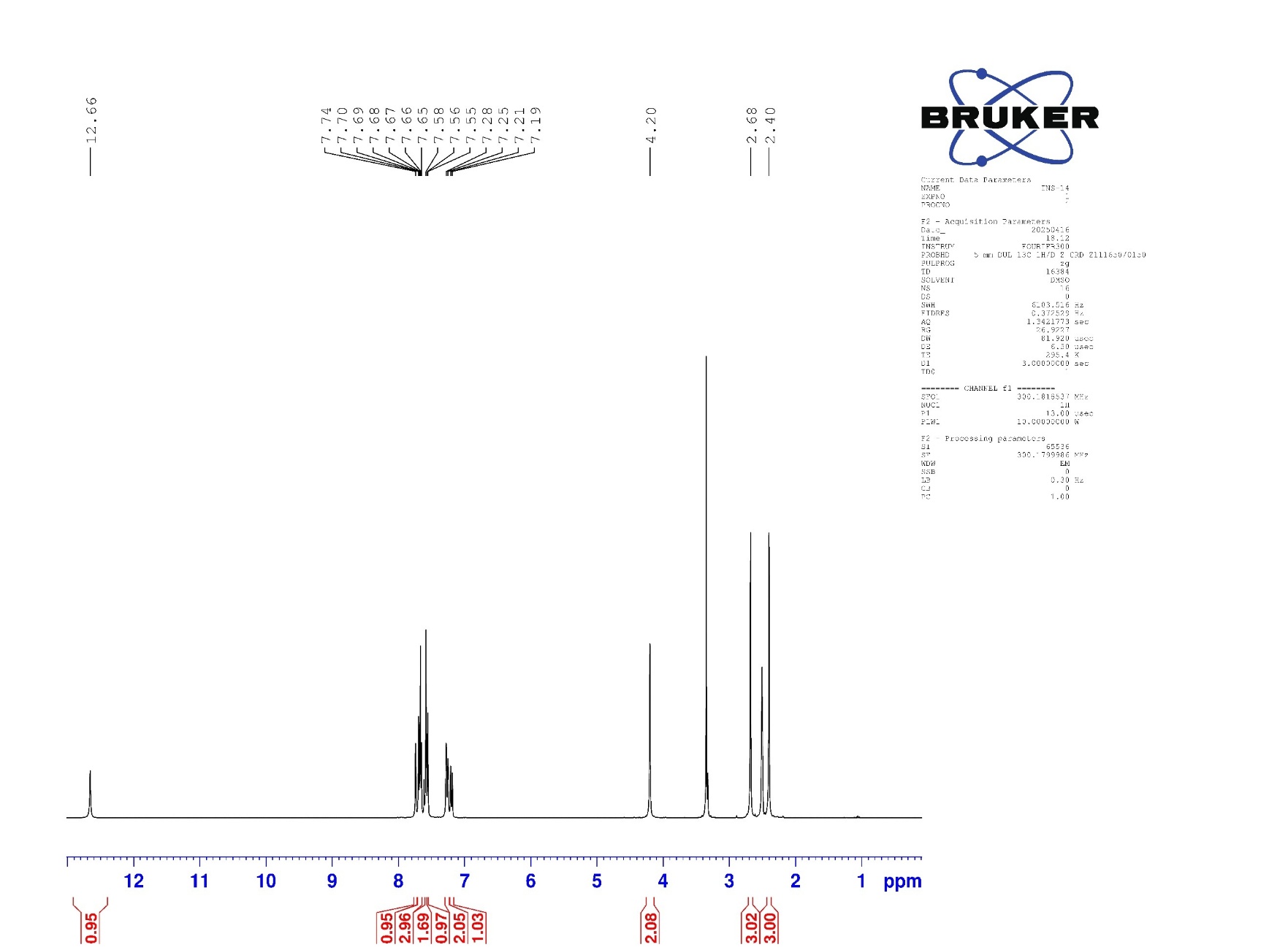
*

Figure S28. ^1^H-NMR spectrum of compound 4h

*
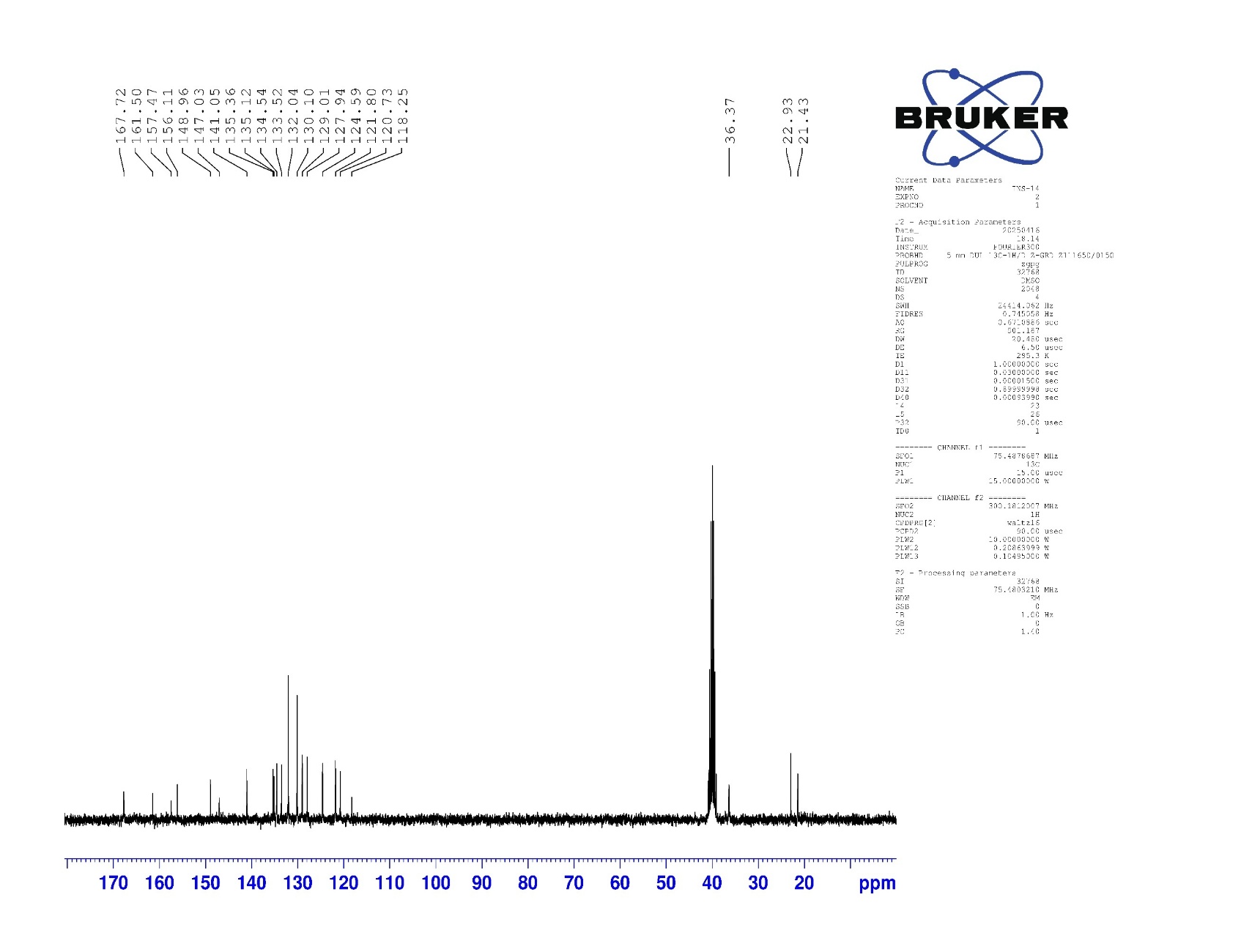
*

Figure S29. ^13^C-NMR spectrum of compound 4h

***2-((3-(4-Chlorophenyl)-4-oxo-3,4-dihydroquinazoline-2-yl)thio)-N-(thiazole-2-yl)acetamide (4i)***


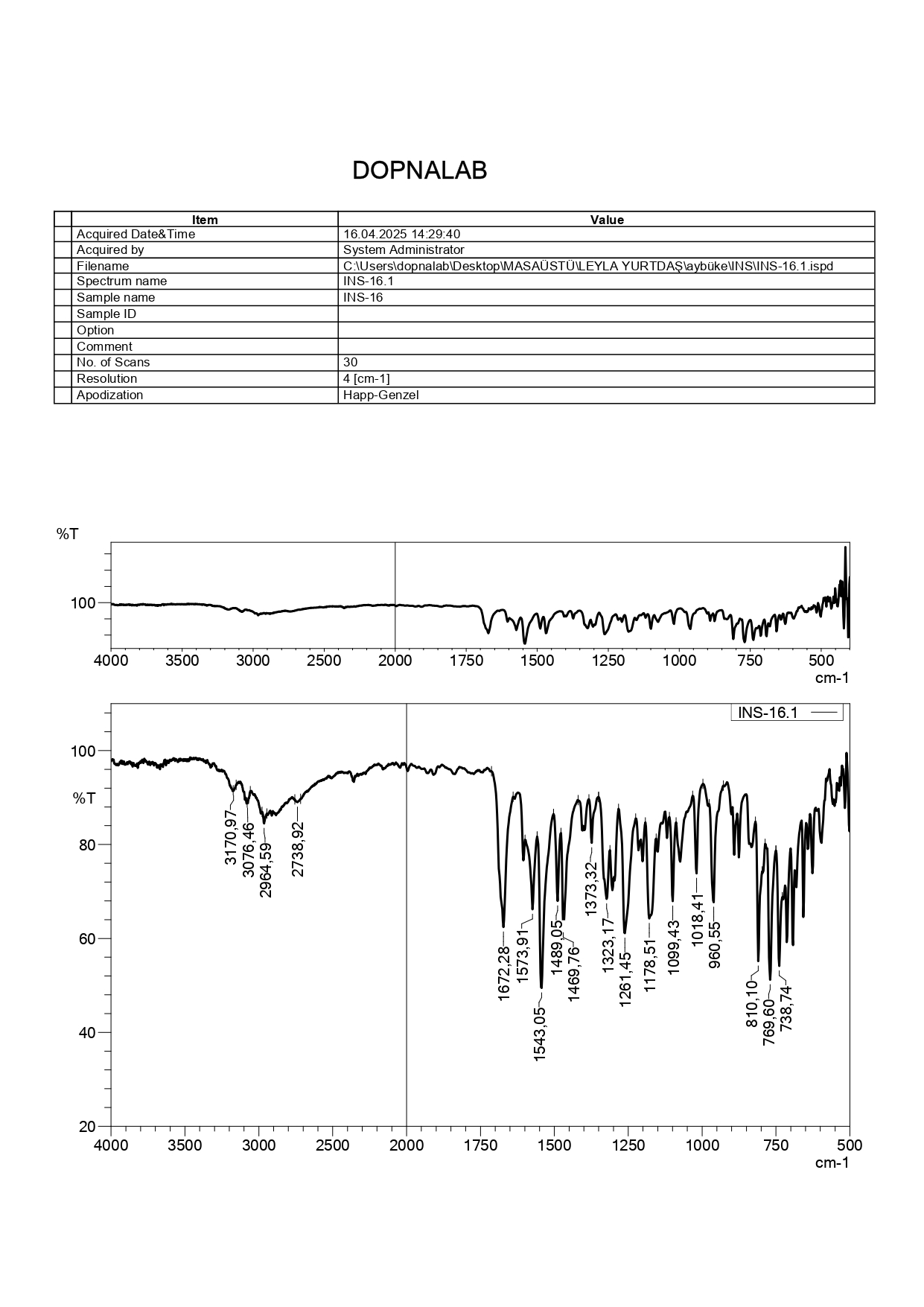


Figure S30. IR spectrum of compound 4i


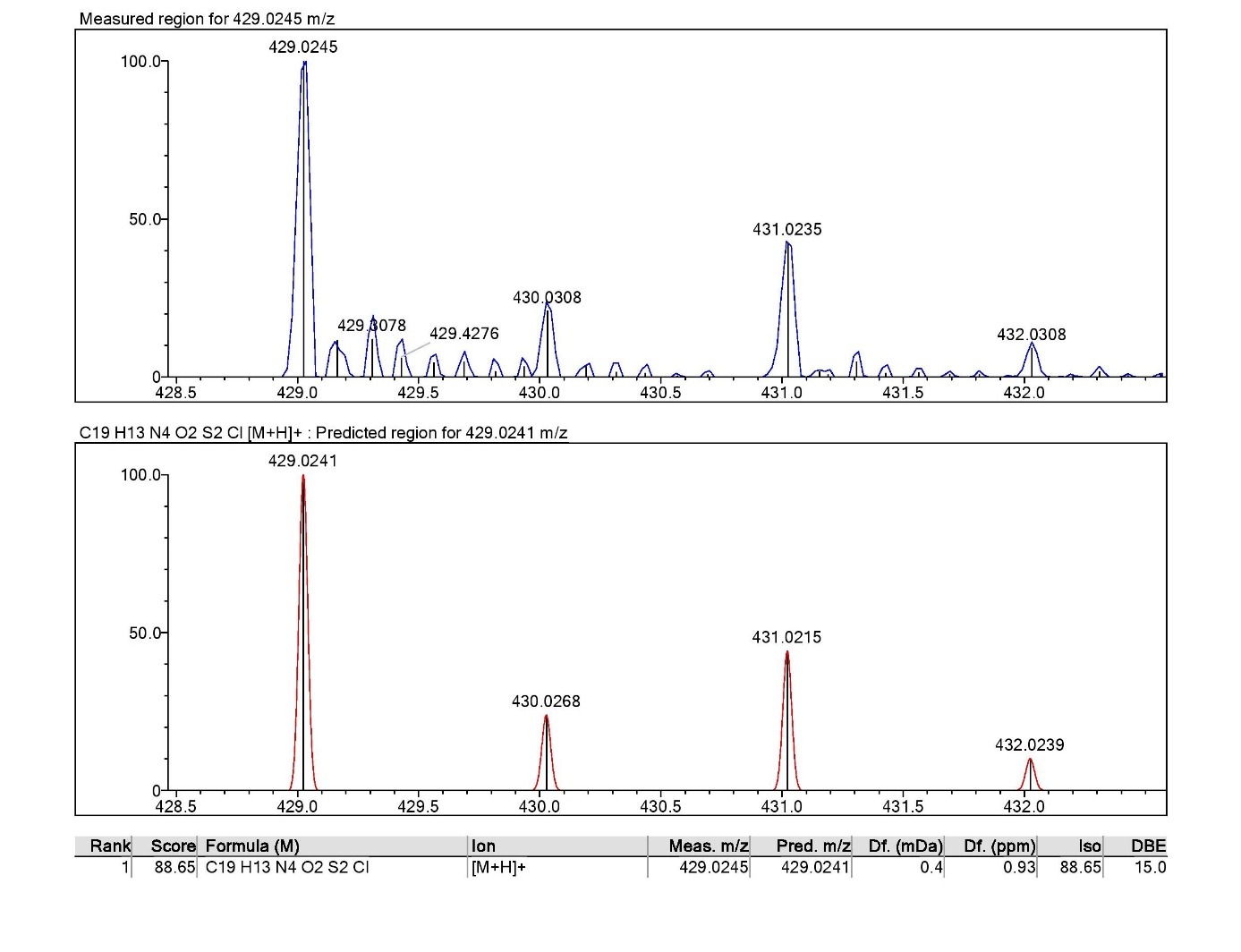


**Figure S31.** HRMS spectrum of compound **4i**

*
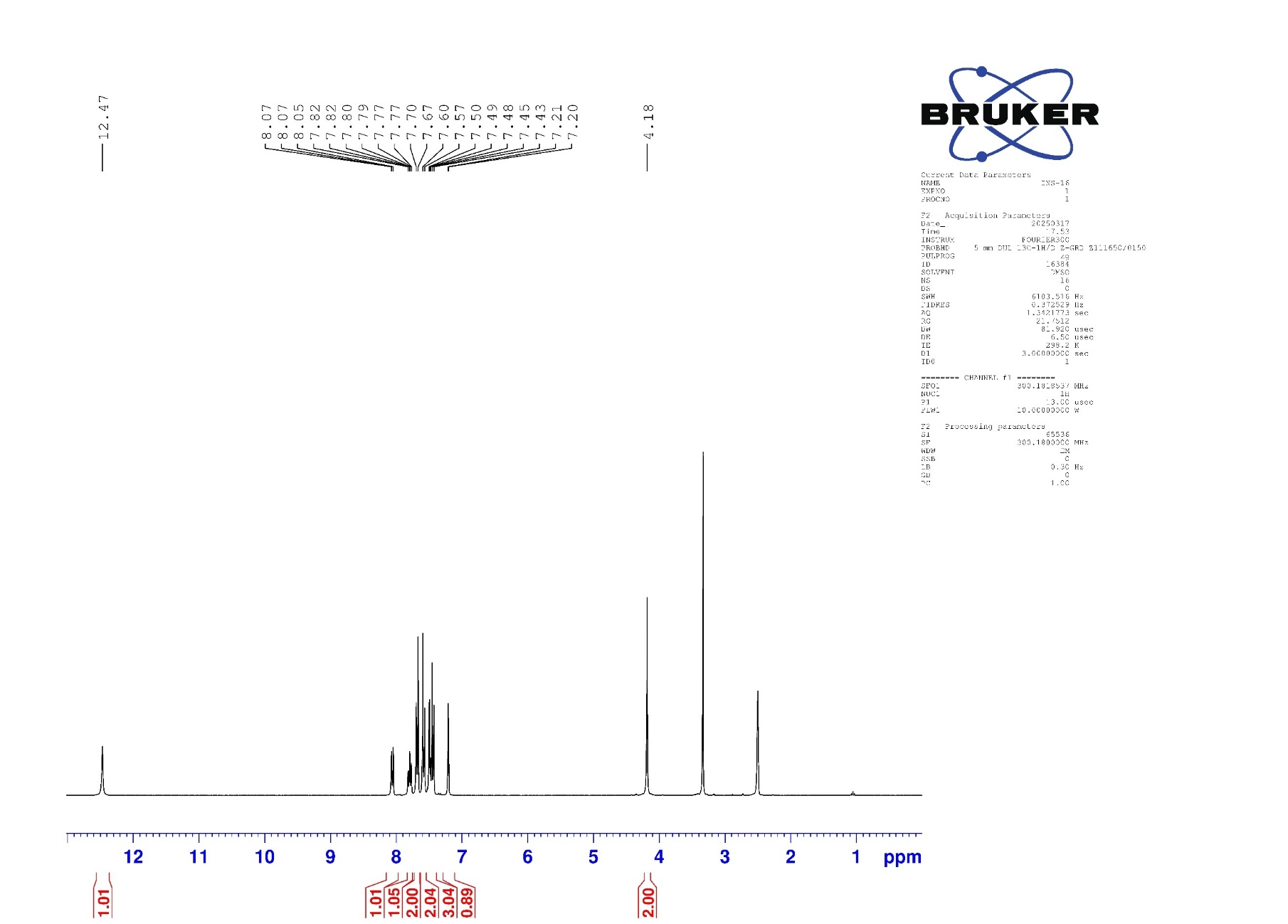
*

Figure S32. ^1^H-NMR spectrum of compound 4i

*
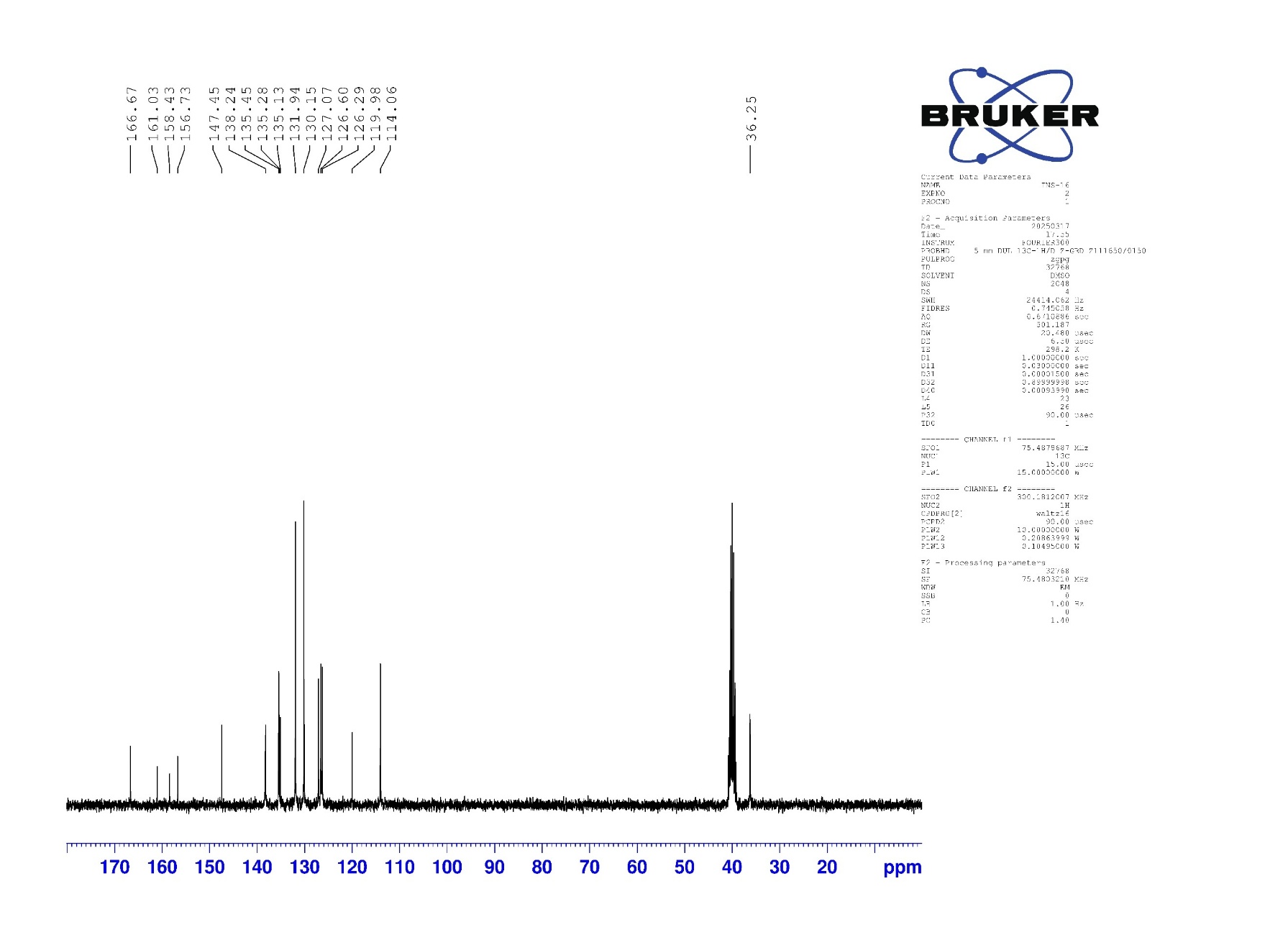
*

Figure S33. ^13^C-NMR spectrum of compound 4i

***2-((3-(4-Chlorophenyl)-4-oxo-3,4-dihydroquinazoline-2-yl)thio)-N-(4,5-dimethylthiazole-2-yl)acetamide (4j)***


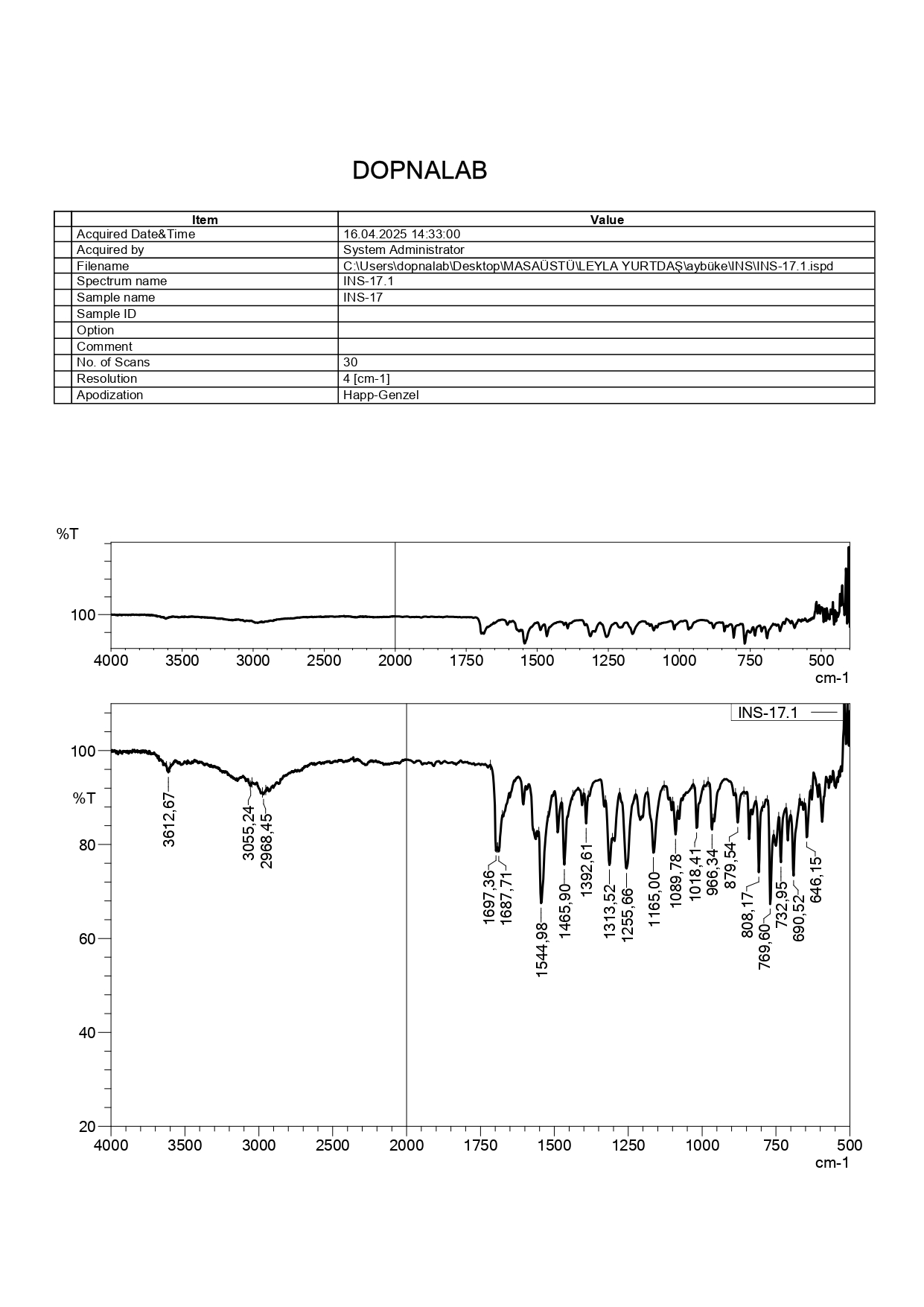


Figure S34. IR spectrum of compound 4j


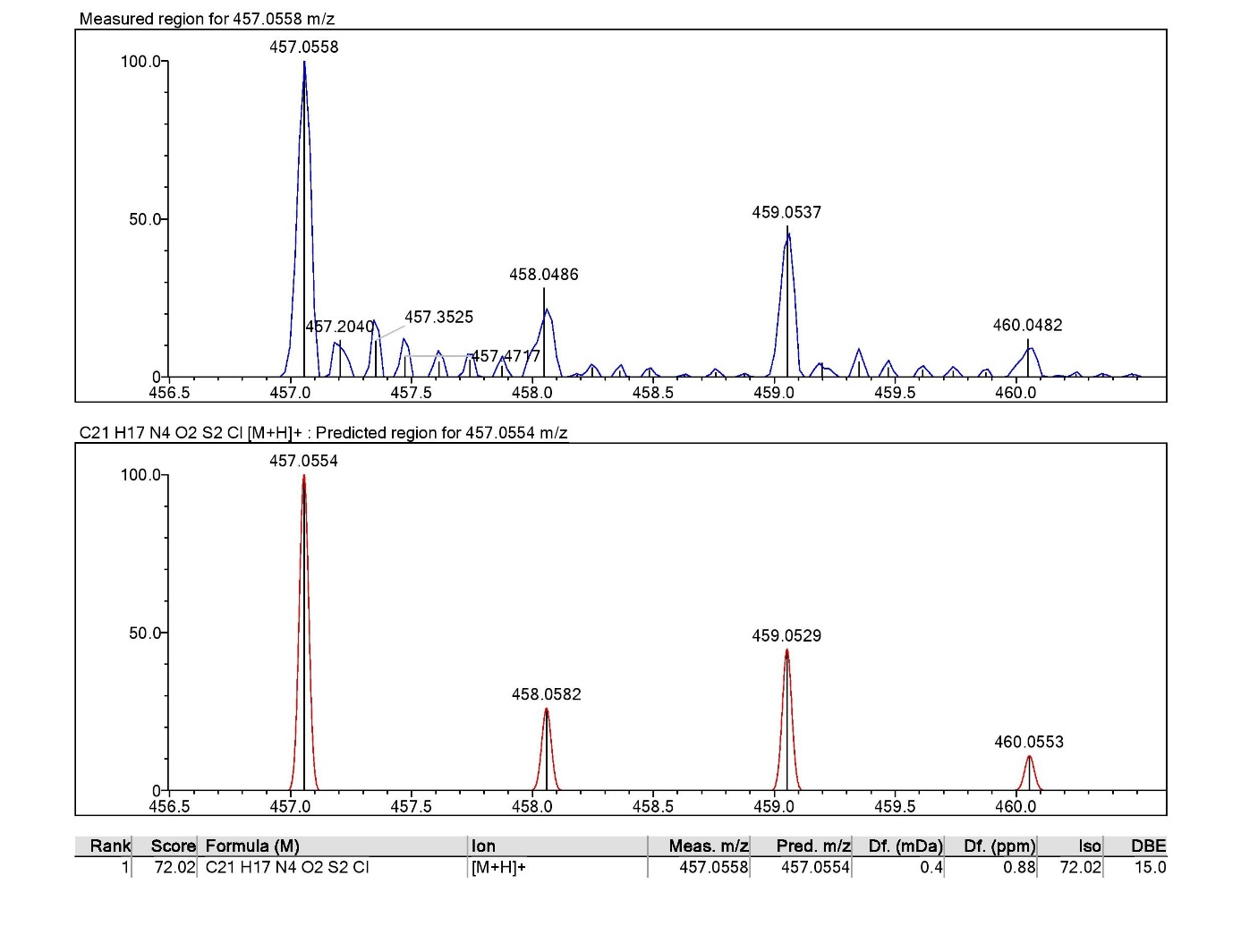


**Figure S35.** HRMS spectrum of compound **4j**


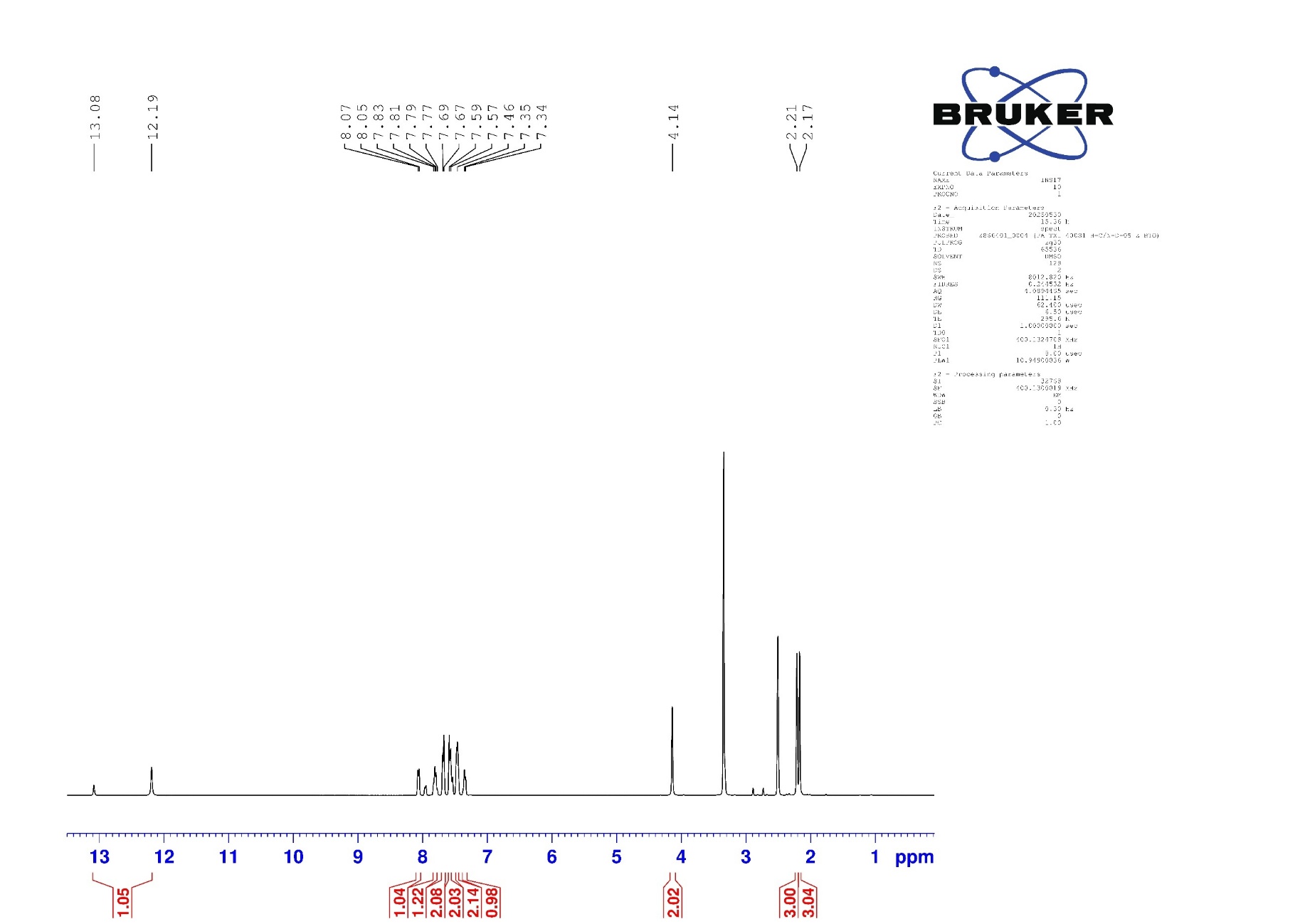


**Figure S36.** ^1^H-NMR spectrum of compound **4j**


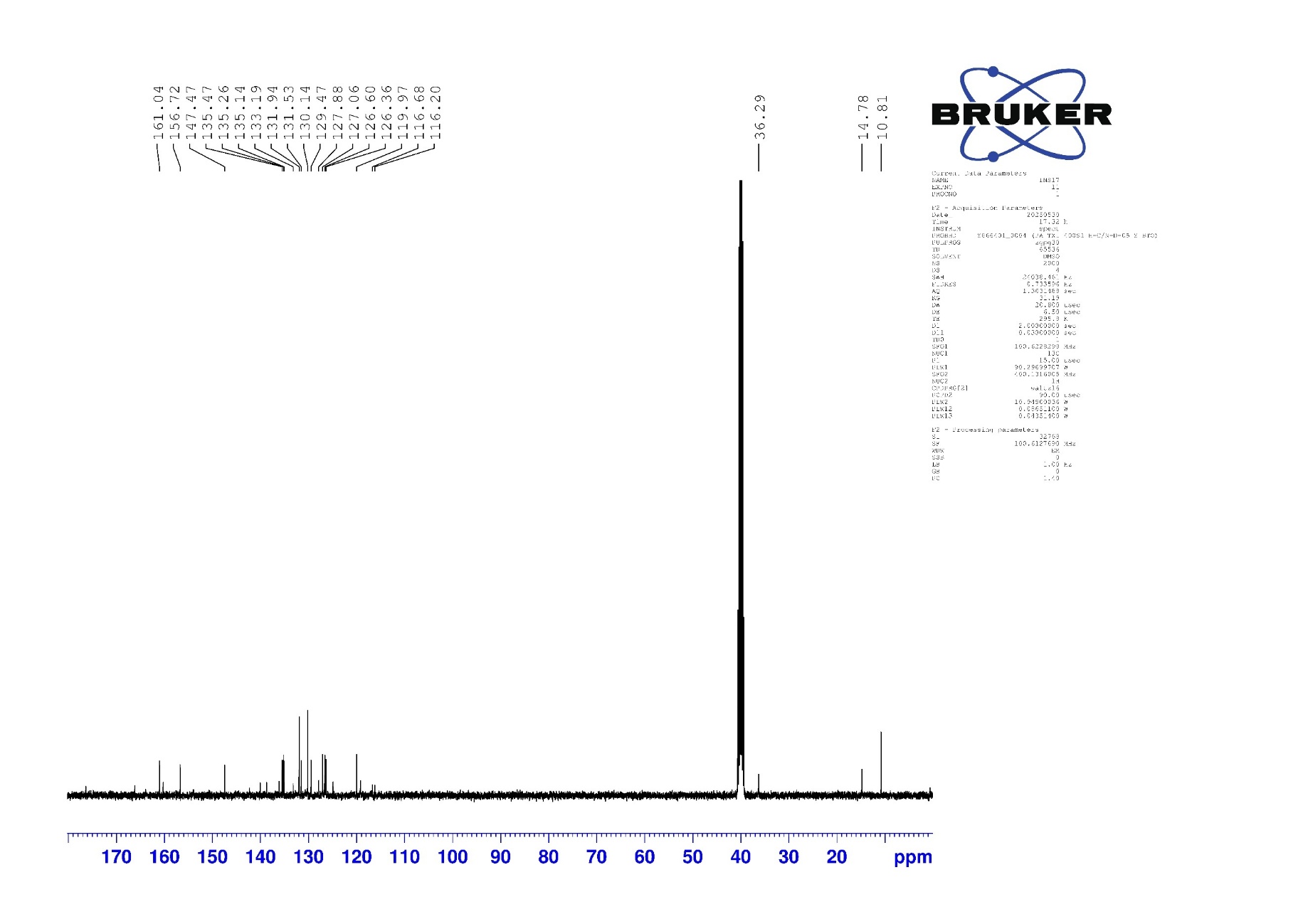


Figure S37. ^13^C-NMR spectrum of compound 4j

***2-((3-(4-Chlorophenyl)-4-oxo-3,4-dihydroquinazoline-2-yl)thio)-N-(6-methylbenzothiazole-2-yl)acetamide (4k)***


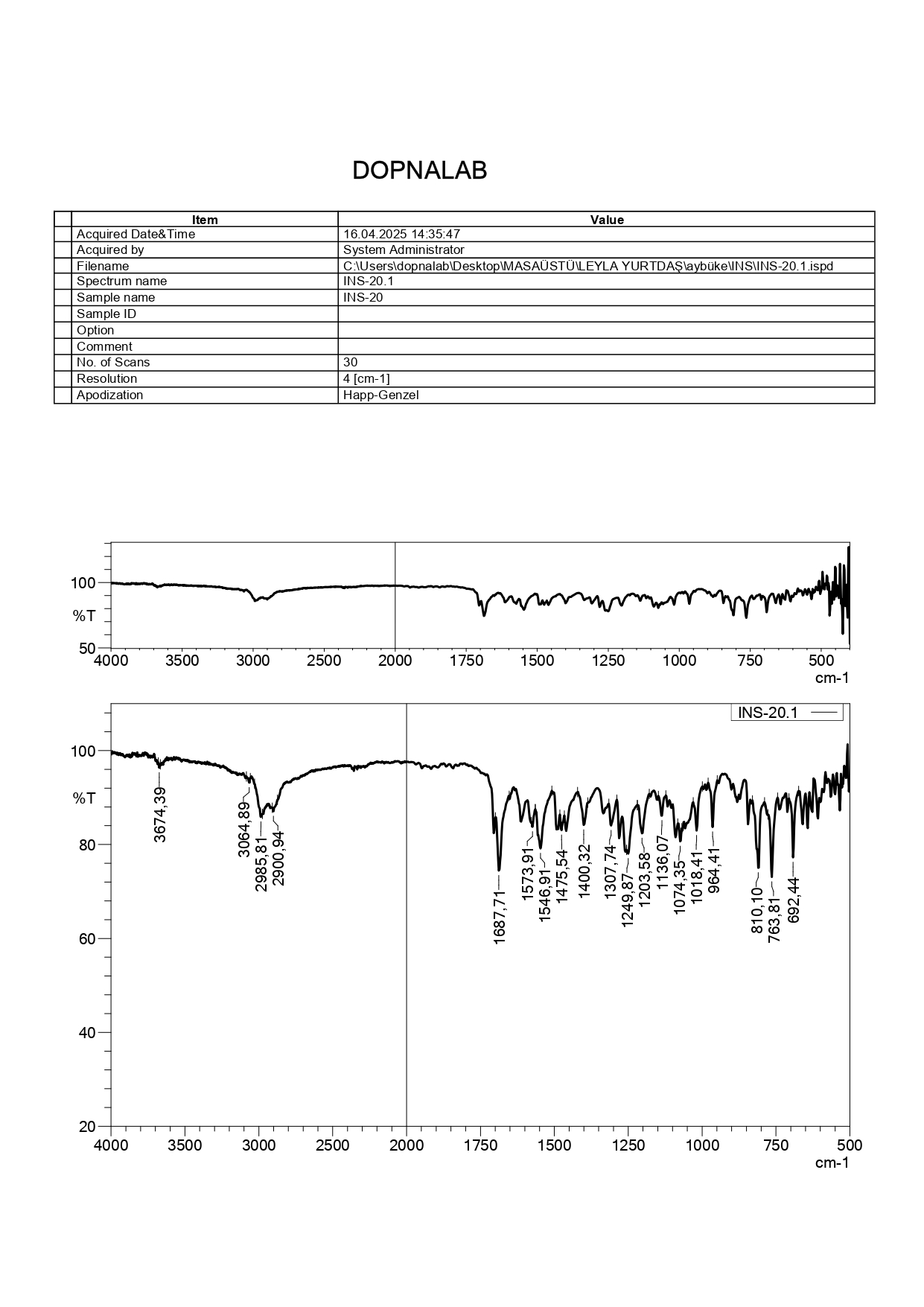


Figure S38. IR spectrum of compound 4k


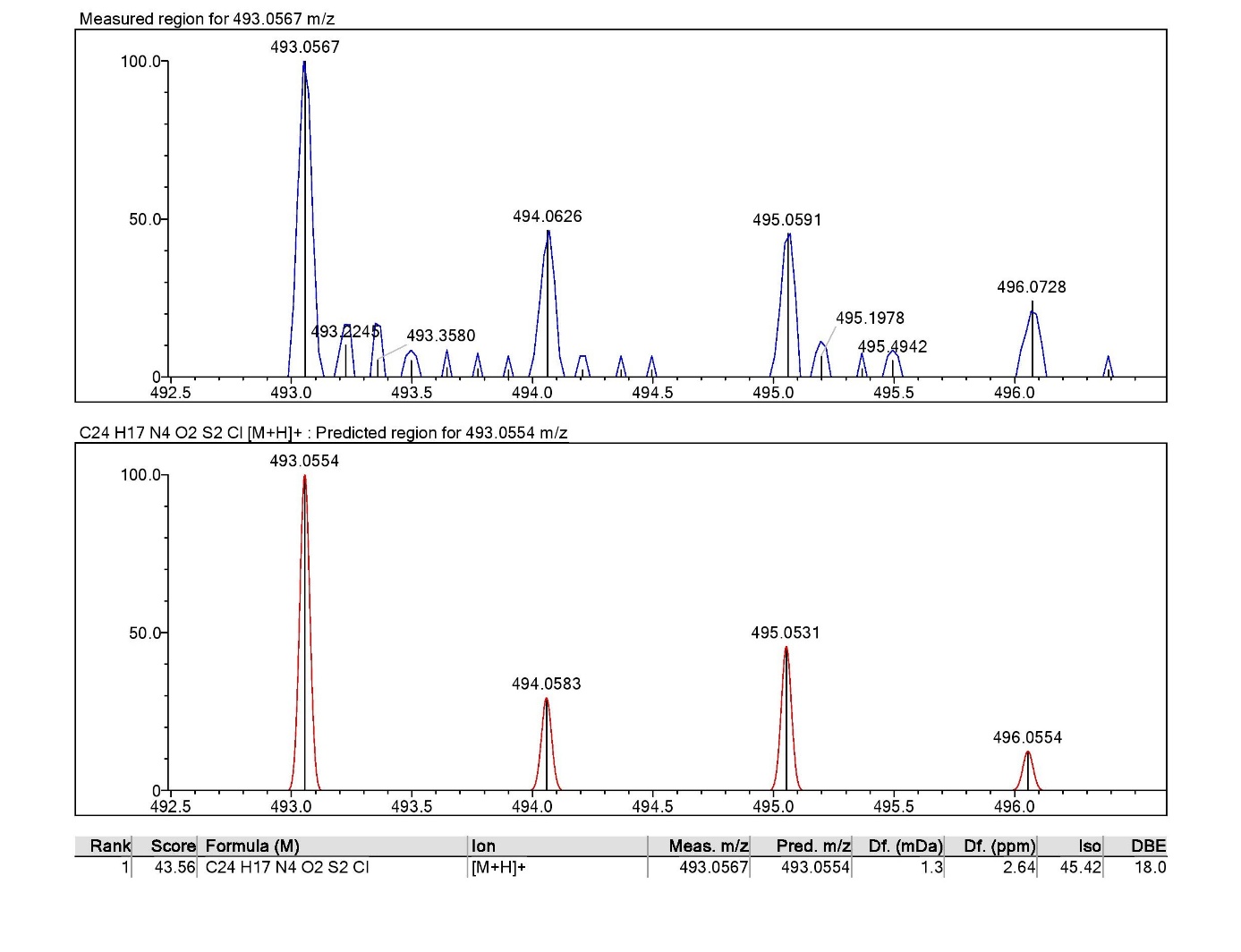


**Figure S39.** HRMS spectrum of compound **4k**

*
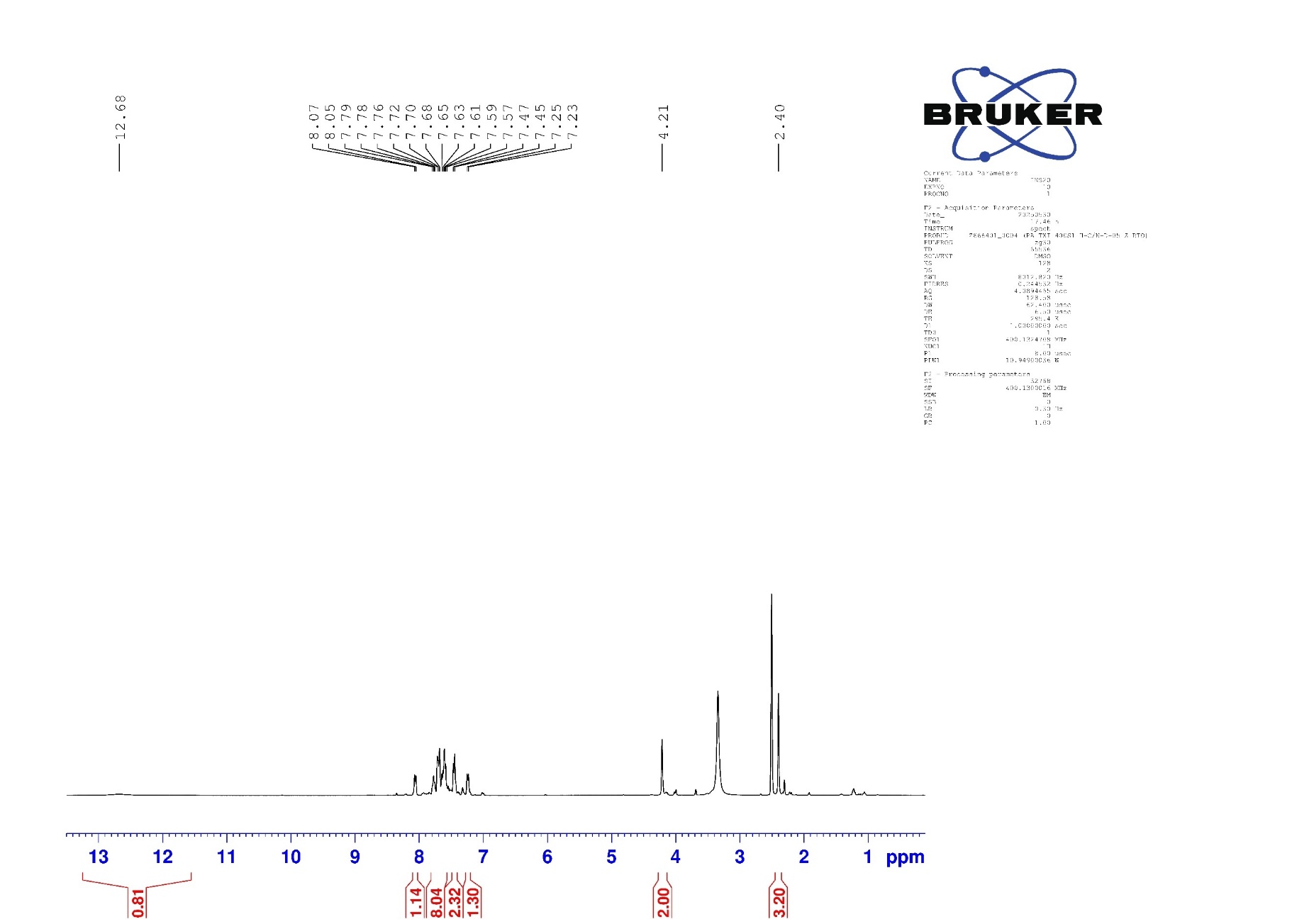
*

**Figure S40.** ^1^H-NMR spectrum of compound **4k**

*
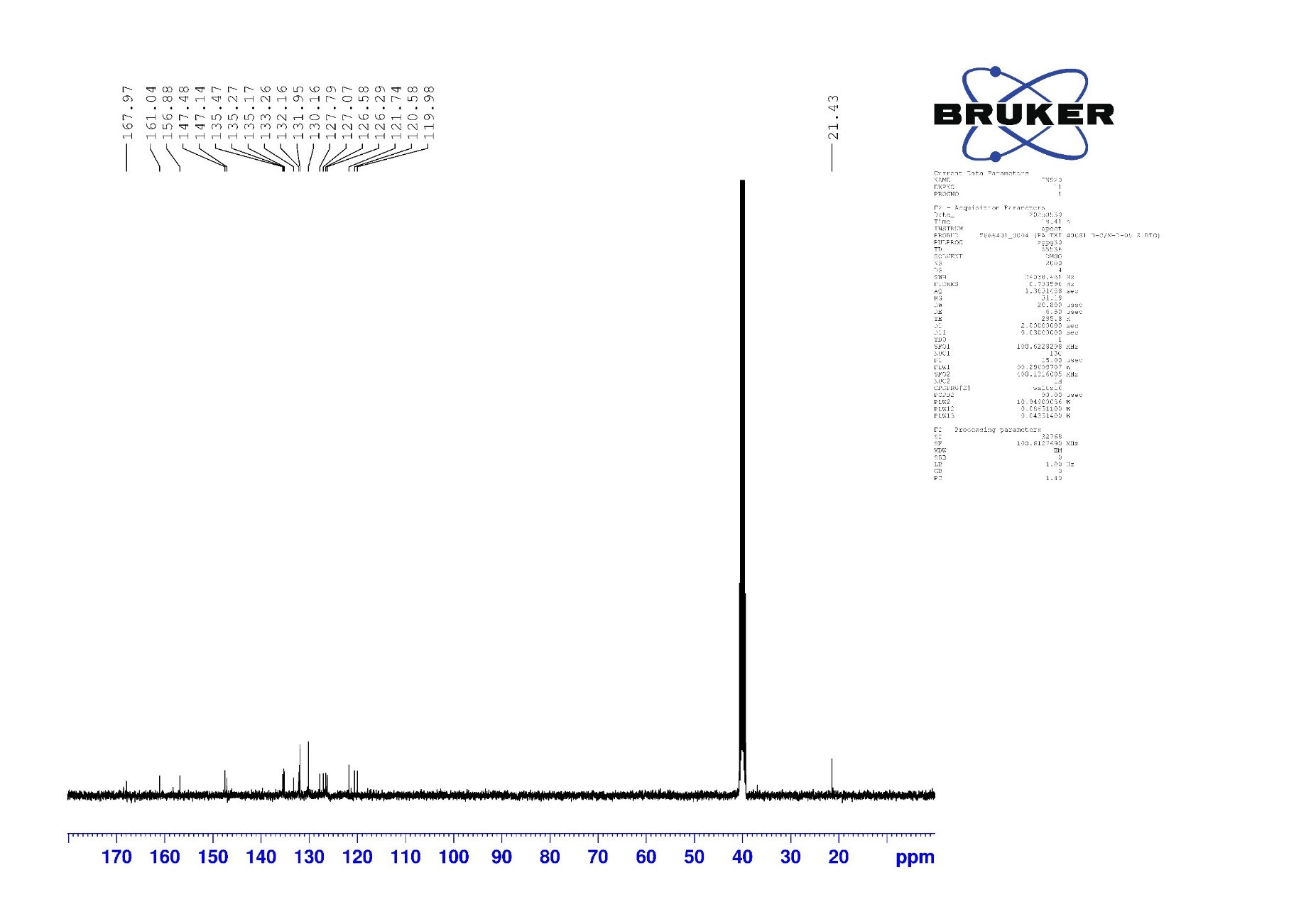
*

Figure S41. ^13^C-NMR spectrum of compound 4k

***2-((3-(4-Chlorophenyl)-4-oxo-3,4-dihydroquinazoline-2-yl)thio)-N-(6-methoxybenzothiazole-2-yl)acetamide (4l)***


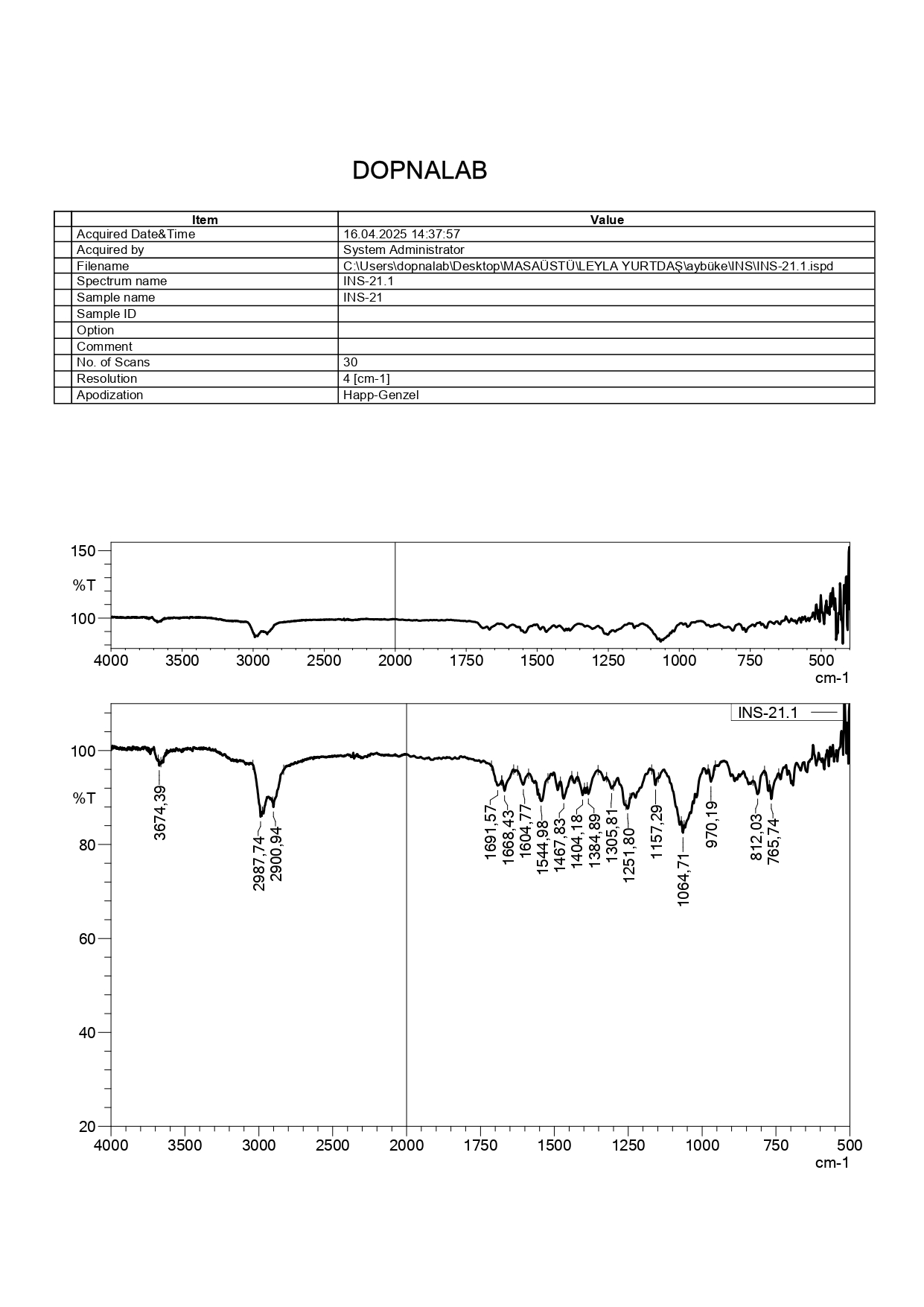


**Figure S42.** IR spectrum of compound **4l**

*
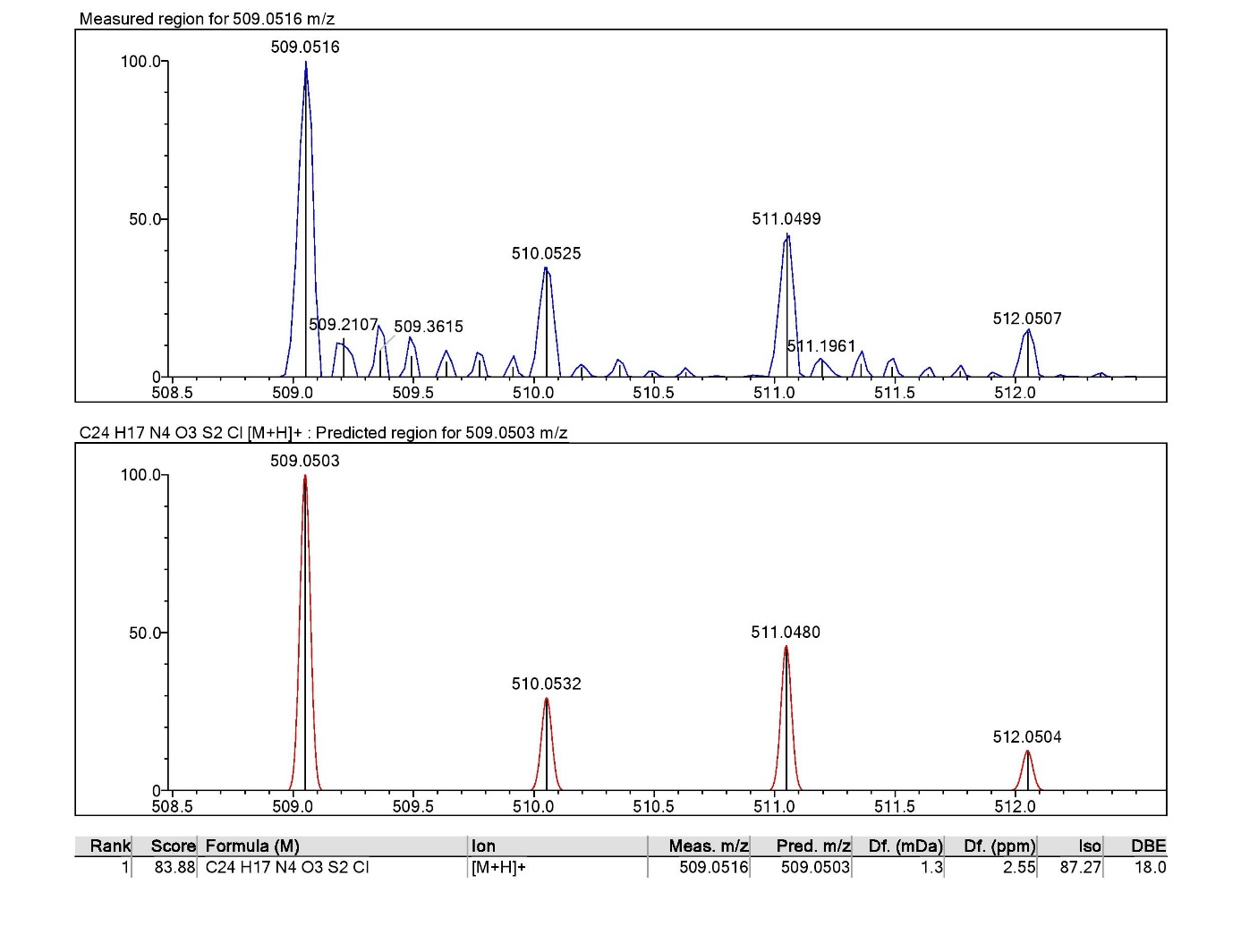
*

**Figure S43.** HRMS spectrum of compound **4l**

*
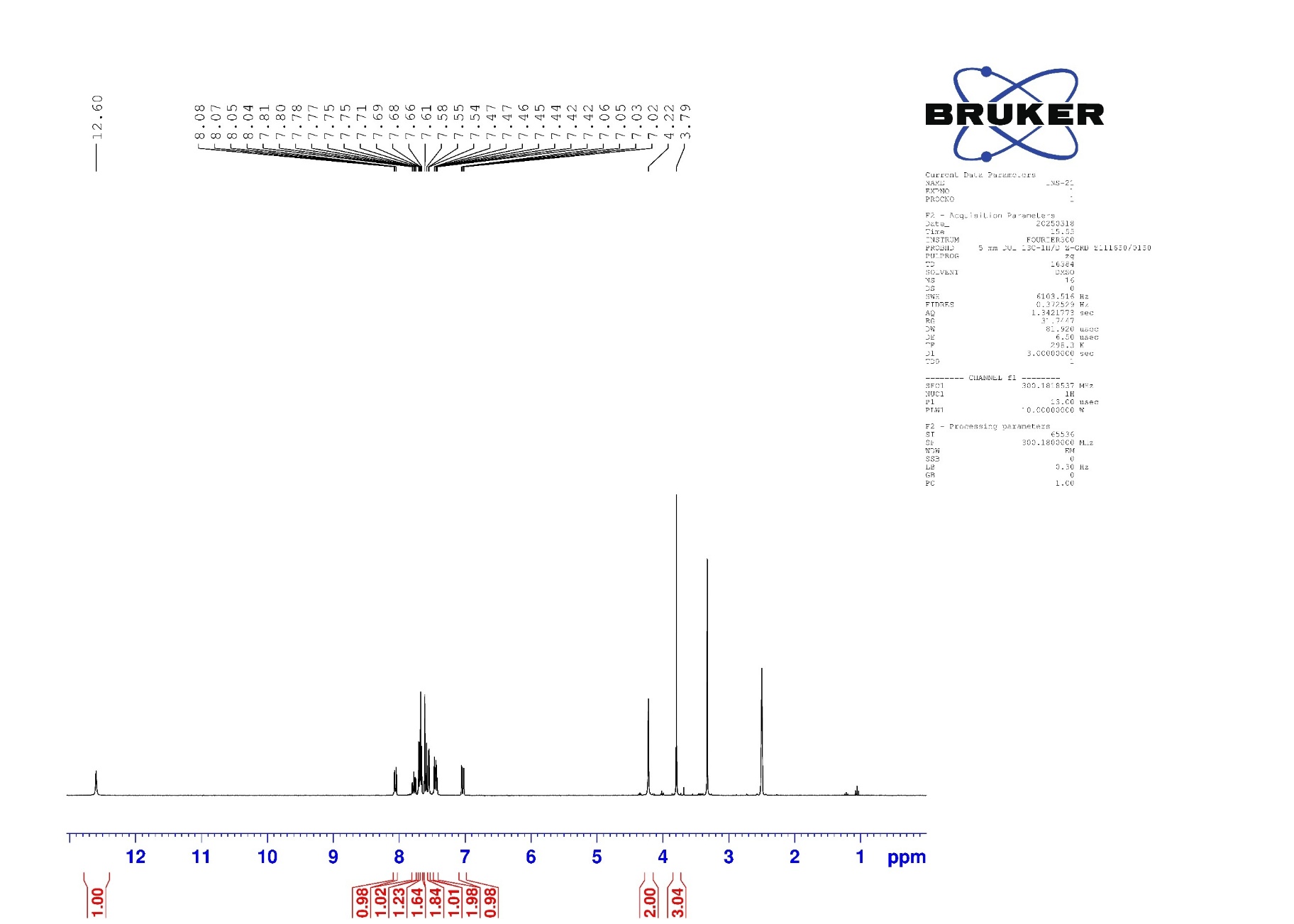
*

**Figure S44.** ^1^H-NMR spectrum of compound **4l**

*
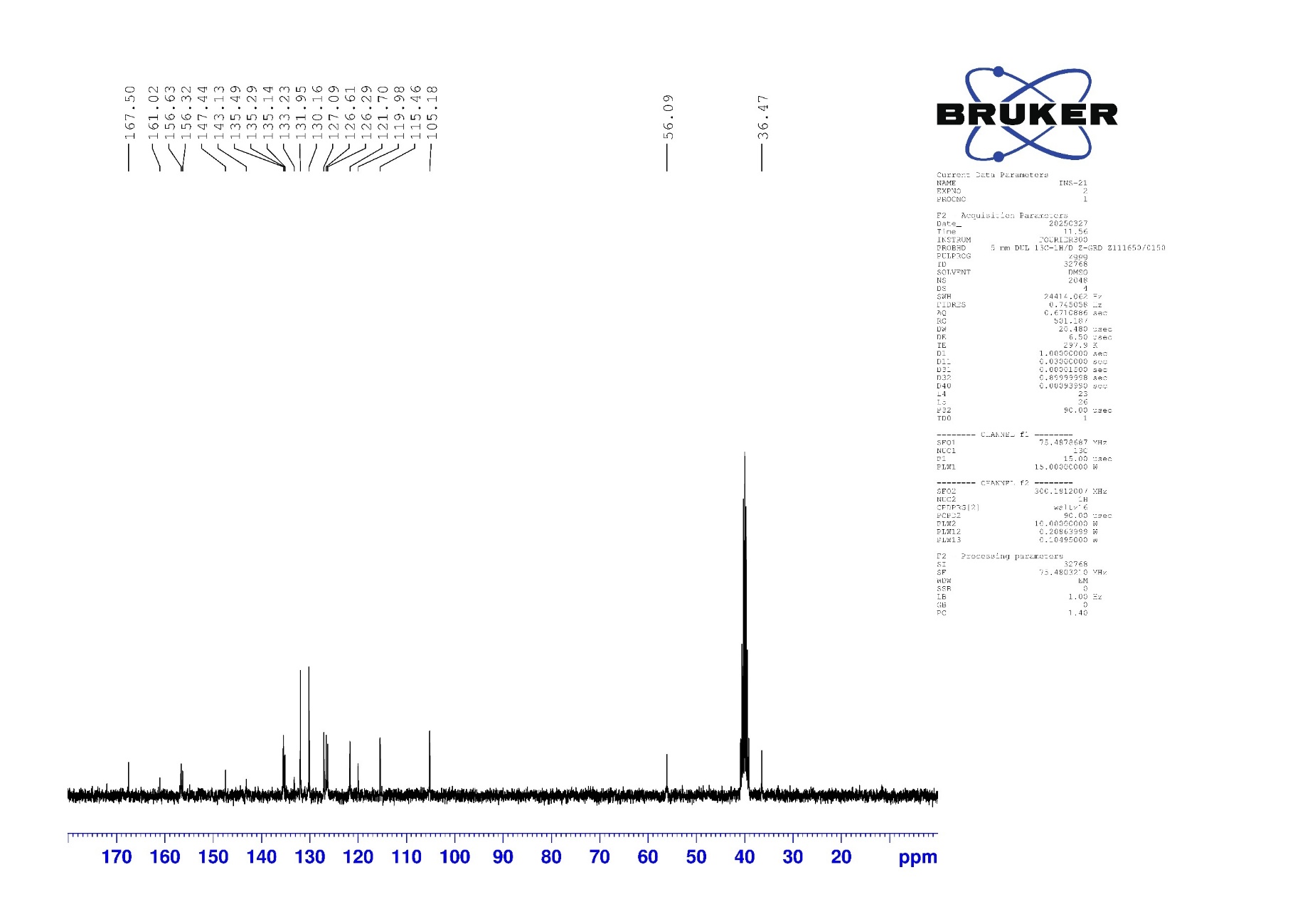
*

Figure S45. ^13^C-NMR spectrum of compound 4l

***N-(6-Chlorobenzothiazole-2-yl)-2-((3-(4-chlorophenyl)-4-oxo-3,4-dihydroquinazoline-2-yl)thio)acetamide (4m)***


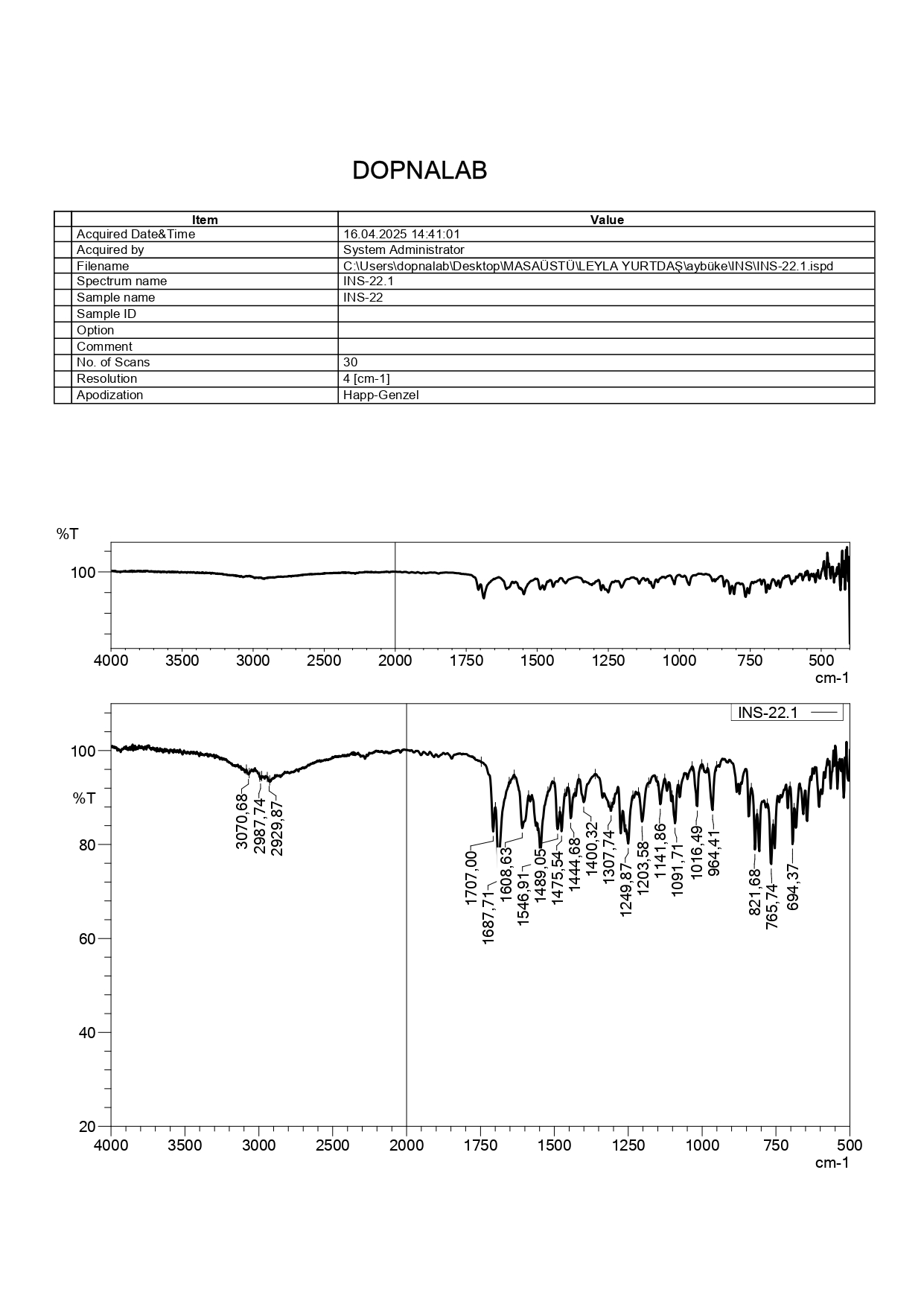


**Figure S46.** IR spectrum of compound **4m**

*
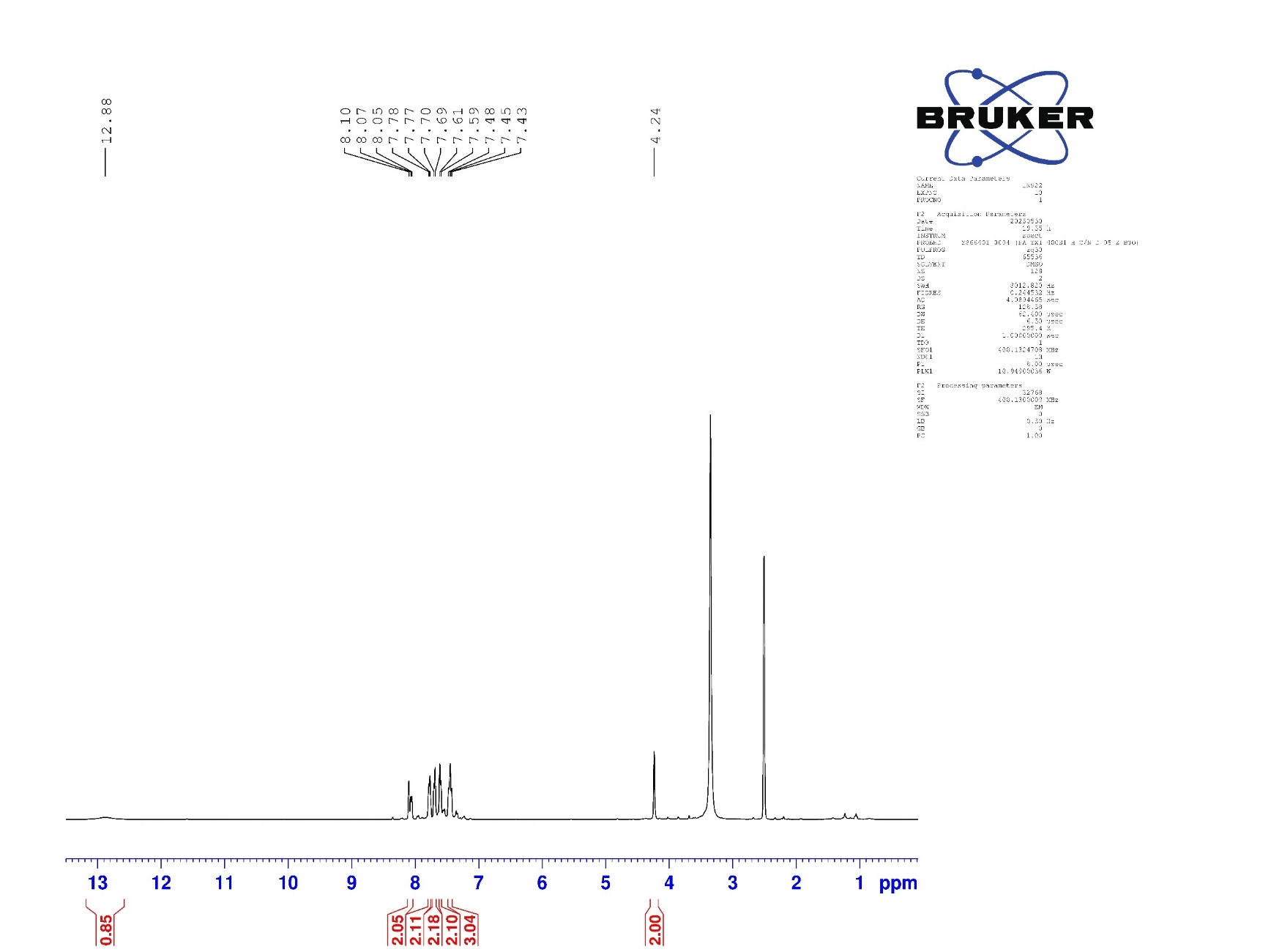
*

**Figure S47.** ^1^H-NMR spectrum of compound **4m**

*
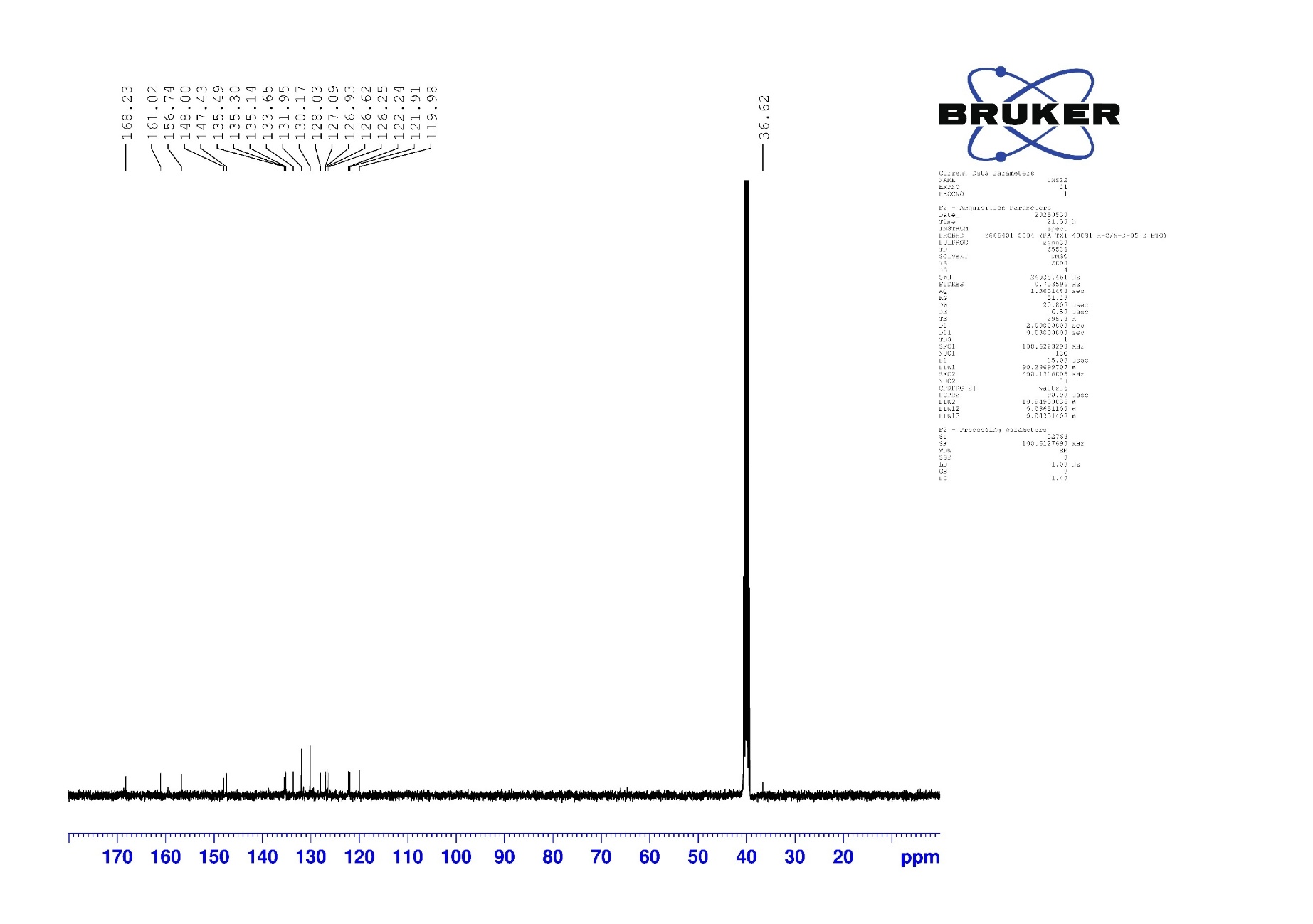
*

Figure S48. ^13^C-NMR spectrum of compound 4m

***2-((3-Benzyl-4-oxo-3,4-dihydroquinazoline-2-yl)thio)-N-(thiazole-2-yl)acetamide (4n)***


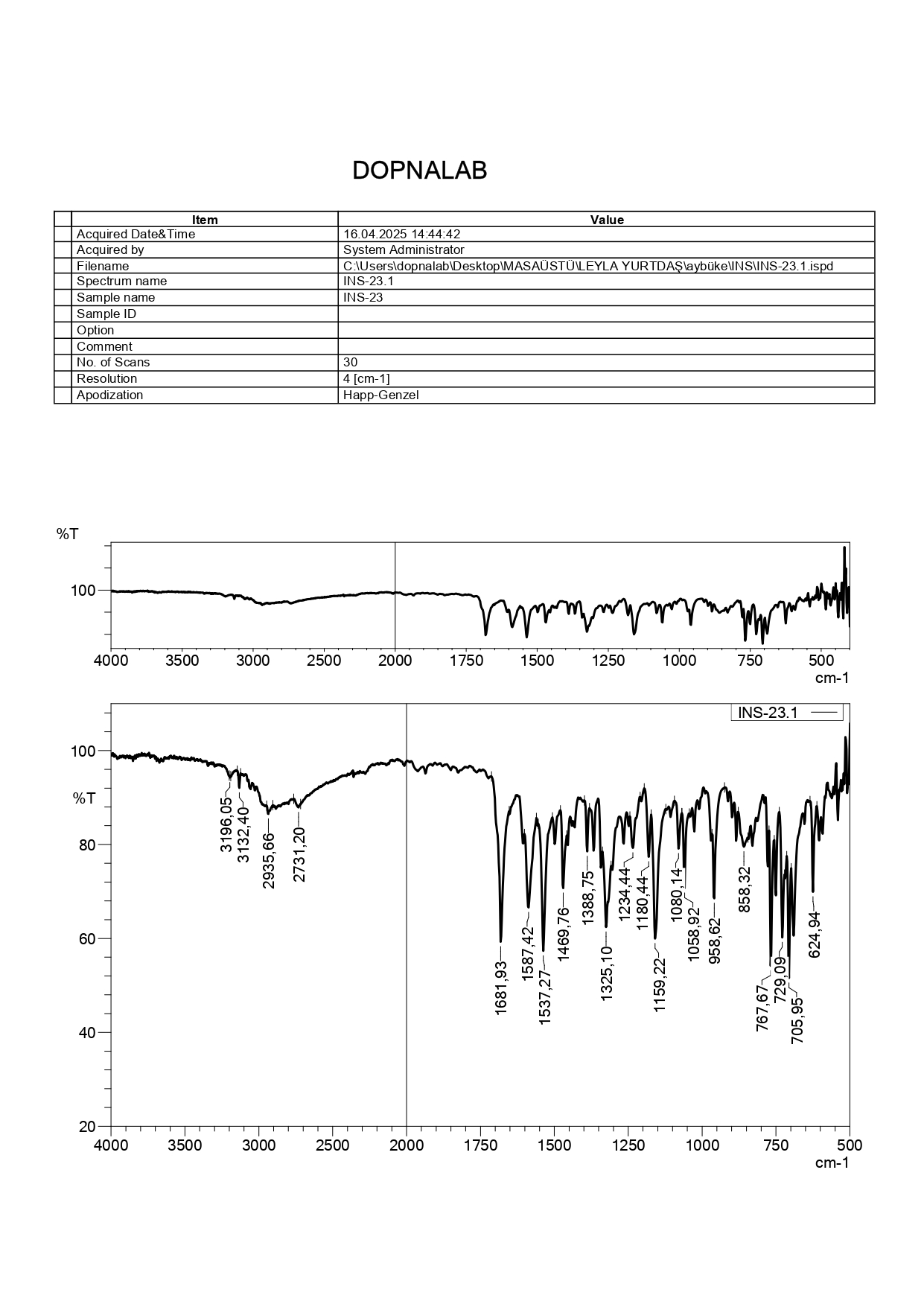


Figure S49. IR spectrum of compound 4n


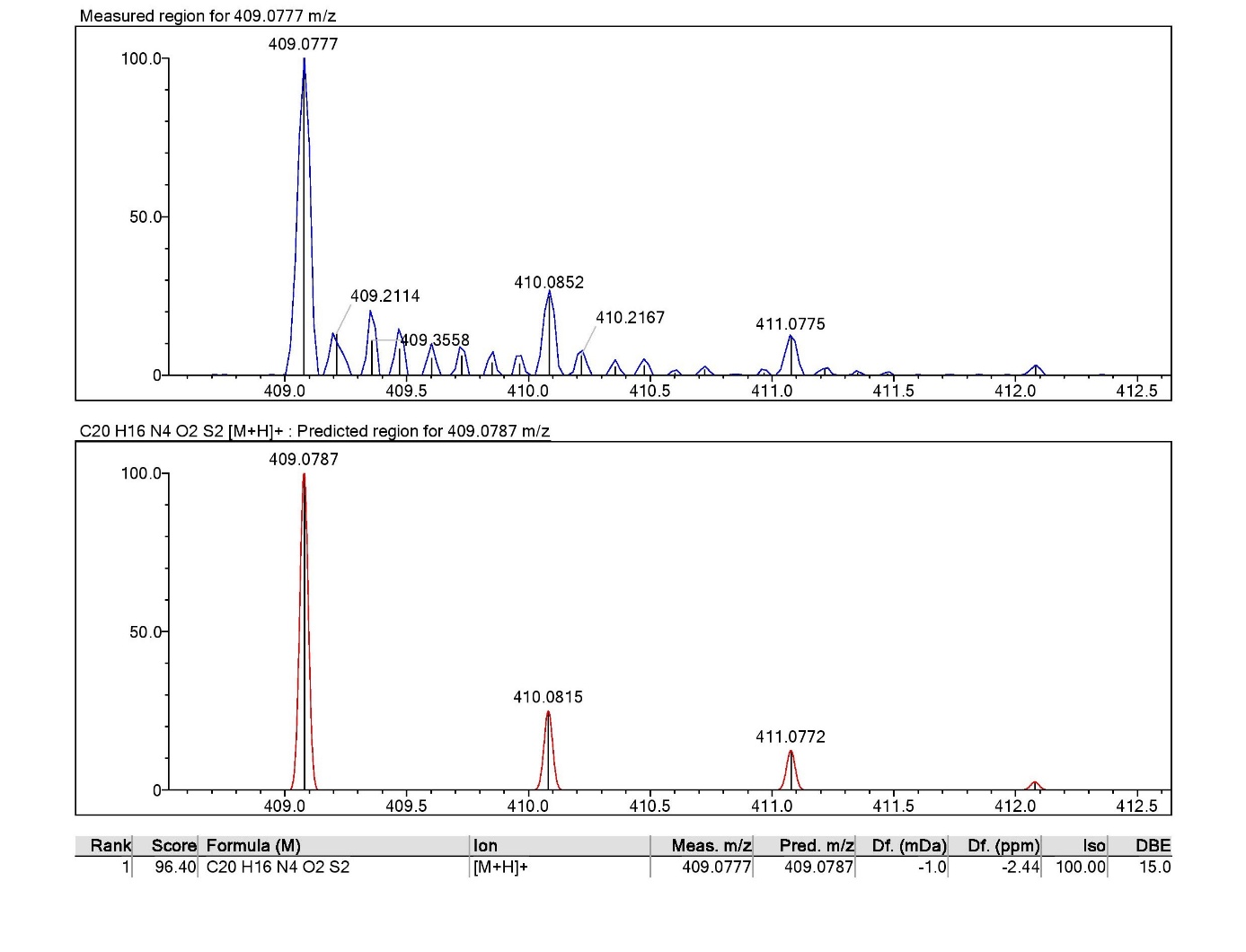


**Figure S50.** HRMS spectrum of compound **4n**

*
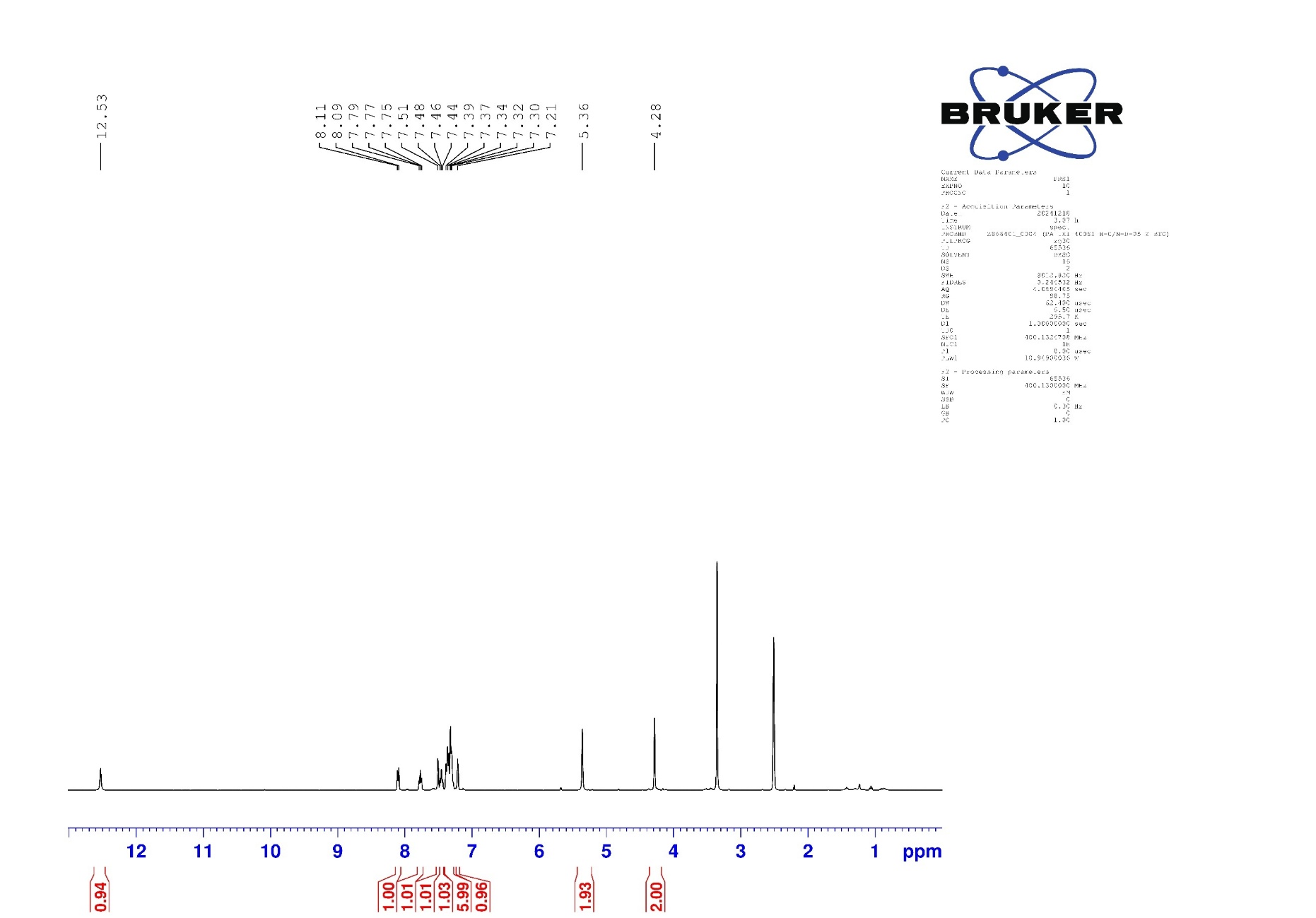
*

Figure S51. ^1^H-NMR spectrum of compound 4n

*
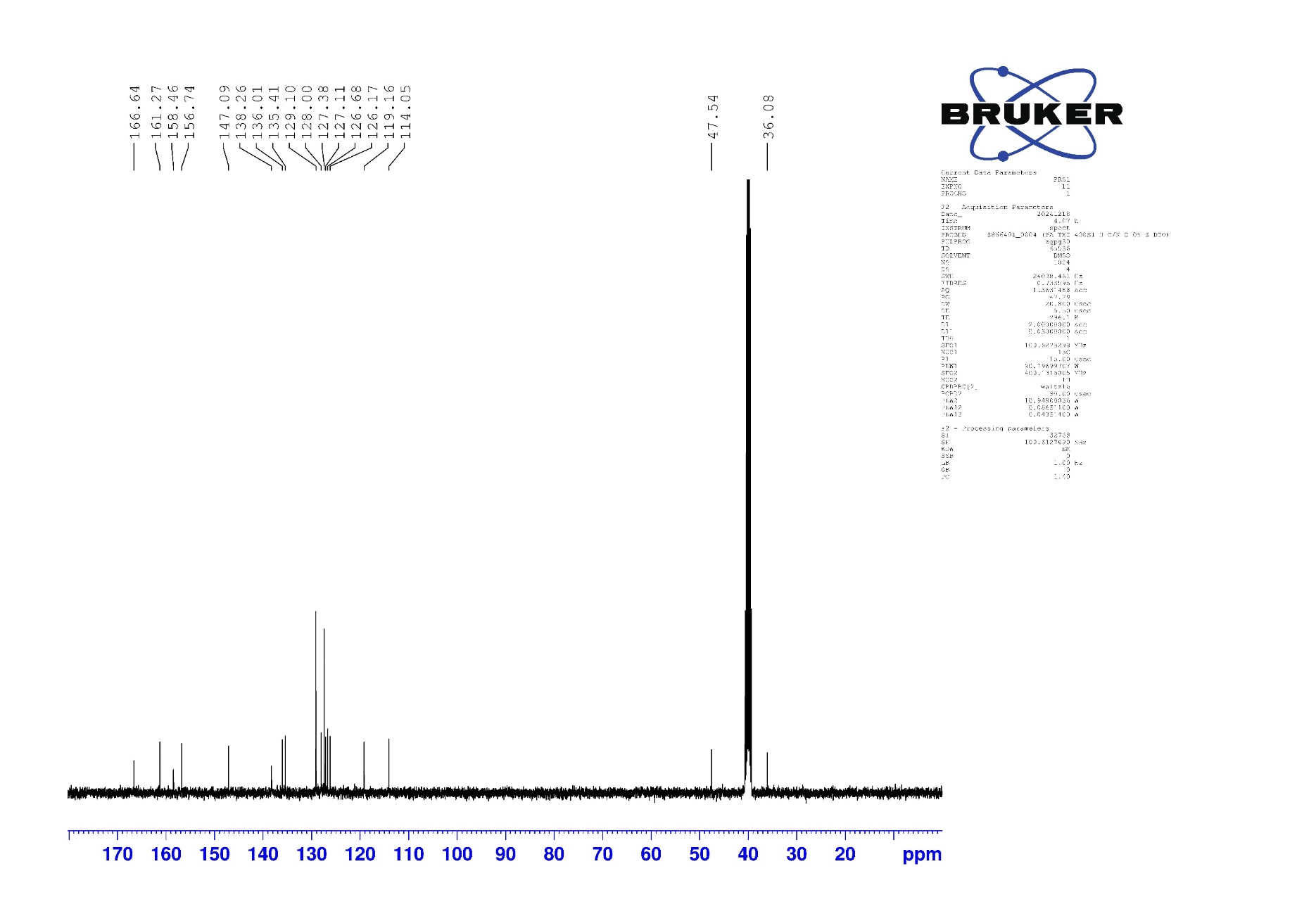
*

Figure S52. ^13^C-NMR spectrum of compound 4n

***2-((3-Benzyl-4-oxo-3,4-dihydroquinazoline-2-yl)thio)-N-(4-flourophenyl)acetamide (4o)***


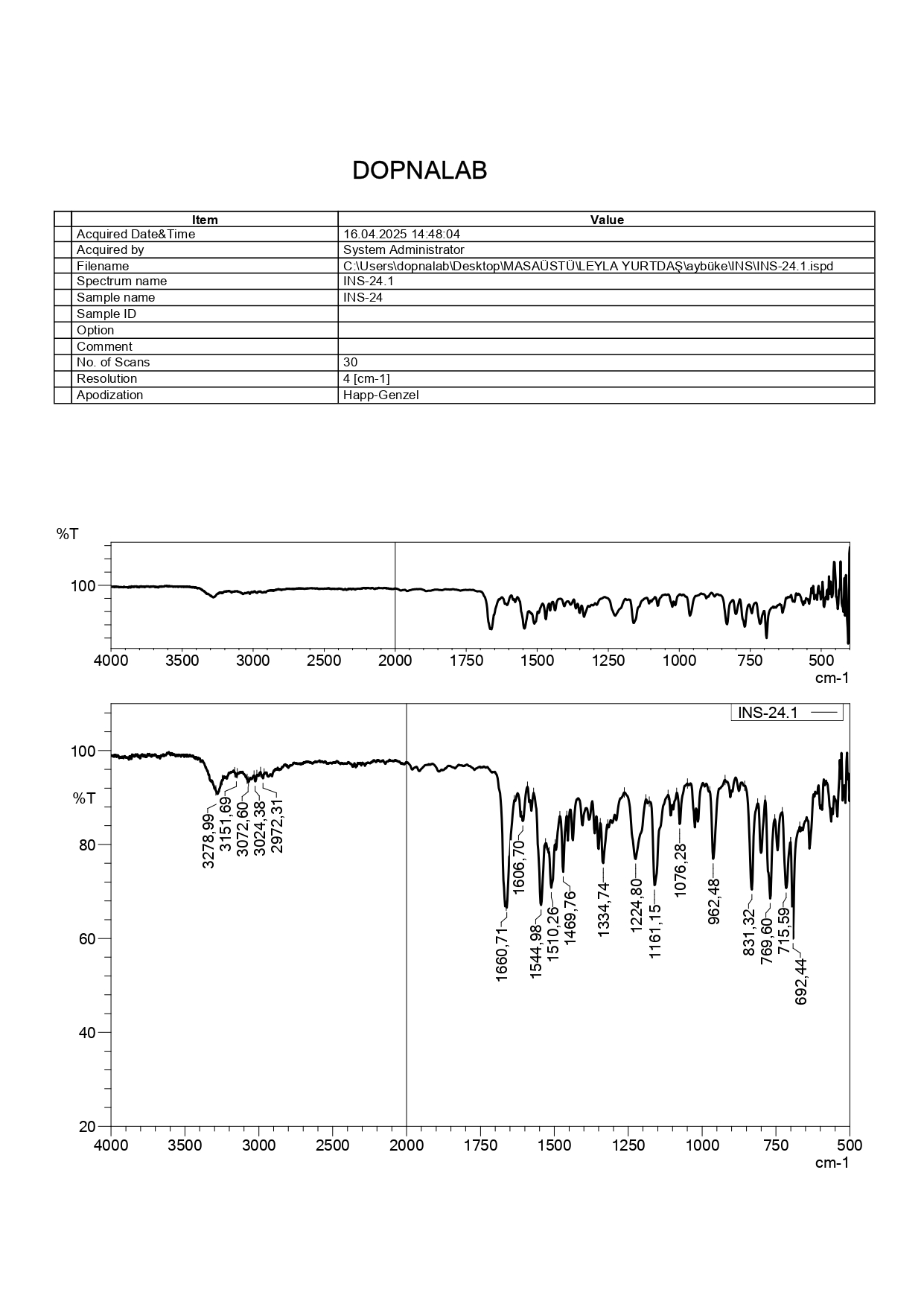


Figure S53. IR spectrum of compound 4o


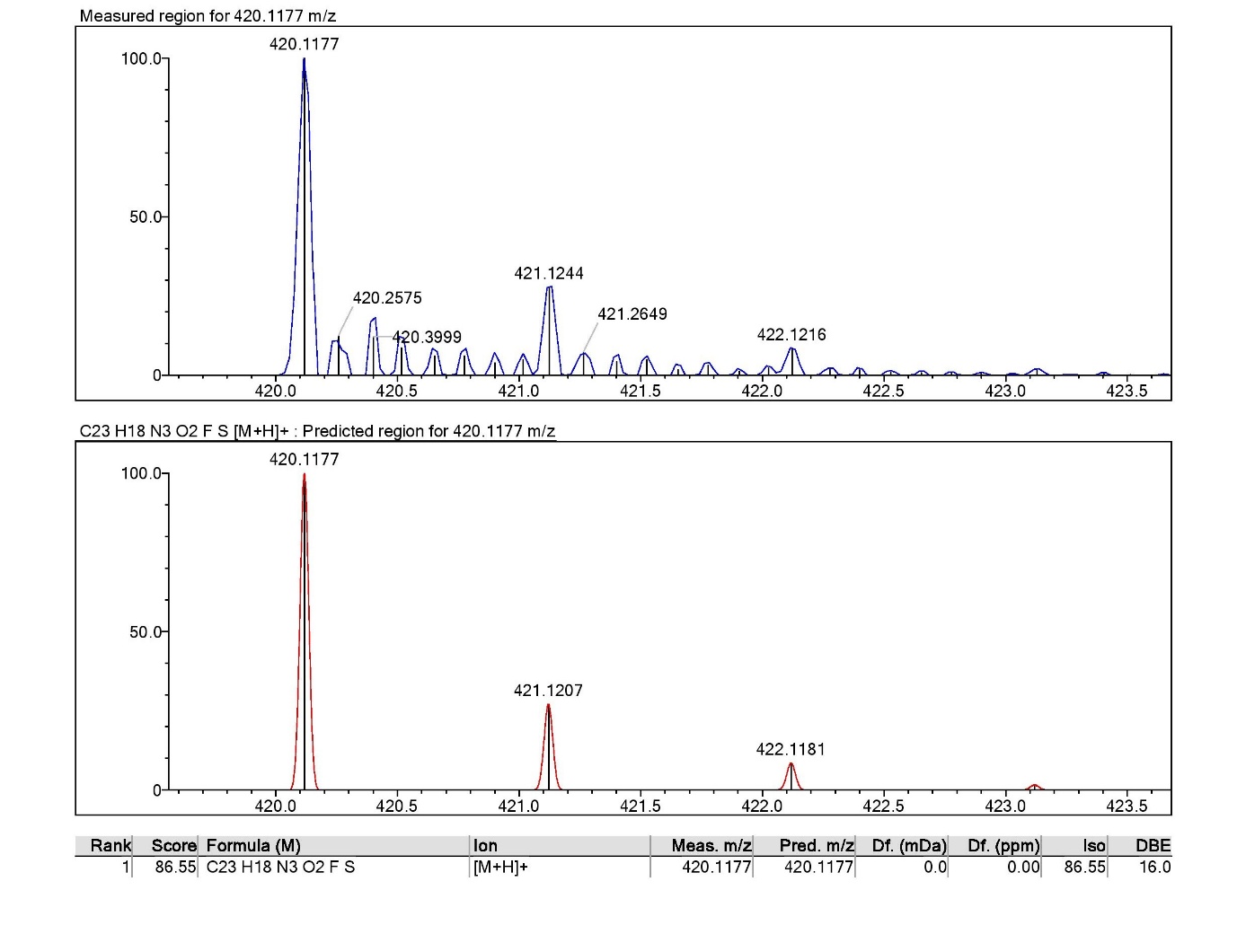


**Figure S54.** HRMS spectrum of compound **4o**

*
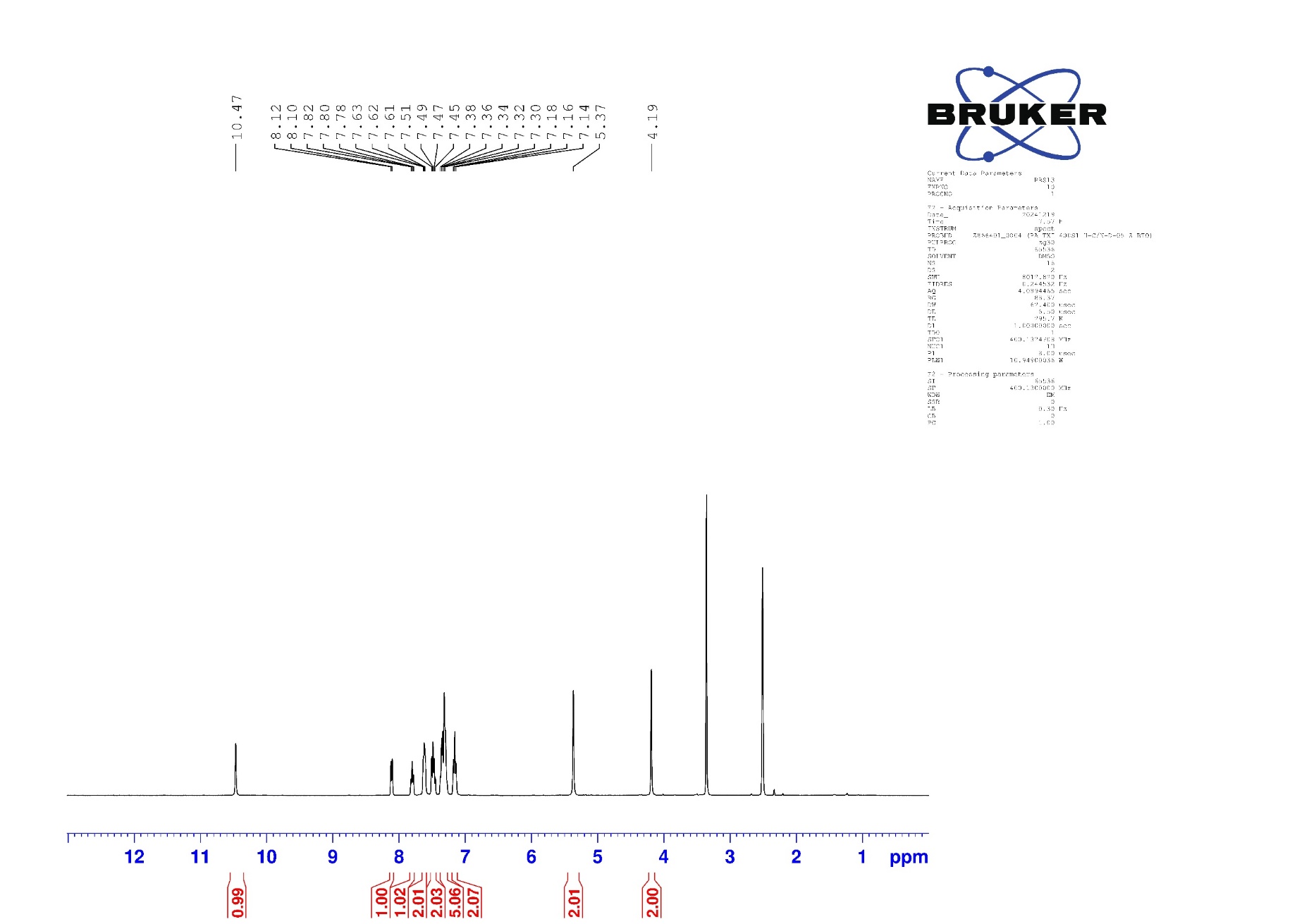
*

Figure S55. ^1^H-NMR spectrum of compound 4o

*
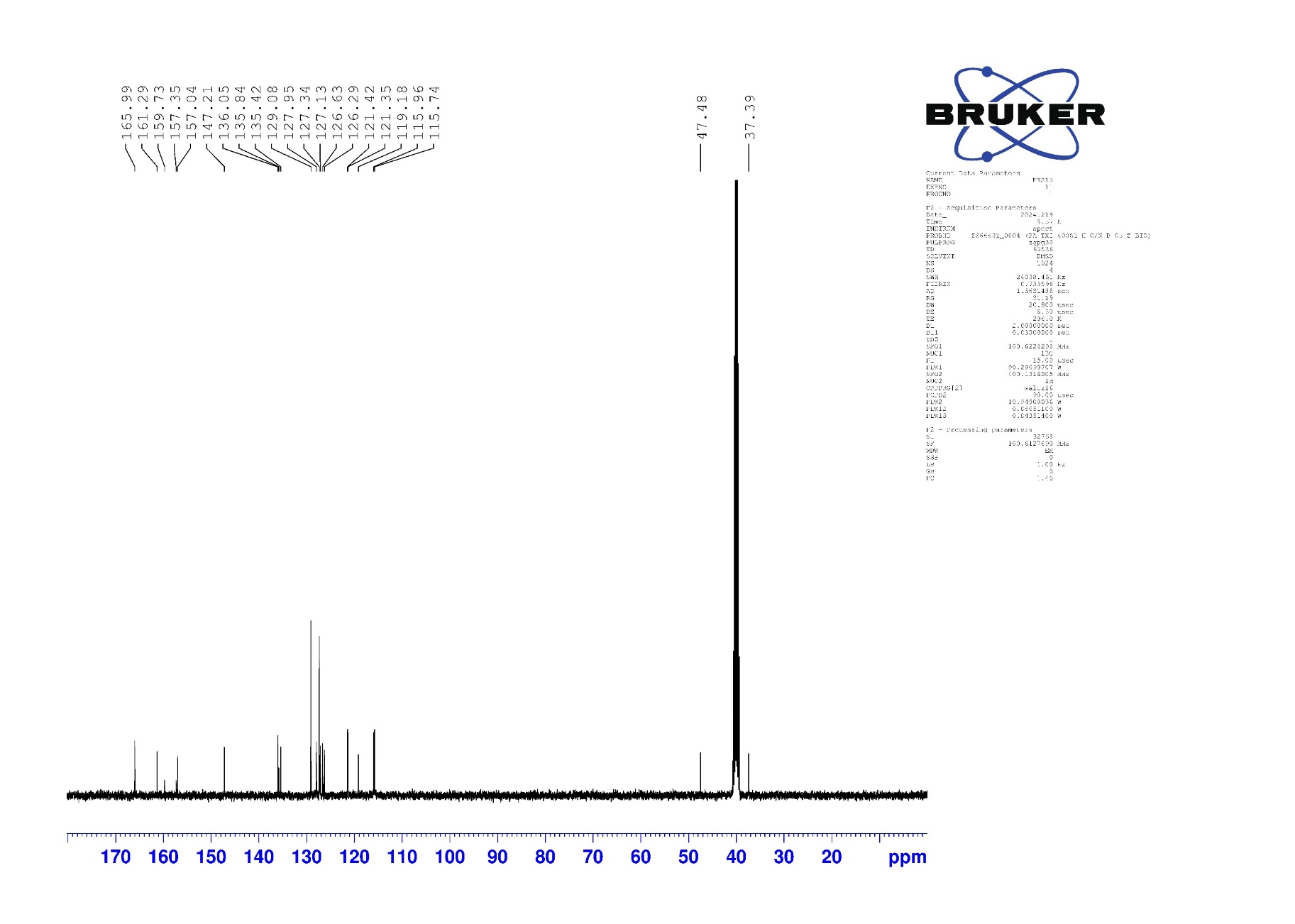
*

Figure S56. ^13^C-NMR spectrum of compound 4o

***2-((3-Benzyl-6-chloro-4-oxo-3,4-dihydroquinazoline-2-yl)thio)-N-(4-flourophenyl)acetamide (4p)***


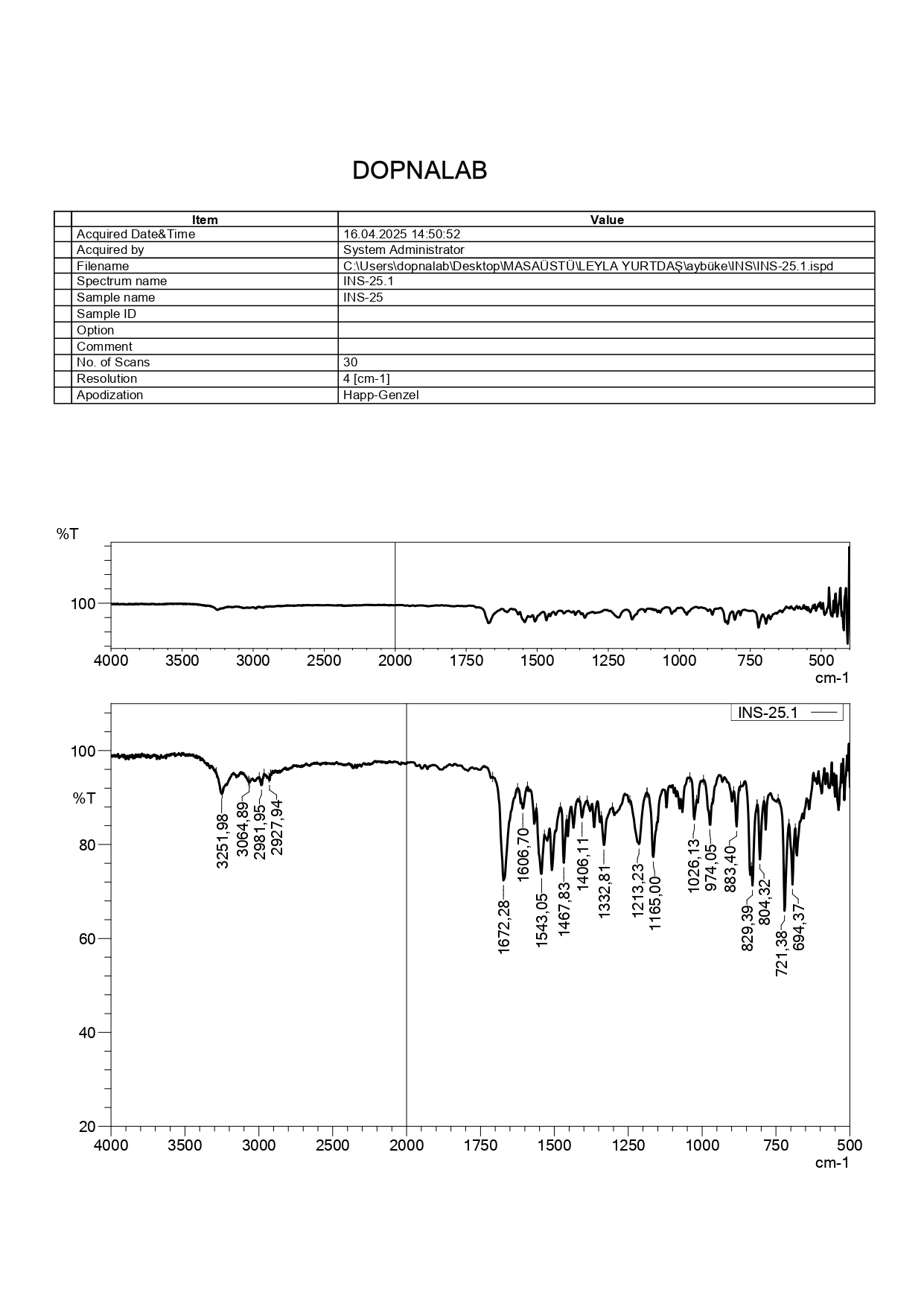


**Figure S57.** IR spectrum of compound **4p**


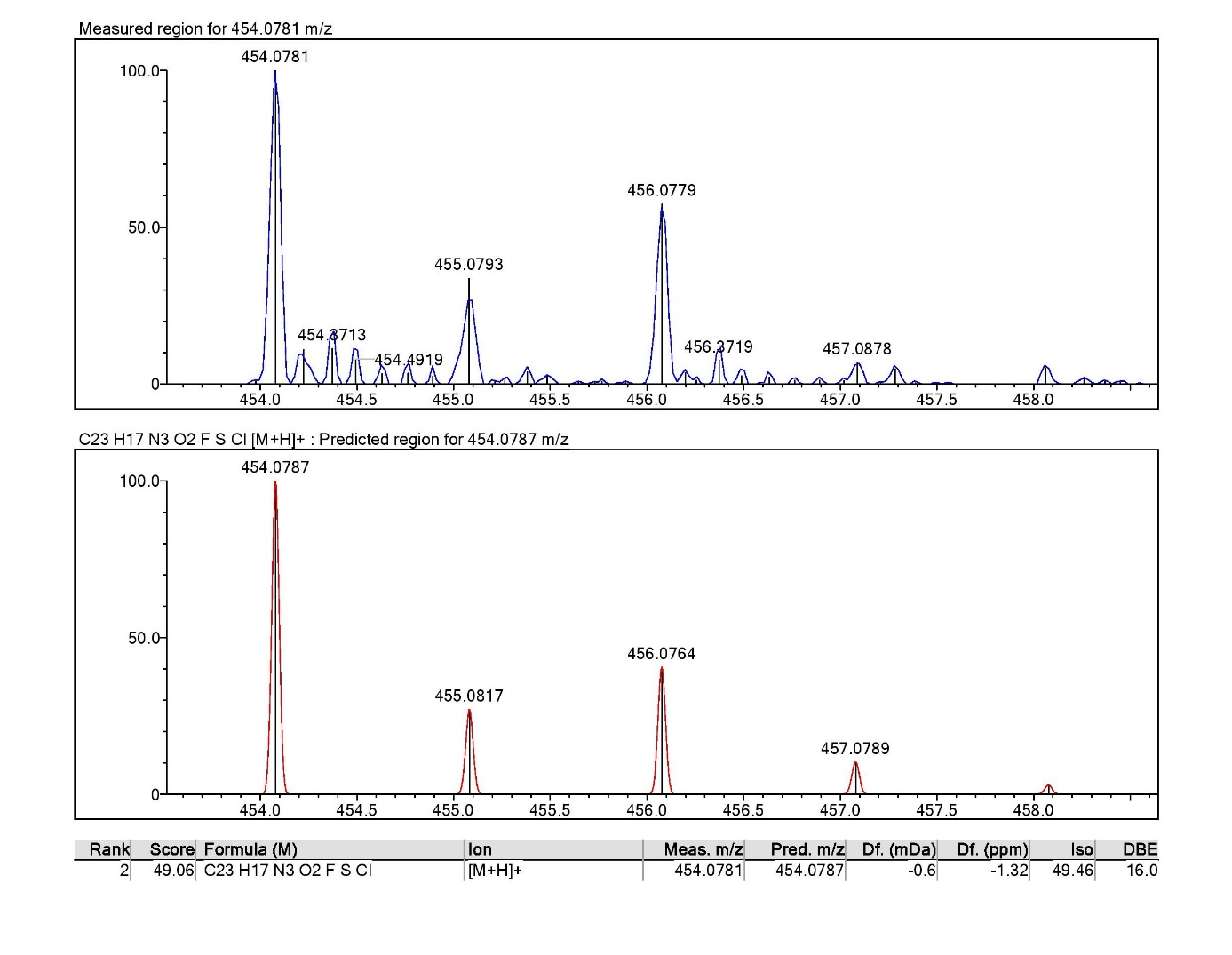


**Figure S58.** HRMS spectrum of compound **4p**

*
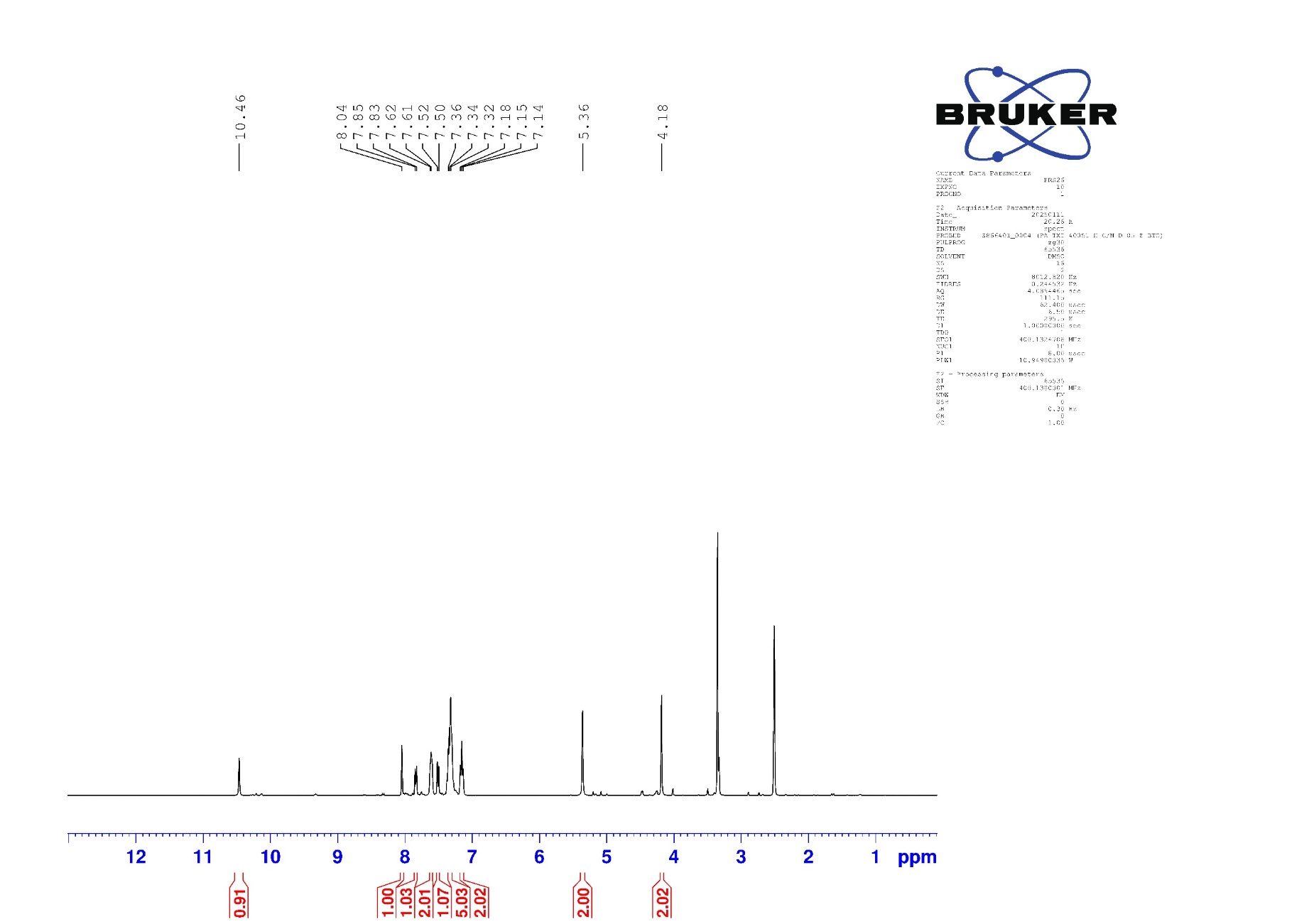
*

Figure S59. ^1^H-NMR spectrum of compound 4p

*
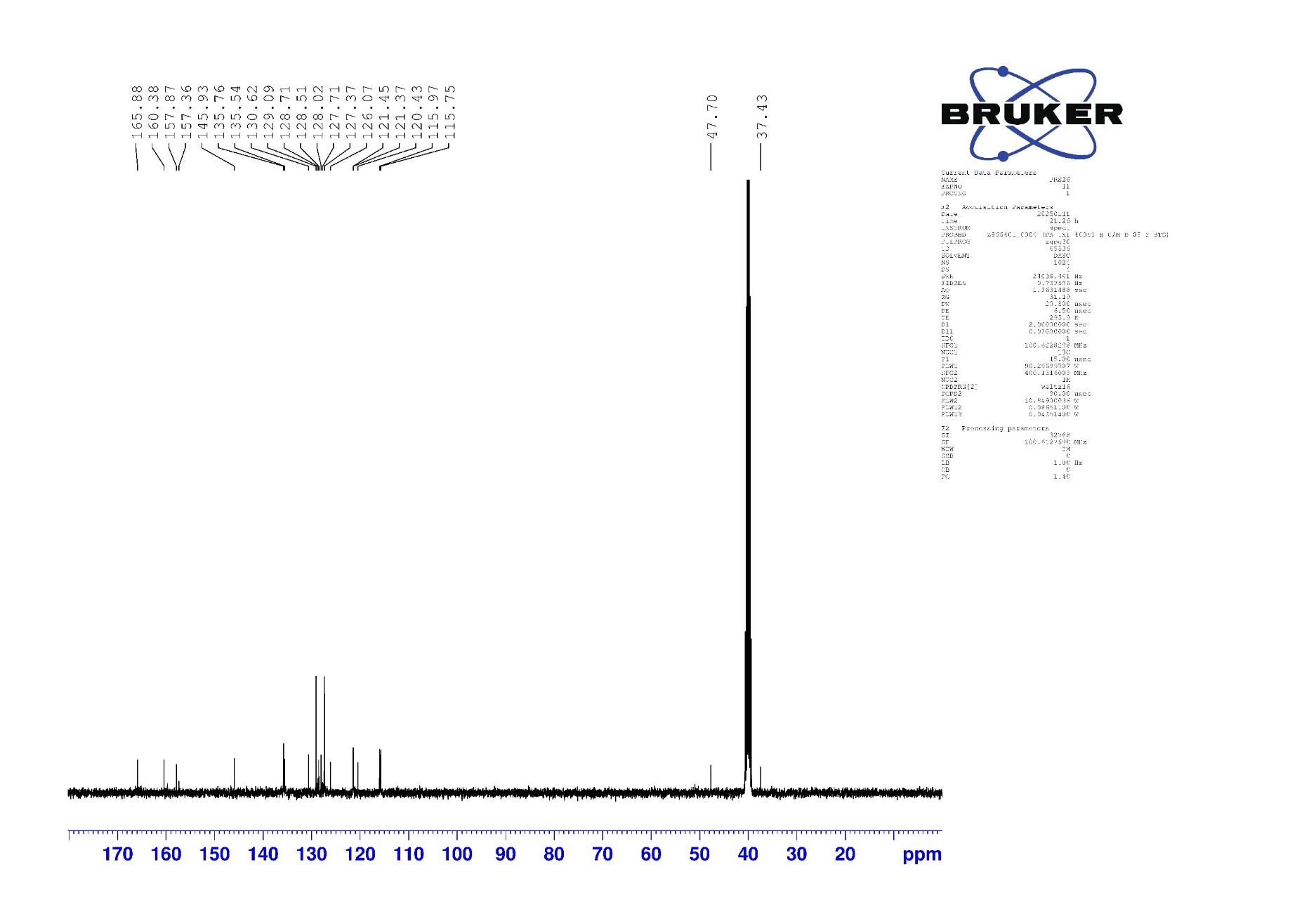
*

Figure S60. ^13^C-NMR spectrum of compound 4p

***2-((3-(4-Flourophenyl)-7-methyl-4-oxo-3,4-dihydroquinazoline-2-yl)thio)-N-(4-methoxyphenyl)acetamide (4r)***

*
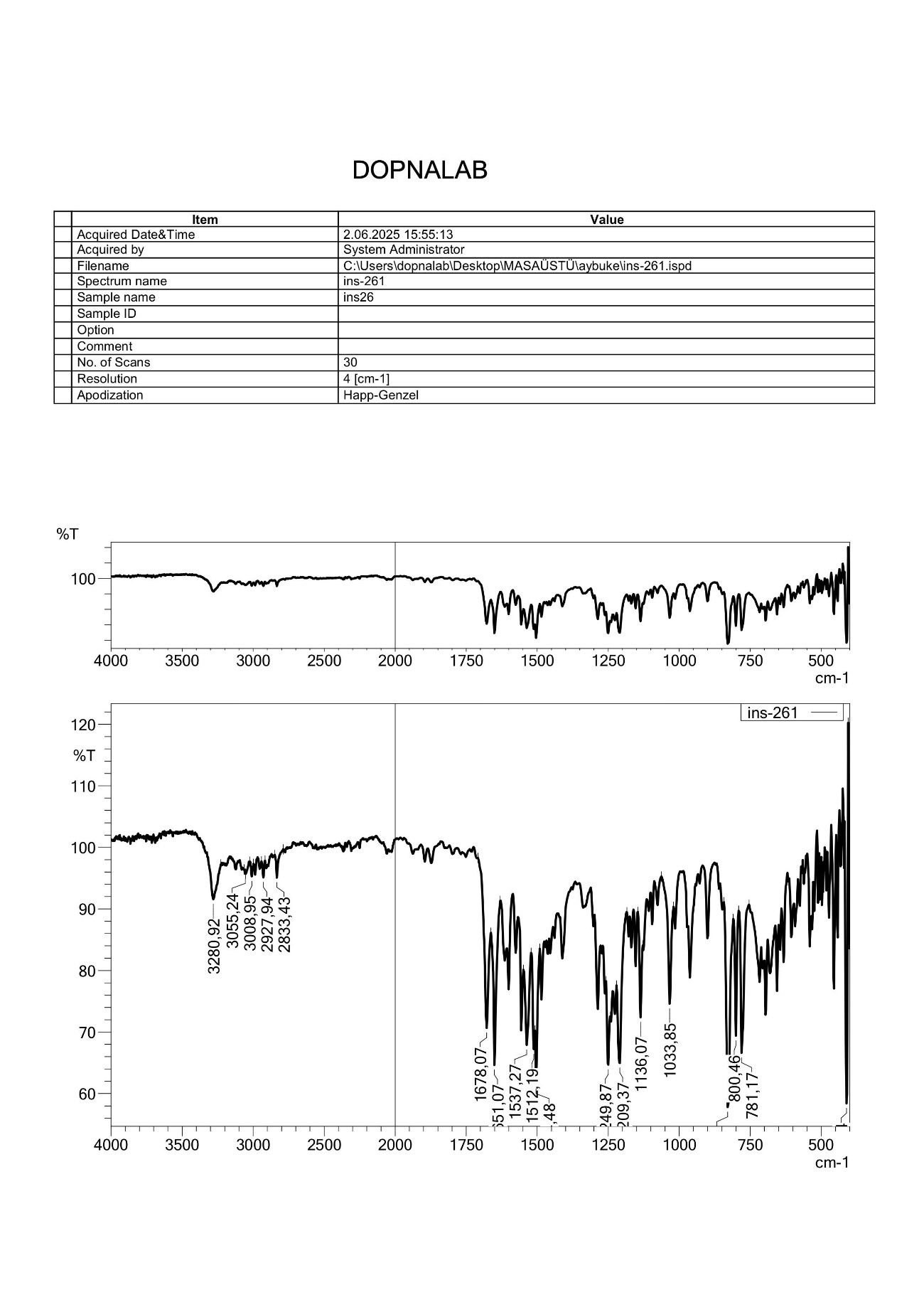
*

Figure S61. IR spectrum of compound 4r

*
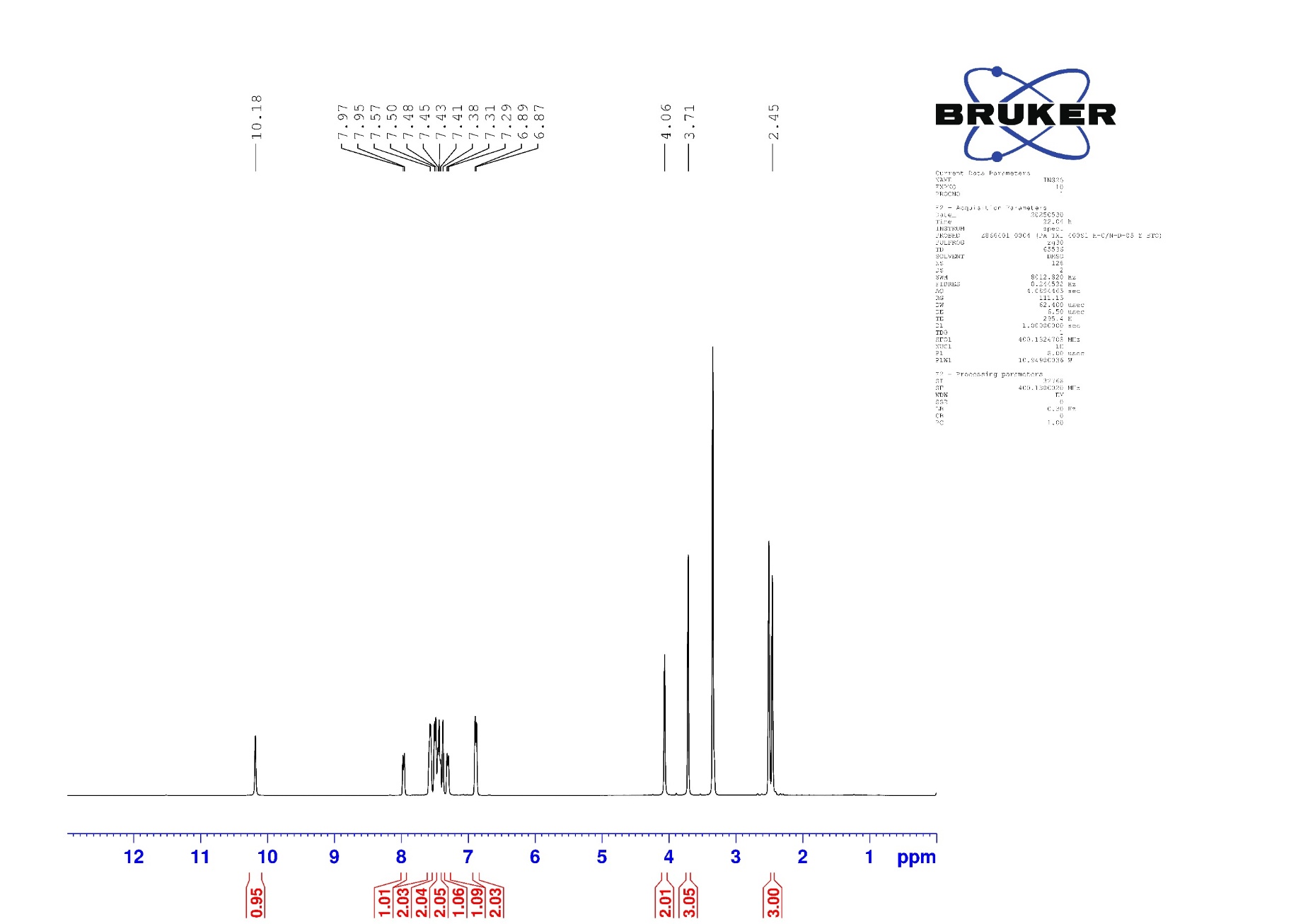
*

Figure S62. ^1^H-NMR spectrum of compound 4r


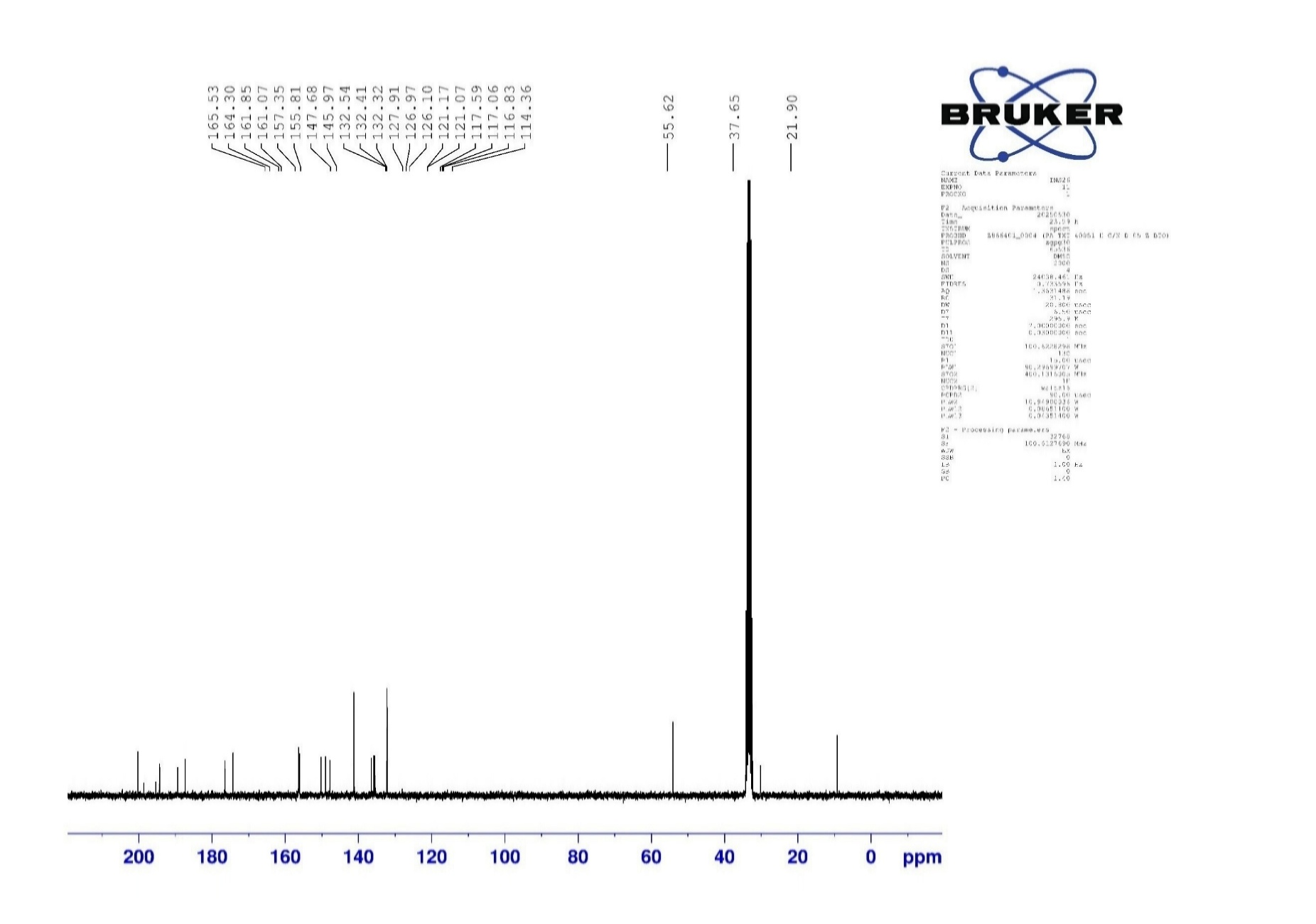


Figure S63. ^13^C-NMR spectrum of compound 4r
